# Localized and Delocalized Nonadiabatic Vibronic Models Unravel Chiroptical Trends and Design Principles in Symmetric π Conjugated Macrocycles

**DOI:** 10.1021/acs.jctc.6c01519

**Published:** 2026-10-19

**Authors:** Francisco García-González, Julia Portela-Pino, Daniel Aranda, José Lorenzo Alonso-Gómez, Fabrizio Santoro

**Affiliations:** † Andalucía Tech, Facultad de Ciencias, Departamento de Química Física, 16752 Universidad de Málaga, 29071, Málaga, Spain; ‡ Instituto Universitario de Materiales y Nanotecnología, IMANA, University of Malaga, Campus de Teatinos 29071, Malaga, Spain; § Department of Organic Chemistry, University of Vigo, 36310, Vigo, Spain; ∥ Department of Physical Chemistry, 16784 Lagoas-Marcosende, 36310, Vigo, Spain; ⊥ Istituto di Chimica dei Composti Organometallici (ICCOM-CNR), 201844 Area della Ricerca del CNR, Via Moruzzi 1, I-56124 Pisa, Italy

## Abstract

The chiroptical response of π-conjugated macrocycles assembled from repeating chromophores can be remarkably intense, yet how it scales with ring size, and what controls it, remains only partly understood. Here we investigate a series of axially chiral diethynylspirobifluorene macrocycles, (*P*_*n*_)-**DES**_*n*_ (*n* = 2, 3, 4; *D*_2_, *D*_3_ and *D*_4_ symmetry), combining accurate nonadiabatic quantum dynamics with two complementary linear vibronic coupling Hamiltonians: a delocalized model, whose diabatic states span the whole macrocycle, and a localized, exciton-like model built from the excitations of the individual fragments. Both reproduce the experimental one-photon absorption and electronic circular dichroism (ECD) spectra and reveal that nonadiabatic interactions, conical intersections and (pseudo-)­Jahn–Teller couplings govern their shapes; a hierarchical representation of the Hamiltonian keeps the simulations tractable, even for (*P*_4_)-**DES**_4_, with more than 500 vibrational modes. A simple symmetry analysis bridges the two pictures, tracing every coupling back to the single excited-state gradient of the monomer and partitions the magnetic transition dipole into an intrinsic contribution, from the strained monomer and an extrinsic, excitonic one. This decomposition rationalizes the observed trends explaining why the ECD grows super-linearly with ring size. As a result, the response crosses over from strain-dominated chirality in (*P*_2_)-**DES**_**2**_ to excitonic chirality in (*P*_4_)-**DES**_4_ at a nearly constant dissymmetry factor. Crucially, the excitonic rotatory strengths form a nearly conservative couplet whose oppositely signed branches partly cancel, and their splitting (0.12–0.16 eV) is comparable to the width of the vibronic progressions. The intrinsic band shape of the monomer, not the splitting alone, therefore sets how much ECD survives. We translate these findings into geometric, excitonic and vibronic design principles for *g*-factor improved symmetric chiral macrocycles, suggesting that our proposed computational approach can be a fast screening tool for new designed macrocycles before synthesis.

## Graphical abstract

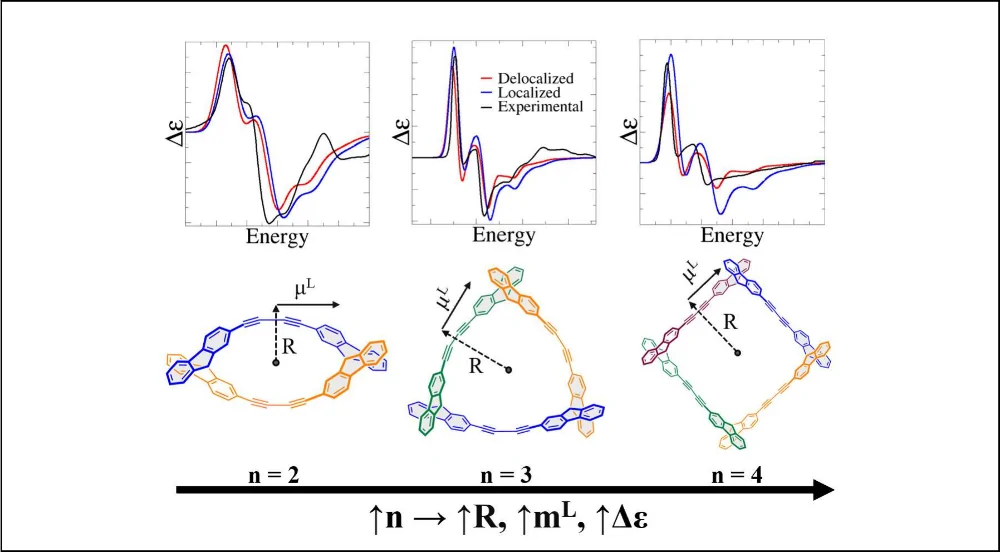

## 1 Introduction

In recent years, axially chiral scaffolds made up of repetitive units have attracted wide interest in fields such as organocatalysis, pharmaceutics, materials chemistry, quantum materials and molecular electronics displays.^1–5^ A major part of these applications exploits the rich optoelectronic properties of these molecules, which in many cases can be straightforwardly tuned by modifying the number of fragments building up the molecule.^6,7^ Regarding this, computational and theoretical methodologies serve as excellent tools,^8–10^ allowing for the rationalization of these trends, and previous works have shown to be successful in both providing satisfactory reproductions of experimental data and helping unveil the physical processes that act behind their properties.^11–13^

An interesting case of study is diethinylspirobifluorene (DES) macrocycles, which present promising applications owing to their stability and intense circular dichroism (ECD) responses.^14^ As evidenced in a previous work,^11^ the *D*_*n*_ symmetry of these species, with *n* being the number of fragments of the macrocycle, enables the presence of Jahn–Teller (JT) couplings, acting between degenerate pair of states and pseudo-Jahn–Teller (pJT) couplings,^15,16^ mixing the degenerate pair with close lying states whenever *n* > 2. They give rise to state mixings, conical intersections (CoIs), and symmetry breakings,^17^ thus indicating that nonadiabatic approaches are required to treat these systems properly. The linear vibronic coupling (LVC) model^18^ is particularly effective for these torsionally restrained molecules, making suitable an harmonic treatment of their vibrational modes.

The structure of DES macrocycles, made up of well-defined repetitive units, suggests that alternative approaches can be considered to reproduce and explain their chiroptical properties, describing the relevant coupled diabatic excited states either as delocalized excitations over the whole molecule or, in an exciton-like perspective, localized on each of the fragments. Once the vibronic model is properly set, spectra can be computed after simulating nonadiabatic quantum dynamics on the coupled states. The enantiomeric species (*P*_3_)-**DES**_3_, made up by three DES fragments, was indeed investigated according to these lines in a previous work,^11^ and both approaches were proven feasible through versatile diabatization protocols.^19,20^ Their combined usage showed the potential to obtain a more in-depth understanding of the properties of the system under investigation.^11^

On these grounds, in this work, we aim to apply exciton-like and delocalized vibronic Hamiltonians to the comparative investigation of a series of macrocycles formed by DES units, with *n* = 2, 3, and 4 being the number of fragments. More specifically, we will consider the enantiomers (*P*_2_)-**DES**_2_, (*P*_3_)-**DES**_3_, and (*P*_4_)-**DES**_4_, belonging to the *D*_*n*_ symmetry group (Figure 1). Performing accurate nonadiabatic quantum dynamical simulations on one side and exploiting symmetry and simple models to interpret their outcome, the scope of our investigation is 2-fold. On the one hand, we will investigate the relative impact of nonadiabatic interactions on the chiroptical properties of the macrocycle. On the other hand, we will determine how the number of fragments influences the shape and intensities of one-photon absorption (OPA, ϵ­(ω)) and electronic circular dichroism (ECD, Δϵ­(ω)) spectra and clarify if these differences are connected to the presence of JT and pJT effects. In particular, we will analyze how these trends are related to the simple number of units, to the mutual arrangements of the fragments, and to their intrinsic conformation in the macrocyle. The simple monomeric units are, in fact, expected to be planar and therefore achiral, but they can acquire an intrinsic chirality when bent in the macrocycle. At the same time, the pattern and extent of couplings and the relative orientation of the transition electric and magnetic dipoles of the monomeric units, which, in an excitonic picture, ultimately determine the overall chiroptical properties of the system, are expected to vary with the number of units in the macrocycle.

**Figure 1:**
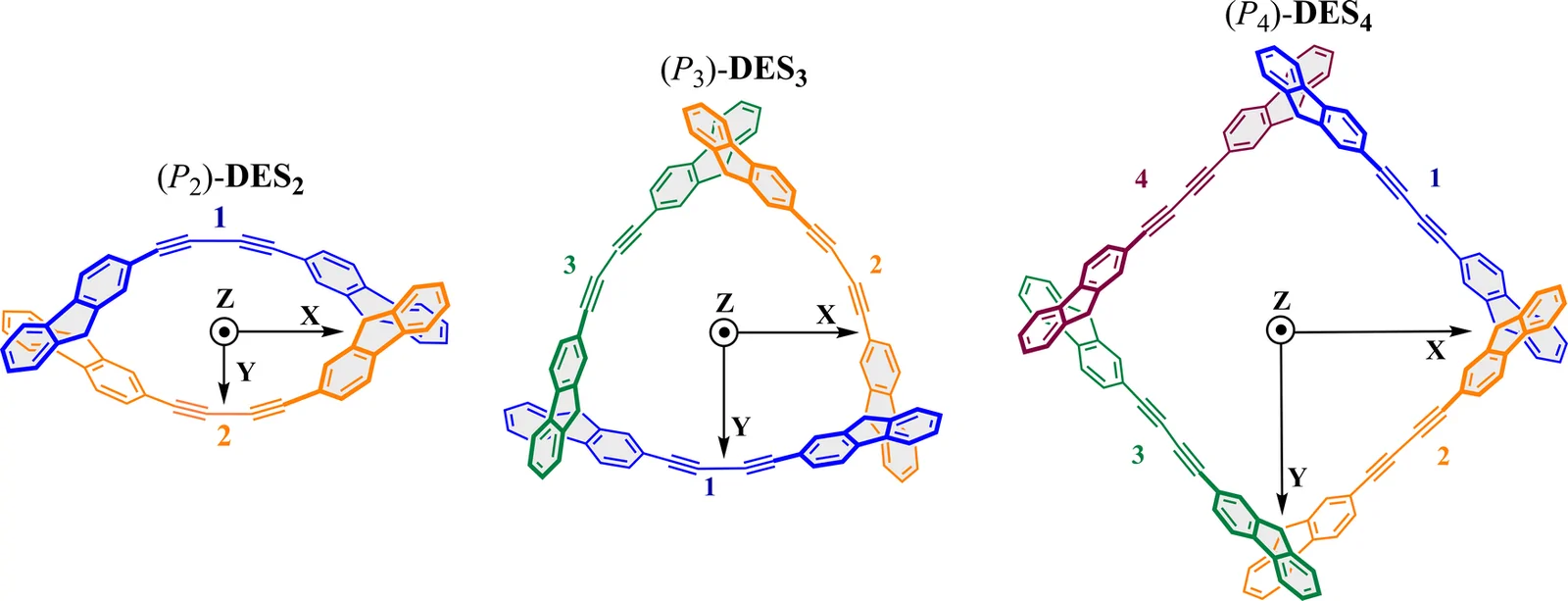
Schemes for the species studied in this work.

The manuscript is organized as follows. Section 2 introduces the LVC Hamiltonian, Section 3 provides the computational details, Section 4 is devoted to the results, and Section 5 reports the concluding remarks.

## 2 Theory

We adopt an LVC model which describes a set of diabatic states *d*_*i*_^γ^ and their couplings:

1 $$H=\underset{i}{\sum }\left(K+V_{ii}^{\gamma }\left(q\right)\right)\left|d_{i}^{\gamma }\right\rangle \left\langle d_{i}^{\gamma }\right|+\underset{i,j>i}{\sum }V_{ij}^{\gamma }\left(q^{\gamma }\right)\left(|d_{i}\rangle^{\gamma }\left\langle d_{j}{|}^{\gamma }+\right|d_{j}\rangle^{\gamma }\left\langle d_{i}^{\gamma }\right|\right)$$

where γ = *D*,*L* specify if diabatic states are defined in a delocalized (D) or localized (L) representation. The potential terms *V* ^γ^ depend on the dimensionless normal coordinates **q** ^γ^ and the kinetic energy term *K* ^γ^ on the conjugate momenta **p** ^γ^. *K* ^γ^ is a function of the diagonal matrix containing the vibrational frequencies of the ground state, **Ω** ^γ^:

2 $$K^{\gamma }=\frac{1}{2}{\left(p^{\gamma }\right)}^{T}\Omega^{\gamma }p^{\gamma }$$

and potential energy terms associated with dimensionless normal coordinates **q** ^γ^ are defined as

3 $$V_{ii}^{\gamma }\left(q^{\gamma }\right)=E_{ii}^{0,\gamma }+{\left(\lambda_{ii}^{\gamma }\right)}^{T}q^{\gamma }+\frac{1}{2}{\left(q^{\gamma }\right)}^{T}\Omega^{\gamma }q^{\gamma }$$

4 $$V_{ij}^{\gamma }\left(q^{\gamma }\right)=E_{ij}^{0,\gamma }+{\left(\lambda_{ij}^{\gamma }\right)}^{T}q^{\gamma }$$

where *E*_*ii*_^0,γ^ is the vertical energy for each *d*_*i*_^γ^, *E*_*ij*_^0,γ^ is the electronic coupling between the pair *d*_*i*_^γ^–*d*_*j*_^γ^, and **λ**_*ii*_^γ^ and **λ**_*ij*_^γ^ are the column vectors with gradient and interstate coupling respectively.

In a delocalized representation, diabatic states can be defined as identical to the adiabatic excited states of the macrocycle at the ground-state equilibrium geometry. By construction, *E*_*ij*_^0,*D*^ couplings are zero for these states which are delocalized over the monomers and belong to the *D*_2_, *D*_3_, and *D*_4_ symmetry groups for (*P*_2_)-**DES**_2_, (*P*_3_)-**DES**_3_, and (*P*_4_)-**DES**_4_, respectively. We call this model a standard LVC (St-LVC). As far as the vibrational coordinates are concerned, they are the delocalized normal coordinates computed for the ground electronic state of the macrocycle.

Exploiting the fact that the macrocyles are formed by well-differentiated fragments, an alternative model can be built considering excitations localized on each of the units as diabatic states. The parameters of this model can be obtained with a fragment diabatization LVC (FrD-LVC) protocol.^21^ Unlike the delocalized adiabatic states mentioned above, localized states are not eigenstates of the macrocycle, even at the reference geometry, and therefore *E*_*ij*_^0,*L*^ couplings are different from zero and can play a crucial role, especially in cases in which diabatic states are degenerate. In this work, for the FrD-LVC Hamiltonian, we adopt the vibrational coordinates of the monomeric units, computed from a normal-mode analysis of idealized monomers constrained in the arrangement imposed by the existence of the macrocycle. More details are given in the computational details.

### 2.1 Reference Idealized Models Provided by Simple Localized and Delocalized Hamiltonians

Exploiting the symmetric nature of these macrocycles, we can work out simplified idealized models that help understanding the expected relations between localized and delocalized pictures, thus facilitating the analysis of results. We start considering *n* states localized on each of the *n* monomers that build up the molecule (*m*), yielding 2 states for (*P*_2_)-**DES**_2_ (*m* = 2), 3 states for (*P*_3_)-**DES**_3_ (*m* = 3) and 4 states for (*P*_4_)-**DES**_4_ (*m* = 4). We can consider as well a single effective vibrational mode *q*_*n*(*m*)_ for each monomer, bridging the ground-state and excited-state equilibrium geometry. Each of these modes can be thought to be localized exclusively on one monomer, which ensures that they are independent of each other. These modes are assumed to be harmonic and by symmetry they are all equivalent, thus all *q*_*n*(*m*)_ share the same frequency ω_(*m*)_ and excited-state gradient λ_(*m*)_. Notice that this is a localized picture and, therefore, according to the notation introduced in the previous section, all the quantities here defined should bear a superscript “*L*”, which is skipped here for the sake of brevity.

Symmetry also dictates that all localized states share the same vertical energy *E*_0(*m*)_. Couplings between these localized states can be defined as excitonic. For (*P*_2_)-**DES**_2_, only one coupling term exists, β_(2)_, and for (*P*_3_)-**DES**_3_, all β_(3)_ are equal, since all pairs of localized states are equivalent by symmetry. For (*P*_4_)-**DES**_4_, we can define an excitonic term β_(4)_ coupling neighboring fragments, and a term η_(4)_ relating non-neighboring monomers. The localized potential energy Hamiltonian matrix can therefore be cast as^22^

5 $$H_{\mathrm{dia}\left(2\right)}^{\mathrm{vib},L}=A_{\left(2\right)}^{L}I_{2}+\left(\begin{array}{cc} E_{0\left(2\right)}+\lambda_{\left(2\right)}q_{1\left(2\right)} & \beta_{\left(2\right)}\\ \beta_{\left(2\right)} & E_{0\left(2\right)}+\lambda_{\left(2\right)}q_{2\left(2\right)} \end{array}\right)$$

6 $$H_{\mathrm{dia}\left(3\right)}^{\mathrm{vib},L}=A^{L}\left(3\right)I_{3}+\left(\begin{array}{ccc} E_{0\left(3\right)}+\lambda_{\left(3\right)}q_{1\left(3\right)} & \beta_{\left(3\right)} & \beta_{\left(3\right)}\\ \beta_{\left(3\right)} & E_{0\left(3\right)}+\lambda_{\left(3\right)}q_{2\left(3\right)} & \beta_{\left(3\right)}\\ \beta_{\left(3\right)} & \beta_{\left(3\right)} & E_{0\left(3\right)}+\lambda_{\left(3\right)}q_{3\left(3\right)} \end{array}\right)$$

7 $$H_{\mathrm{dia}\left(4\right)}^{\mathrm{vib},L}=A_{\left(4\right)}^{L}I_{4}+\left(\begin{array}{cccc} E_{0\left(4\right)}+\lambda_{\left(4\right)}q_{1\left(4\right)} & \beta_{\left(4\right)} & \eta_{\left(4\right)} & \beta_{\left(4\right)}\\ \beta_{\left(4\right)} & E_{0\left(4\right)}+\lambda_{\left(4\right)}q_{2\left(4\right)} & \beta_{\left(4\right)} & \eta_{\left(4\right)}\\ \eta_{\left(4\right)} & \beta_{\left(4\right)} & E_{0\left(4\right)}+\lambda_{\left(4\right)}q_{3\left(4\right)} & \beta_{\left(4\right)}\\ \beta_{\left(4\right)} & \eta_{\left(4\right)} & \beta_{\left(4\right)} & E_{0\left(4\right)}+\lambda_{\left(4\right)}q_{4\left(4\right)} \end{array}\right)$$

with **I**_*m*_ being the m-dimensional identity matrix, and $A_{\left(m\right)}^{L}=\frac{\omega }{2}\overset{m}{\underset{k=1}{\sum }}q_{k\left(m\right)}^{2}$ (we are expressing vibrational modes in dimensionless coordinates). At the ground-state equilibrium geometry, *q*_*n*(*m*)_ = 0, we can define the electronic Hamiltonian **H**_dia(*m*)_^vib,*L*^(**0**). The latter can be diagonalized by a simple symmetry transformation **T**_(*m*)_ (whose expression is given in eqs1–3 in the SI), yielding **H**_dia(*m*)_^vib,*D*^(**0**), with eigenvalues *E*_0(2)_ ± β_(2)_ for (*P*_2_)-**DES**_2_, *E*_0(3)_ + 2β_(3)_ and a degenerate pair *E*_0(3)_ – β_(3)_ for (*P*_3_)-**DES**_3_, and *E*_0(4)_ ± 2β_(4)_ + η_(4)_ and a degenerate pair *E*_0(4)_ – η_(4)_ for (*P*_4_)-**DES**_4_. The same symmetry transformation can be applied to the column matrix of the localized vibrational coordinates **q**_*n*(*m*)_, providing a set of delocalized coordinates **q**_*D*(*m*)_:

8 $$q_{D\left(m\right)}=T_{\left(m\right)}^{T}q_{n\left(m\right)}$$

where the subscript *D* indicates that the resulting coordinates are symmetry-adapted. Applying **T**_(*m*)_ both to the localized states and the localized coordinates we transform the vibronic potential energy matrix **H**_dia(*m*)_^vib,*L*^, which depends on fragment-localized states and coordinates, into **H**_dia(*m*)_^vib, *D*^, a function of delocalized states and coordinates:

9 $$H_{\mathrm{dia}\left(2\right)}^{\mathrm{vib},D}=T_{\left(2\right)}^{T}H_{\mathrm{dia}\left(2\right)}^{\mathrm{vib},L}T_{\left(2\right)}=A_{\left(2\right)}^{D}I_{2}+\left(\begin{array}{cc} E_{0\left(2\right)}+\beta_{\left(2\right)}+\frac{\lambda_{\left(2\right)}}{\sqrt{2}}q_{a\left(2\right)} & \frac{\lambda_{\left(2\right)}}{\sqrt{2}}q_{b\left(2\right)}\\ \frac{\lambda_{\left(2\right)}}{\sqrt{2}}q_{b\left(2\right)} & E_{0\left(2\right)}-\beta_{\left(2\right)}+\frac{\lambda_{\left(2\right)}}{\sqrt{2}}q_{a\left(2\right)} \end{array}\right)$$

10 $$H_{\mathrm{dia}\left(3\right)}^{\mathrm{vib},D}=A_{\left(3\right)}^{D}I_{3}+\left(\begin{array}{ccc} E_{0\left(3\right)}+2\beta_{\left(3\right)}+\frac{\lambda_{\left(3\right)}}{\sqrt{3}}q_{a\left(3\right)} & \frac{\lambda_{\left(3\right)}}{\sqrt{3}}q_{e_{a}\left(3\right)} & \frac{\lambda_{\left(3\right)}}{\sqrt{3}}q_{e_{b}\left(3\right)}\\ \frac{\lambda_{\left(3\right)}}{\sqrt{3}}q_{e_{a}\left(3\right)} & E_{0\left(3\right)}-\beta_{\left(3\right)}+\frac{\lambda_{\left(3\right)}}{\sqrt{3}}q_{a\left(3\right)}+\frac{\lambda_{\left(3\right)}}{\sqrt{6}}q_{e_{a}\left(3\right)} & -\frac{\lambda_{\left(3\right)}}{\sqrt{6}}q_{e_{b}\left(3\right)}\\ \frac{\lambda_{\left(3\right)}}{\sqrt{3}}q_{e_{b}\left(3\right)} & -\frac{\lambda_{\left(3\right)}}{\sqrt{6}}q_{e_{b}\left(3\right)} & E_{0\left(3\right)}-\beta_{\left(3\right)}+\frac{\lambda_{\left(3\right)}}{\sqrt{3}}q_{a\left(3\right)}-\frac{\lambda_{\left(3\right)}}{\sqrt{6}}q_{e_{a}\left(3\right)} \end{array}\right)$$

11 $$H_{\mathrm{dia}\left(4\right)}^{\mathrm{vib},D}=A_{\left(4\right)}^{D}I_{4}+\left(\begin{array}{cccc} E_{0\left(4\right)}+2\beta_{\left(4\right)}+\eta_{\left(4\right)}+\frac{\lambda_{\left(4\right)}}{2}q_{a\left(4\right)} & \frac{\lambda_{\left(4\right)}}{2}q_{e_{a}\left(4\right)} & \frac{\lambda_{\left(4\right)}}{2}q_{e_{b}\left(4\right)} & \frac{\lambda_{\left(4\right)}}{2}q_{b\left(4\right)}\\ \frac{\lambda_{\left(4\right)}}{2}q_{e_{a}\left(4\right)} & E_{0\left(4\right)}-\eta_{\left(4\right)}+\frac{\lambda_{\left(4\right)}}{2}q_{a\left(4\right)}+\frac{\lambda_{\left(4\right)}}{2}q_{b\left(4\right)} & 0 & \frac{\lambda_{\left(4\right)}}{2}q_{e_{a}\left(4\right)}\\ \frac{\lambda_{\left(4\right)}}{2}q_{e_{b}\left(4\right)} & 0 & E_{0\left(4\right)}-\eta_{\left(4\right)}+\frac{\lambda_{\left(4\right)}}{2}q_{a\left(4\right)}-\frac{\lambda_{\left(4\right)}}{2}q_{b\left(4\right)} & -\frac{\lambda_{\left(4\right)}}{2}q_{e_{b}\left(4\right)}\\ \frac{\lambda_{\left(4\right)}}{2}q_{b\left(4\right)} & \frac{\lambda_{\left(4\right)}}{2}q_{e_{a}\left(4\right)} & -\frac{\lambda_{\left(4\right)}}{2}q_{e_{b}\left(4\right)} & E_{0\left(4\right)}-2\beta_{\left(4\right)}+\eta_{\left(4\right)}+\frac{\lambda_{\left(4\right)}}{2}q_{a\left(4\right)} \end{array}\right)$$

where $A_{\left(m\right)}^{D}=\frac{\omega }{2}\overset{m}{\underset{k=1}{\sum }}q_{D\left(m\right)}^{2}$ .

The delocalized states in eqs 10 and 11 show that symmetry dictates the existence of degenerate pairs, which are split moving along the components of a degenerate pair of modes, creating Conical Intersections. It is extremely interesting here to notice that these simple transformations from a localized, exciton-type framework to a delocalized framework unveil an intrinsic fundamental connection between diagonal gradients and constant couplings of the localized picture and the coefficients of the linear couplings and the splittings at the reference geometry in the delocalized picture. In particular, all of the couplings in the delocalized model are predicted to be generated by the single gradient of the localized excitation, combined with signs and (simple) coefficients that are dictated by symmetry.

## 3 Computational Details

For all systems presented in this work, DFT/TD-DFT electronic structure data were computed in the gas phase at the CAM-B3LYP/6–31G­(d) of theory^23–25^ as implemented in Gaussian 16.^26^ St-LVC and FrD-LVC Hamiltonians were parametrized with Overdia 1.0.^27^ For the delocalized picture, the St-LVC Hamiltonian was obtained by defining diabatic states identical to some relevant number of the lowest-energy TD-DFT adiabatic states. The linear couplings λ_*ii*_(α) and λ_*ij*_(α) were obtained by displacing the ground state molecular structure along each normal mode α, computing the diabatic energies and couplings with a overlap-based diabatization protocol, and taking their numerical derivatives. Further details are given in refs. 20,21,28

Constant couplings in a FrD-LVC Hamiltonian can be obtained from the definition of *m* localized HOMO and LUMO orbitals, constructed from symmetry combinations of the *m* HOMO and LUMO with highest and lowest energy, respectively. Transitions can then be freely defined between these orbitals: if both HOMO and LUMO are localized over the same fragment, then the transition corresponds to a local excitation (*L*_*n*(*m*)_). The model can be easily extended to introduce the possible effect of charge transfer (*CT*) states, if the HOMO and the LUMO are localized over different fragments.

Table 1 shows the vertical energies and *E*_*ij*_^0,*L*^ values for the FrD-LVC localized states. These results allow us to exclude *CT* states from the Hamiltonian, given that they lie very high in energy with respect to *LE* states and *E*_*ij*_^0,*L*^ terms are too low. Consequently, *CT* states will not be considered further. As discussed in ref 11, a description solely based on localized HOMO–LUMO transitions for the local excitations leads to notable differences between computed and experimental spectra for (*P*_3_)-**DES**_3_ due to the presence of non-negligible additional orbital contributions. Thus, only the couplings *E*_*ij*_^0,*L*^ terms were extracted from the approximate HOMO–LUMO Hamiltonian, whereas Vertical Energies and transition dipoles were extracted from the S_1_ state of a single fragment (data in Table S8 of the SI), in which free bonds on the bridging atoms have been saturated with hydrogen atoms and the equilibrium geometry was calculated by freezing al torsional degrees of freedom. Notice that in Table 1 the couplings *E*_*ij*_^0,*L*^ between local adjacent and nonadjacent excitations were named β and η, i.e. the same nomenclature we used in the idealized model in Section 2.1.

**Table 1:** Vertical Energies (VE), Interstate Couplings between Adjacent Localized States (β), and Largest Interstate Couplings for *CT* States (ι) for the Localized Hamiltonian of the Species Investigated in This Work^a^

(*P*_2_)-**DES**_2_				
state	VE (eV)	β (eV)	η (eV)	ι (eV)
*L*_*a*_	4.20	–0.093		
*CT*_*a*→*b*_	4.84			–0.060
(*P*_3_)-**DES**_3_				
*L*_*a*_	4.00	–0.059		
*CT*_*a*→*b*_	4.94			–0.035
(*P*_4_)-**DES**_4_				
*L*_*a*_	3.94	–0.060	–0.024	
*CT*_*a*→*b*_	4.94			–0.041
*CT*_*a*→*c*_	5.31			–0.014

a (η) denotes interstate couplings between nonadjacent localized states, present only in (*P*_4_)-**DES**_4_. Subscripts *a*,*b* and *a*,*c* denote adjacent and nonadjacent fragments, respectively. CAM-B3LYP/6-31G­(d) level of theory.

Different approaches were tested and analyzed to compute magnetic transition dipole moments (MTDMs) and are presented in the Supporting Information; however, the discussion in this main text will consider the results in which the MTDMs were defined as those of the saturated monomers displaced at their respective positions in the macrocycle. For a transition belonging to monomer *i*, these MTDMs can can be computed as^29^

12 $$m_{i}^{L}=m_{i\left(int\right)}^{L}+\frac{ie\hbar }{2m_{e}c}R_{i}\times \mu_{i}^{vel}=m_{i\left(int\right)}^{L}+m_{i\left(ext\right)}^{L}$$

where ***m***_*i*(*int*)_^*L*^ is the MTDM of the transition when the monomer is placed at the center of mass of the macrocycle, **R**_*i*_ is the distance of the monomer with respect to the origin of coordinates, and **μ**_*i*_^*vel*^ is the electric transition dipole moment of the transition in the velocity gauge. This expression can be conveniently described as a sum between intrinsic (***m***_*i*(*int*)_^*L*^) and extrinsic (***m***_*i*(*ext*)_^*L*^) contributions, with the latter arising due to the monomer not being placed at the center of coordinates.

OPA and ECD spectra were calculated in a time-dependent framework according to the following expressions:^28,30,31^

13 $$\epsilon \left(\omega \right)=\frac{\omega N_{A}}{6000\times \ln10\times \hbar c_{0}\epsilon_{0}}\underset{ij}{\sum }\left(\mu_{i}^{\gamma }\cdot \mu_{j}^{\gamma }\right)c_{ij}^{\gamma }\left(\omega \right)$$

14 $$\Delta \epsilon \left(\omega \right)=\frac{\omega N_{A}}{1500\times \ln10\times \hbar c_{0}^{2}\epsilon_{0}}\underset{ij}{\sum }\left(m_{i}^{\gamma }\cdot \mu_{j}^{\gamma }\right)c_{ij}^{\gamma }\left(\omega \right)$$

where γ = *L*,*D* specifies the localized or delocalized representation, ω is the transition frequency, **μ**_*i*_^γ^ is the ETDM between the ground state and excited state *i*, ***m***_*j*_^γ^ is the imaginary part of the MTDM of said transition. The computation of the ECD in the delocalized picture is origin invariant within the well-known limits for pure electronic computations.^32^ As far as the localized picture is concerned, a proof of its origin invariance is given in Section 1.2 of the Supporting Information. The spectral components *c*_*ij*_^γ^(ω) are obtained by the Fourier transform of the auto- (*i* = *j*) and cross- (*i* ≠ *j*) time correlation functions in the brackets:

15 $$c_{ij}^{\gamma }\left(\omega \right)=\int_{-\infty }^{\infty }dte^{i\omega t-\Gamma t^{2}}\left\langle 0;d_{i}^{\gamma }\right|e^{-iHt/\hbar }\left|d_{j}^{\gamma };0\right\rangle$$

where Γ is a damping factor which corresponds to a Gaussian broadening in the frequency domain. Due to this choice, it is more suited to describe static disorder (inhomogeneous broadening) which is usually the dominant broadening effect in solution. Here Γ is fixed to a value that approximately fits the width of the experimental vibronic bands, thus phenomenologically accounting also for homogenoeus brodening. The correlation functions are computed numerically performing vibronic wavepacket propagations on all diabatic states included in the LVC Hamiltonians. To that end we employed the multiconfigurational time-dependent Hartree (ML-MCTDH) method,^33–35^ as implemented in the Quantics code.^36^

Despite the efficiency of ML-MCTDH, these computations are challenged by the increasing number of normal coordinates of the investigated macrocycles, respectively 252 for (*P*_2_)-**DES**_2_, 381 for (*P*_3_)-**DES**_3_ and 510 for (*P*_4_)-**DES**_4_. Therefore, for the St-LVC Hamiltonian, we followed two different routes, either (i) neglecting coordinates with very small couplings (less than 0.01 eV), obtaining reduced models with respectively 42 (*P*_2_)-**DES**_2_, 47 (*P*_3_)-**DES**_3_ and 51 (*P*_4_)-**DES**_4_ vibrational degrees of freedom, or (ii) adopting a hierarchical transformation of the LVC Hamiltonians in blocks of effective coordinates. The latter coordinates are defined so that a few blocks are enough to describe accurately the short time evolution of the system.^37–41^ Each subsequent block can be defined recursively with respect to the previous one, counting each with *n*(*n* + 1)/2 effective modes, where *n* is the number of electronic states, therefore, 3, 6, and 10 for (*P*_2_)-**DES**_2_, (*P*_3_)-**DES**_3_ and (*P*_4_)-**DES**_4_, respectively. In Section 7 of the Supporting Information we cross-check that both (i) and (ii) approaches converge to the same spectra. For the localized approach, only the hierarchical Hamiltonian was employed to compute spectra; in particular, for each monomer we included up to 4 effective modes, therefore, a total of 8, 12, and 16 coordinates for (*P*_2_)-**DES**_2_, (*P*_3_)-**DES**_3_ and (*P*_4_)-**DES**_4_, corresponding to 4 blocks of the hierarchy. For both delocalized and localized methodologies, we checked in the Supporting Information that 4 blocks were enough to ensure convergence of the spectra (Figures S30–S32).

## 4 Results

### 4.1 Main Characteristics of the Potential Energy Surfaces Suggested by the Idealized Reference Model

Before discussing the TD-DFT results, it is worth analyzing the main features of the PES of the systems as suggested by the simplified idealized models presented in section 2.1. Figure 2 reports the adiabatic PES for the relevant states for each of the macrocycles presented in this work, obtained through the diagonalization of eqs 9–11. These parameters are chosen considering the two most relevant modes regarding the coupling between states; namely, *q*_*a*(2)_ and *q*_*b*(2)_ for (*P*_2_)-**DES**_2_, and the pairs *q*_*e*_*a*_(3)_/*q*_*e*_*b*_(3)_ and *q*_*e*_*a*_(4)_/*q*_*e*_*b*_(4)_ for (*P*_3_)-**DES**_3_ and (*P*_4_)-**DES**_4_, respectively, and will be discussed in the following pages.

**Figure 2:**
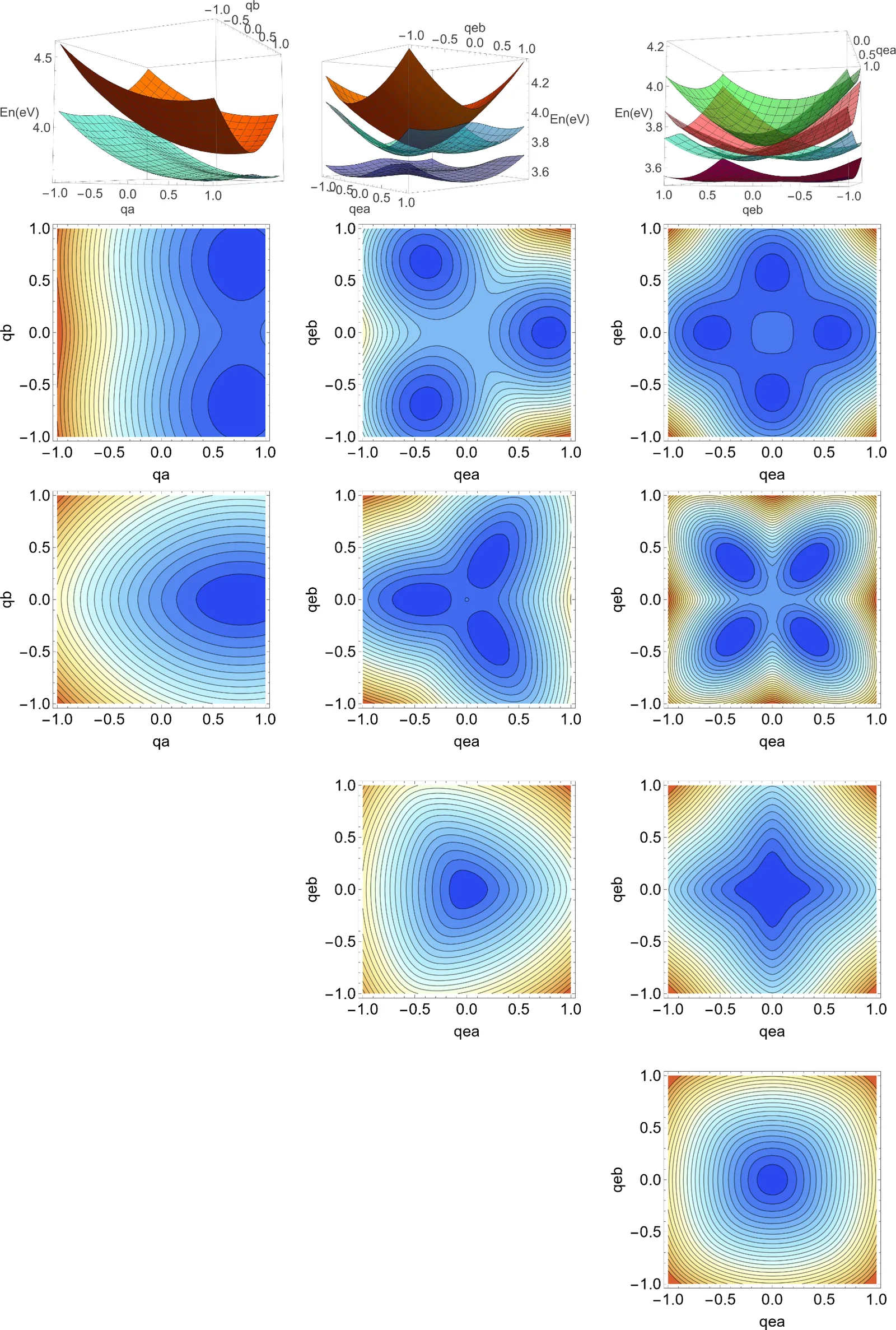
Adiabatic PES for the relevant excited states of species studied in this work: first row, left to right, 3D adiabatic PES plots for (*P*_2_)-**DES**_2_, (*P*_3_)-**DES**_3_ and (*P*_4_)-**DES**_4_. The following rows show individual contour plots for states S_1_ to S_2_/S_3_/S_4_, respectively. Surfaces are a function of the two relevant modes (*q*_*a*(2)_ and *q*_*b*(2)_ for (*P*_2_)-**DES**_2_, and the degenerate pair *q*_*e*_*a*_(3)_ and *q*_*e*_*b*_(3)_ for (*P*_3_)-**DES**_3_ and (*P*_4_)-**DES**_4_ (*q*_*e*_*a*_(4)_ and *q*_*e*_*b*_(4)_). Frequency of all normal modes was set to 0.3 eV (typical of a C-C triple bond stretching) and λ_(*n*)_ to 0.32 eV for all surfaces, β and η couplings were set to those reported in Table 1, and *E*_0(*n*)_ to 3.83 eV for (*P*_2_)-**DES**_2_, 3.69 eV for (*P*_3_)-**DES**_3_ and 3.68 eV for (*P*_4_)-**DES**_4_.

For (*P*_2_)-**DES**_2_, the lower-energy PES exhibits two minima reminiscent of the local excitations. The two PESs, however, show no CoI due to the lack of degenerate states (see section 3.1.1 of the Supporting Information). Differently, due to their higher symmetry, both (*P*_3_)-**DES**_3_ and (*P*_4_)-**DES**_4_ show a CoI between the second and third states (S_2_ and S_3_), which are degenerate at the FC point, whereas the degeneracy is lifted along the two modes. Interestingly, in analogy with the two minima of the S_1_ of (*P*_2_)-**DES**_2_, the S_1_ PESs of (*P*_3_)-**DES**_3_ and (*P*_4_)-**DES**_4_ exhibit, respectively, 3 and 4 wells reminiscent of the localized minima.

The S_2_ PES, i.e., the lower sheet of the conical intersection, also shows 3 and 4 minima for (*P*_3_)-**DES**_3_ and (*P*_4_)-**DES**_4_, but their direction is rotated in the plane of the degenerate modes. S_3_ and S_4_ PESs have a symmetric minimum at the FC point. The S_3_ minimum is actually a cusp (the upper sheet of the CoIn) with a shape that has a 3-fold or 4-fold symmetry for (*P*_3_)-**DES**_3_ and (*P*_4_)-**DES**_4_. Finally, the shape of the S_4_ minimum (*P*_4_)-**DES**_4_ has a perfect circular symmetry.

(*P*_3_)-**DES**_3_ exhibits both JT and pJT (eq 10) effects, which are expected to impact the chiroptical properties as also demonstrated in a previous work.^11^ According to this idealized model, (*P*_4_)-**DES**_4_ only presents pJT couplings (eq 11), a property that arises from the symmetry of the system. pJT couplings were shown to dominate over JT ones in (*P*_3_)-**DES**_3_.^11^ The pJT effect in (*P*_4_)-**DES**_4_ is expected to have a substantially higher impact than S_1_/S_4_ coupling because of the comparatively higher energy gap between S_1_ and S_4_. Adiabatic surfaces obtained by diagonalizing the Hamiltonian after setting all interstate couplings except pJT ones to zero are identical (), showing a minimum around the FC point whereas S_2_ and S_3_ become completely degenerate along the pair *q*_*e*_*a*_(4)_/*q*_*e*_*a*_(4)_.

Notice that JT and pJT nonadiabatic effects are not strictly present in (*P*_2_)-**DES**_2_ due to the absence of a degeneracy dictated by symmetry (eq 9). However, on the one hand, the small gap between S_1_ and S_2_ and, on the other hand, the fact that this idealized model predicts that their coupling has, in an excitonic picture, the same origin of those responsible for JT and pJT in the higher-symmetry (*P*_3_)-**DES**_3_ and (*P*_4_)-**DES**_4_, lead to expect that nonadiabatic interactions will impact also the chiroptical properties of (*P*_2_)-**DES**_2_ species.

After this general survey of the main characteristics expected for the states of (*P*_2_)-**DES**_2_, (*P*_3_)-**DES**_3_, and (*P*_4_)-**DES**_4_, in the following a separated discussion for each of the molecules will be presented, comparing experimental data with the predictions of both delocalized and localized approaches, and analyzing how the computed parameters compare to the predictions of the idealized Hamiltonian matrices discussed here.

### 4.2 (*P*_2_)-**DES**_2_

#### 4.2.1 LVC Parameters

Table 2 reports the data for the lowest-energy excited states for (*P*_2_)-**DES**_2_ in the ground state minimum. Our St-LVC approach considers only the two lowest states, since S_3_ to S_6_ exhibit comparatively low dipole and rotatory strengths. S_1_ has a very small oscillator strength and a high positive rotatory strength (≈1700 × 10^–40^ cgs) and S_2_ is bright and has a strong negative rotatory strength (≈−2000 × 10^–40^ cgs). Table 3 summarizes, out of the 42 modes with linear couplings >0.01 eV, those with the highest inter and intrastate couplings. Two sets of quasi-degenerate groups (214–216–219 and 223–224) account for more than 90% of couplings out of all the modes. The modes in the latter group (≈2393 cm^–1^) are combinations of the symmetric triple-bond stretchings on each branch of the molecule. The modes in the former group (≈1700 cm^–1^) consist of double-bond CC stretchings mostly localized on the spirofluorene fragments.

**Table 2:** Vertical Energies (VE), Dipole Strength, Rotatory Strength in Velocity Gauge (*R*_*vel*_) and Angle between Electric and Magnetic Transition Dipole Moments (θ_*EM*_) for the Eight Lowest Energy Adiabatic States of (*P*_2_)-**DES**_2_-**2** ^a^

system	state	symm.	VE (eV)	Dip. Str (a.u.)	*R*_*vel*_ (10^–40^ cgs)	θ_*EM*_
(*P*_2_)-**DES**_2_	S_1_	*B*_2_	3.75	1.23	1727.70	0.00
	S_2_	B_1_	3.90	17.69	–1969.86	180.00
	S_3_	B_3_	4.01	0.13	–10.43	180.00
	S_4_	A	4.01	0.00	0.00	90.00
	S_5_	B_3_	4.13	2.46	354.12	0.00
	S_6_	A	4.15	0.00	0.00	90.00
	S_7_	B_2_	4.39	0.39	747.52	0.00
	S_8_	B_1_	4.46	11.62	–1603.82	180.00

a CAM-B3LYP/6-31G­(d) level of theory.

**Table 3:** LVC Parameters for the Most Relevant Normal Modes of (*P*_2_)-**DES**_2_ ^a^

(*P*_2_)-**DES**_2_-**2** (*D*_2_)					
modes	symm.	freq (cm^–1^)	λ_11_	λ_22_	λ_12_
214	B_3_	1698.78	0.00	0.00	–0.10
216	A	1700.75	0.08	0.06	0.00
219	A	1705.50	–0.08	–0.07	0.00
223	B_3_	2392.70	0.00	0.00	–0.23
224	A	2392.83	–0.22	–0.24	0.00
norm 5 modes			0.250	0.262	0.249
norm all modes			0.272	0.277	0.267

a All λ in eV. CAM-B3LYP/6-31G­(d) level of theory.

These parameters can be analyzed on the grounds of what is predicted by the idealized model for the delocalized Hamiltonian matrix described in eq 9. The energy gap between S_1_ and S_2_ is 0.15 eV, close to the prediction using the idealized model, considering the computed electronic coupling between localized states (Table 1), β_(2)_ ≈−0.09 eV and the fact that the gap is 2β. Couplings with additional states, not included in the idealized model, can explain the smaller energy gap computed by TD-DFT. For instance, it is possible that the interaction of S_2_ with the closest electronic state of the same *B*_1_ symmetry (S_8_) is lowering its energy.

As far as the vibrational contributions are concerned, the idealized model also predicts that modes of (*P*_2_)-**DES**_2_ should appear in pairs of (quasi-) degenerate normal modes: a totally symmetric one accounting for the gradients in both states (*q*_*a*(2)_) and an antisymmetric one (*q*_*b*(2)_) responsible for a coupling between the two states equal in magnitude to the gradient along *q*_*a*(2)_. Modes 223 and 224 perfectly match this prediction. The fact that at ∼1700 cm^–1^ we found as well 3 important quasi-degenerate modes should be ascribed to mixings due to couplings not present in the idealized model (the manifold of vibrational modes in the region at 1700 cm^–1^ is much more dense than at 2400 cm^–1^). Notwithstanding some deviations from the idealized model, it is worth noticing that the module of the gradient along all modes for each diabatic state and of the vector of the interstate couplings is very similar, with the norm for all modes being 0.272 (S_1_), 0.277 (S_2_) and 0.267 (coupling S_1_/S_2_), respectively.

#### 4.2.2 OPA and ECD Spectra

Figure 3 compares experimental OPA and ECD spectra for (*P*_2_)-**DES**_2_ with those computed within both localized and delocalized pictures. Remarkably, both reproduce correctly all main spectral features; namely, for OPA, a peak at ≈3.4 eV followed by the maximum peak at ≈3.6 eV, capturing nicely the relative intensities; for ECD, a positive peak at ≈3.3 eV, red-shifted with respect to the first OPA peak, with a weak positive shoulder at ≈3.5 eV, and a negative peak at around 3.6 eV. Relative intensities for ECD are nicely captured as well.

**Figure 3:**
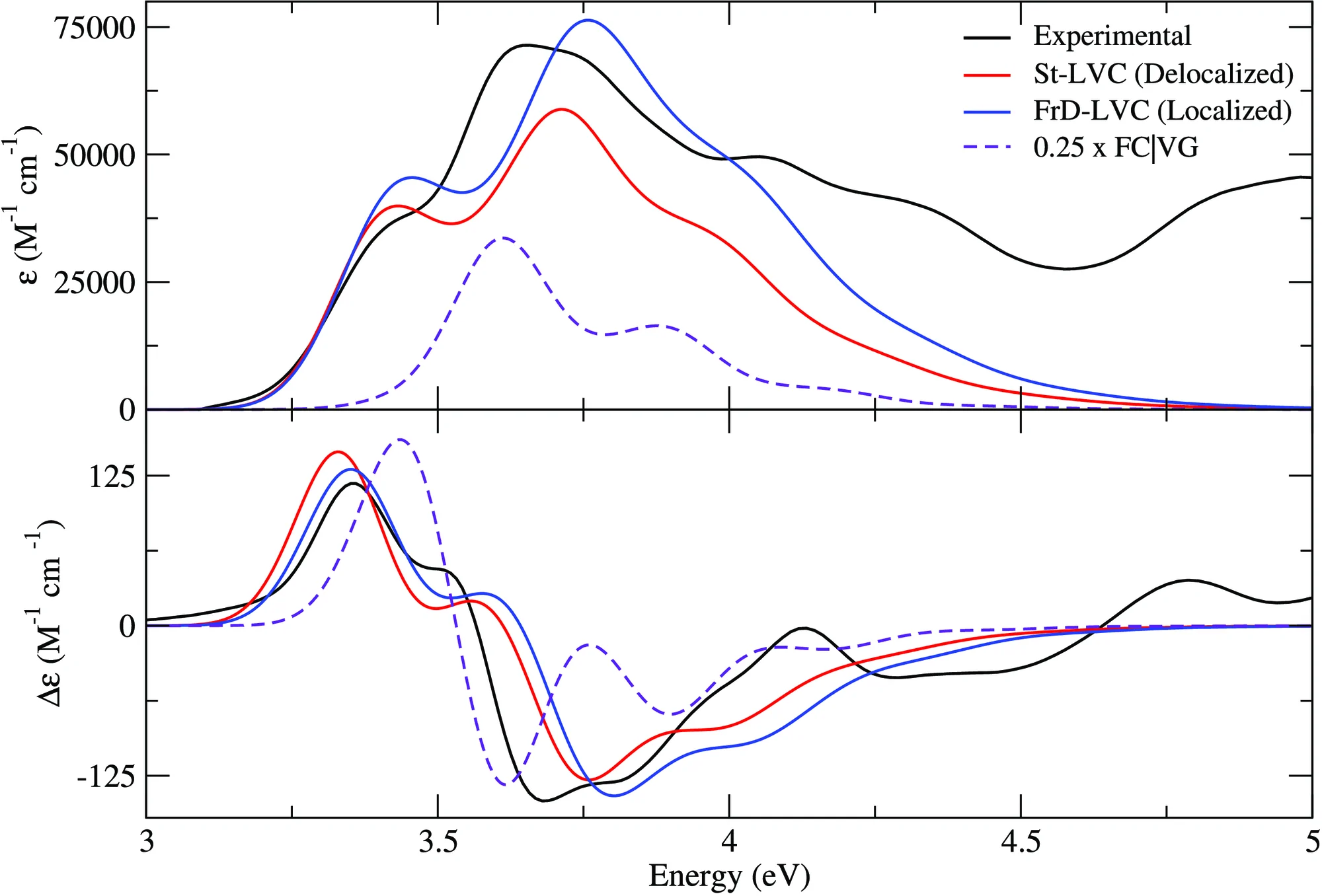
Comparison of computed and experimental ABS and ECD spectra for (*P*_2_)-**DES**_2_. Computed spectra were red-shifted by 0.150 eV, and a Gaussian broadening with HWHM = 0.09 eV was applied for both ABS and ECD. Computed LVC OPA and ECD spectra were scaled by 0.75 and 0.5, respectively. CAM-B3LYP/6–31G­(d) level of theory.

The remarkable impact of nonadiabatic couplings on the spectra can be emphasized setting linear couplings between S_1_ and S_2_ to zero in the St-LVC, which in practice converts the nonadiabatic LVC model into the adiabatic Franck–Condon Vertical Gradient (FC|VG) model.^42^ The agreement of resulting spectra with experimental data is much poorer. Specifically, in the FC|VG OPA spectrum, only a shoulder is predicted on the blue side of the main peak at ≈3.70 eV, whereas St-LVC correctly predicts a peak appearing at a lower energy as well. According to the FC|VG model, the contribution of S_1_ in the OPA spectrum is negligible, due to its very low dipole strength, so that practically the spectrum is dominated by S_2_. This finding clarifies that the shoulder at lower energy arises due to a contribution of S_1_ activated by nonadiabatic couplings. The ECD FC|VG spectrum disagrees most notably for the lack of a second positive peak at ≈3.50 eV, similarly indicating that it arises from the interaction of S_1_ and S_2_ contributions mediated by nonadiabatic couplings.

### 4.3 (*P*_3_)-**DES**_3_

#### 4.3.1 LVC Parameters

The chiroptical properties of (*P*_3_)-**DES**_3_ have been already investigated in ref 11. In this work they have been reproduced, and the convergence has been further checked with respect to computations without using the hierarchical representation of the Hamiltonian adopted in ref 11. Results are equivalent and therefore they are just concisely summarized here in order to facilitate a comparison with (*P*_2_)-**DES**_2_ and (*P*_4_)-**DES**_4_, whereas a more extended discussion is given in Section 4 of the Supporting Information.

Table 4 shows the data for the 3 lowest-lying in energy excited states for (*P*_3_)-**DES**_3_. The first excited state, S_1_, is weakly absorbing and counts with a high rotatory strength (≈3858 × 10^–40^ cgs). The rotatory strength of the S_2_/S_3_ degenerate pair is about half of this value, whereas their dipole strengths are very high at 25.10 a.u.; therefore, these states are expected to play a prominent role in the low-energy region for both OPA and ECD spectra. Other states lie higher in energy and are not expected to contribute remarkably to the spectra (see section 4 in the SI).

**Table 4:** Vertical Energies (VE), Dipole Strength (Dip. Str. in a.u.), Rotatory Strength (in Velocity Gauge (*R*_*vel*_) and the Angle between Electric and Magnetic Transition Dipole Moments (θ_*EM*_ for the Six Lowest Energy Adiabatic States of (*P*_3_)-**DES**_3_ ^a^

system	state	symm.	VE (eV)	Dip. Str (a.u.)	*R*_*vel*_ (10^–40^ cgs)	θ_*EM*_
(*P*_3_)-**DES**_3_	S_1_	A_2_	3.58	2.10	3858.02	0.00
	S_2_	E	3.74	25.10	–2045.47	180.00
	S_3_	E	3.74	25.10	–2045.47	180.00

a CAM-B3LYP/6-31G­(d) level of theory.

Table S10 in SI shows that 3 modes lying at ∼2400 cm^–1^ that correspond to a combination of triple-bond stretchings account for ≈80% of the total vibronic coupling. The values of their St-LVC couplings perfectly confirm the predictions of the idealized model presented in Section 2.1.

Figure 4 shows that, also for (*P*_3_)-**DES**_3_, St-LVC and FrD-LVC, nonadiabatic approaches provide a very satisfactory simulation of both OPA and ECD spectra. Neglecting nonadiabatic interactions (FC|VG computations) leads to an inversion of the intensities of the two main peaks of the OPA and, in ECD, to the loss of the two weak shoulders, negative and positive, at ∼3.4–3.5 eV, respectively, that characterize the experimental spectrum and are very nicely reproduced by St-LVC and FrD-LVC models.

**Figure 4:**
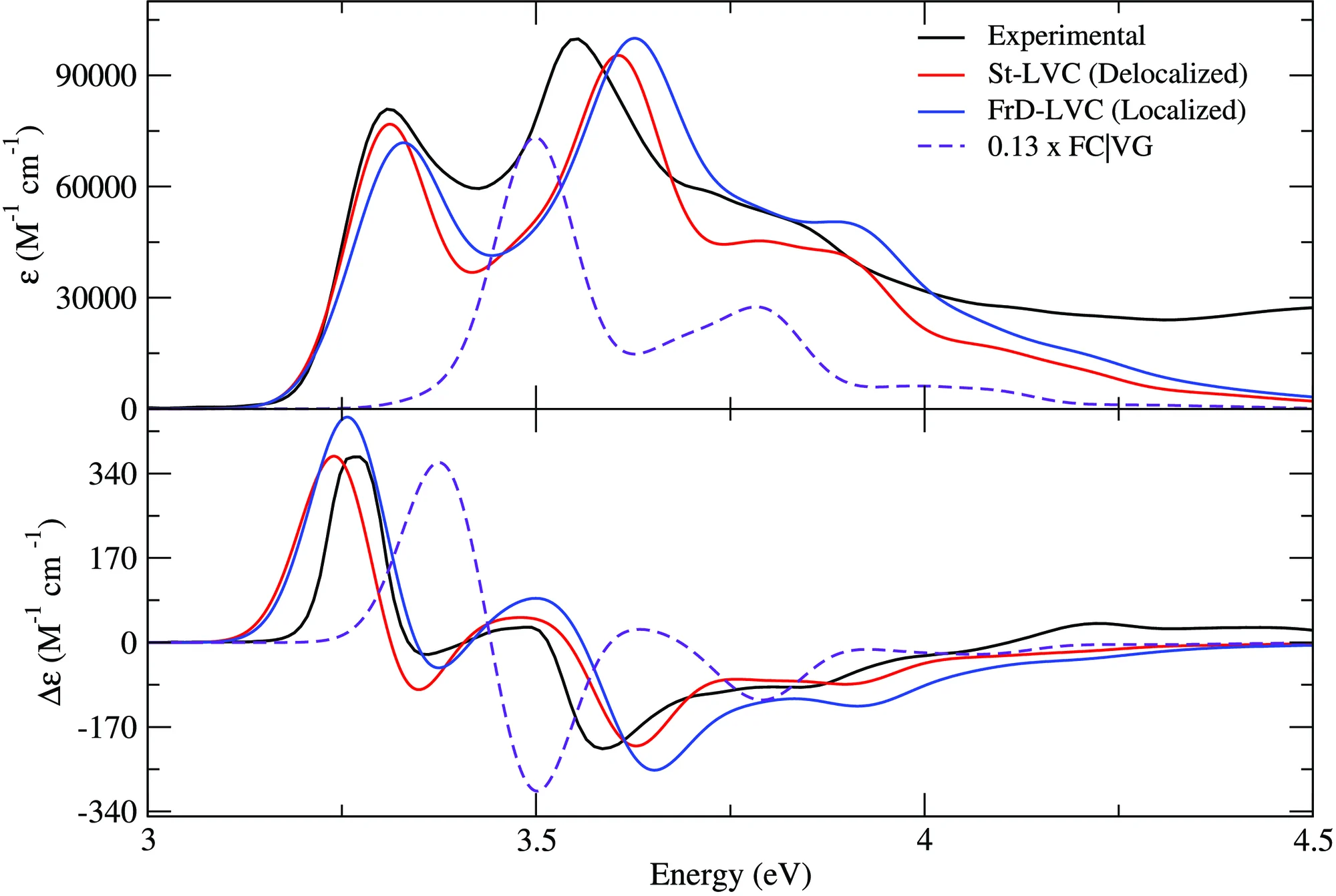
Comparison of computed and experimental ABS and ECD spectra for (*P*_3_)-**DES**_3_. Computed spectra were red-shifted by 0.105 eV, and a Gaussian broadening with HWHM = 0.064 eV was set for both ABS and ECD. LVC OPA and ECD spectra were scaled by 0.35 and 0.26, respectively. CAM-B3LYP/6–31G­(d) level of theory.

#### 4.3.2 OPA and ECD Spectra

### 4.4 (*P*_4_)-**DES**_4_

#### 4.4.1 LVC Parameters

An LVC Hamiltonian was parametrized for (*P*_4_)-**DES**_4_ considering the 4 lowest-energy TD-DFT excited states. Higher energy states (Table 5) do not play a prominent role, given that they are dark and the energy gap between S_4_ and S_5_ is too high for linear couplings to operate. Like in smaller macrocycles, S_1_ exhibits a weak dipole strength and a very high rotatory strength (≈6700 × 10^–40^ cgs). The degenerate pair S_2_ and S_3_ displays very high dipole strengths and rotatory strengths. S_4_ has vanishing dipole and rotatory strengths; however, it has been included in the model, since the idealized model in Section 2.1 suggests that it originates from the same mixing of local excitations forming S_1_–S_3_, and moreover because it is close in energy to S_2_/S_3_.

**Table 5:** Vertical Energies (VE), Dipole Strength (Dip. Str. in a.u.), Rotatory Strength (in Velocity Gauge (*R*_*vel*_) and Angle between Electric and Magnetic Transition Dipole Moments (θ_*EM*_ for the Six Lowest Energy Adiabatic States of (*P*_4_)-**DES**_4_ ^a^

system	state	symm.	VE (eV)	Dip. Str (a.u.)	*R*_*vel*_ (10^–40^ cgs)	θ_*EM*_
(*P*_4_)-**DES**_4_	S_1_	A_2_	3.54	2.90	6739.28	0.00
	S_2_	E	3.66	38.60	–3097.82	180.00
	S_3_	E	3.66	38.60	–3097.82	180.00
	S_4_	B_1_	3.71	0.00	0.00	90.00
	S_5_	E	4.14	0.00	–0.55	180.00
	S_6_	E	4.14	0.00	–0.32	180.00
	S_7_	A_1_	4.14	0.00	0.00	
	S_8_	E	4.36	0.00	–2.61	180.00
	S_9_	E	4.36	0.00	–2.54	180.00
	S_10_	A_1_	4.36	0.00	0.00	

a CAM-B3LYP/6-31G­(d) level of theory.

The existence of a degenerate pair laying in between the first and fourth states fully confirms the predictions of the ideal delocalized model (eq 11). According to TD-DFT, the degenerate pair is 0.12 eV above S_1_ and 0.05 eV below S_4_. The idealized model is in reasonable agreement predicting the same gaps as −2β – 2η = 0.17 and −2β + 2η = 0.07 eV. The comparatively higher mismatch between the energy gap with respect to S_1_ compared to that for S_4_ might be ascribed to interactions with other states not included in the idealized model. For instance, there are other two degenerate pairs, namely S_5_/S_6_ and S_8_/S_9_. Sharing the same symmetry of S_2_/S_3_, they might signal the existence of interactions which have lowered S_2_/S_3_ energy.

The normal modes with the largest displacements and couplings, listed in Table 6, share a clear similarity with those individuated for (*P*_2_)-**DES**_2_. More specifically, they are combinations of CC triple bond stretchings and, in agreement with what is predicted by the model in Section 2.1, they form a quadruplet of quasi-degenerate modes (451–454) at 2400 cm^–1^ which accounts for ≈ 80% of the total gradients/couplings. They are a totally symmetric mode (*q*_*a*(4)_) with positive gradients in all 4 states (mode 454), a pair of *E* symmetry modes (*q*_*e*_*a*_(4)_ and *q*_*e*_*b*_(4)_) which couple the nondegenerate states with the degenerate pair (modes 452 and 453), being one of the couplings of opposite sign with respect to the other 3, and a nontotally symmetric mode (*q*_*b*(4)_) which couples the nondegenerate pair. In agreement with the predictions of the model in Section 2.1, the norm for all of these couplings has exactly the same value (0.16 eV). Moreover, the data perfectly confirm the lack of a direct linear coupling of the degenerate pair, i.e., the absence of JT couplings. As in smaller macrocycles, we observe also moderate gradients and couplings along other modes, in particular, ∼1700 cm^–1^, arising from combinations of double-bond stretchings (check Table S11 in the Supporting Information).

**Table 6:** LVC Parameters for the Most Relevant Normal Modes of (*P*_4_)-**DES**_4_ ^a^

modes	symm.	freq (cm^–1^)	λ_11_	λ_22_	λ_33_	λ_44_	λ_12_	λ_13_	λ_14_	λ_23_	λ_24_	λ_34_
451	b_2_	2400.24	0.00	–0.16	0.16	0.00	0.00	0.00	0.16	0.00	0.00	0.00
452	e	2400.26	0.00	0.00	0.00	0.00	–0.16	0.00	0.00	0.00	0.16	0.00
453	e	2400.26	0.00	0.00	0.00	0.00	0.00	–0.16	0.00	0.00	0.00	–0.16
454	a_1_	2400.29	0.16	0.16	0.16	0.16	0.00	0.00	0.00	0.00	0.00	0.00
norm 4 modes			0.165	0.227	0.227	0.158	0.162	0.162	0.159	0.000	0.159	0.159
norm all modes			0.199	0.270	0.270	0.187	0.194	0.194	0.191	0.007	0.188	0.188

a All *λ* in eV. CAM-B3LYP/6-31G­(d) level of theory.

#### 4.4.2 OPA and ECD Spectra

Figure 5 shows that, in a similar fashion as for (*P*_2_)-**DES**_2_ and (*P*_3_)-**DES**_3_, also for (*P*_4_)-**DES**_4_ the OPA and ECD spectra computed with both delocalized and localized approaches are in very good agreement with experimental data. Both computational approaches correctly predict the peaks at ≈3.20 and 3.50 eV with correct relative intensities. Also, for this macrocycle, neglecting nonadiabatic couplings leads to a clear worsening of the agreement, as shown by FC|VG spectra, which are unable to reproduce the position and relative intensities of the peaks. Additionally, the shoulders between these peaks in ECD are not reproduced without nonadiabatic couplings. It is worthy of notice that for (*P*_4_)-**DES**_4_ the difference between FC|VG and St-LVC approaches mainly arises from the lack of pJT couplings, showing that they play a crucial role, whereas the direct S_1_/S_4_ coupling has a negligible effect.

**Figure 5:**
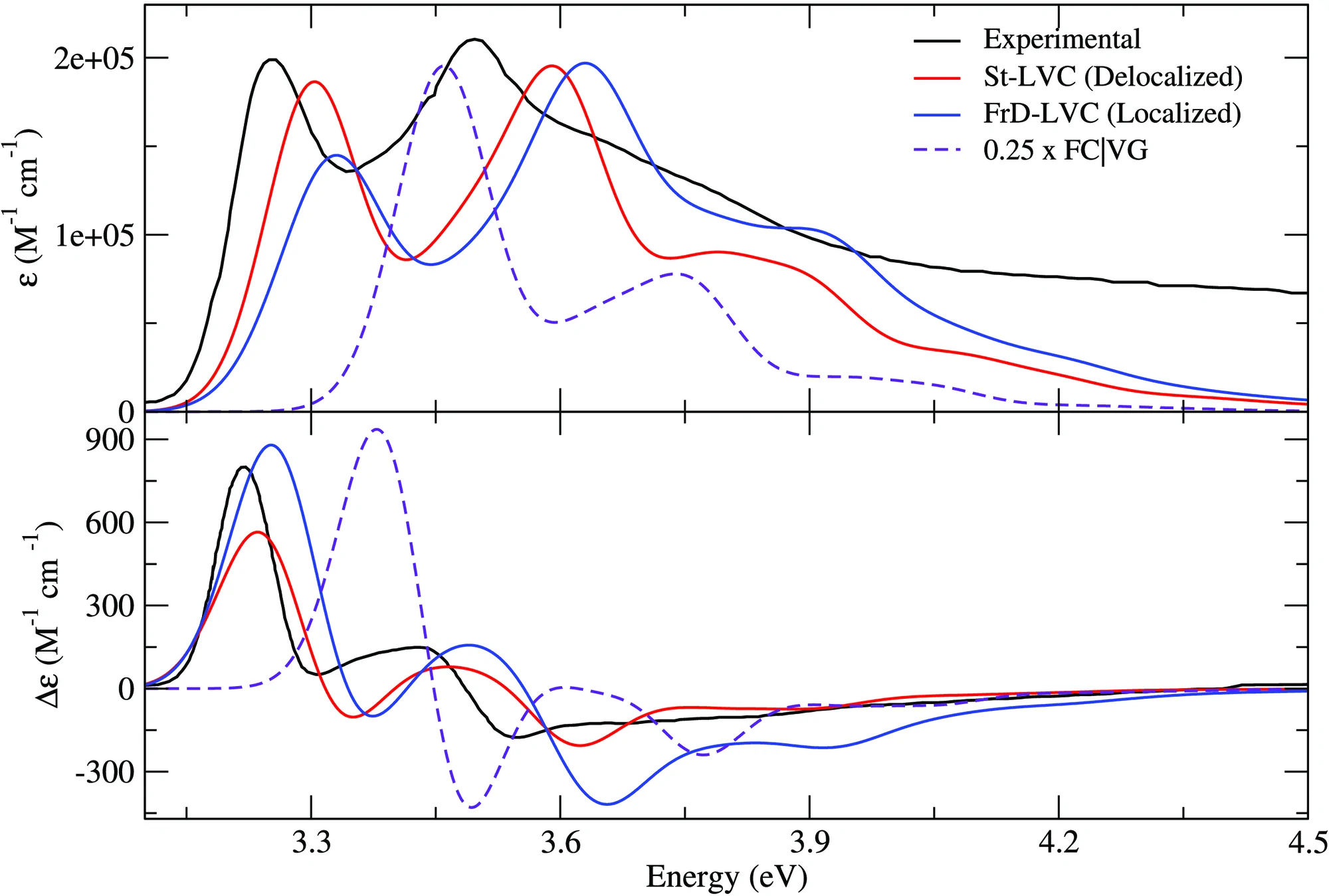
Comparison of computed and experimental ABS and ECD spectra for (*P*_4_)-**DES**_4_. Computed spectra were red-shifted by 0.06 eV, and a Gaussian broadening with HWHM = 0.06 eV was set for both ABS and ECD. Computed LVC OPA and ECD spectra were scaled by 0.5 and 0.25, respectively. CAM-B3LYP/6–31G­(d) level of theory.

### 4.5 Analyzing the Trend of Chiroptical Properties of the DESn Macrocycle with LVC Parameters

The systematic study presented in this work allows for an analysis of the trend of the chiroptical properties with the dimensions of DES macrocycles. More specifically, the success of the FrD-LVC exciton-like approach in reproducing the OPA and ECD spectra suggests that despite the covalent links, a rationalization of such properties can be obtained in terms of the properties and interactions of the idealized monomers that compose them. Finally, the idealized simplified models presented in Section 2.1 set an attractive framework for such analysis, suggesting the existence of some general correlation between the parameters ruling the spectroscopy of the different species.

The most evident relation between all systems concerns the extent of the linear couplings between the delocalized diabatic states of the macrocyles. The analysis in Section 2.1 suggests that they all have a common origin, namely, the gradient of the local excitation in the idealized monomer. It is striking that this prediction is fully confirmed by our St-LVC parametrization. For example, for the triple bond stretchings, for all species we can get a λ_(*n*)_ ≈ 0.32 eV, with (*P*_4_)-**DES**_4_ adjusting to this value better than (*P*_2_)-**DES**_2_ and (*P*_3_)-**DES**_3_. In fact, for (*P*_2_)-**DES**_2_, we have $\lambda_{\left(2\right)}=\lambda_{11\left(224\right)}\sqrt{2}\approx 0.31$ eV (data from Table 3), for (*P*_3_)-**DES**_3_, we have $\lambda_{\left(3\right)}=\lambda_{11\left(339\right)}\sqrt{3}\approx 0.31$ eV (data from Table S10 in the SI) and for (*P*_4_)-**DES**_4_, λ_(4)_ = λ_11(454)_2 = 0.32 eV­(data from Table 6). Notice that, according to the localized approach, which is parametrized in a fully independent way, λ is 0.39 eV for (*P*_2_)-**DES**_2_ and (*P*_3_)-**DES**_3_ and 0.38 eV for (*P*_4_)-**DES**_4_. Since these data confirm that the pure vibrational contribution of each monomer to the spectra of the macrocycles is very similar, the differences shall mainly arise from the different mutual arrangements of the monomers, affecting both the couplings and the combination of ETDMs and MTDMs. It is worth noticing that additional differences can actually arise from the intrinsic distortion imposed to the monomer geometry by the macrocyle structure

Computed excitonic couplings between adjacent monomers are larger for (*P*_2_)-**DES**_2_ and then seem to converge for (*P*_3_)-**DES**_3_ and (*P*_4_)-**DES**_4_. Such behavior can be easily rationalized with the fact that in (*P*_2_)-**DES**_2_, the two branches of the molecule form a smaller angle and are therefore closer in space than in the other macrocycles. For all species, moreover, couplings to CT states play a negligible role in determining the chiroptical properties, at least in the considered energy ranges.

In experiments, ECD intensities increase more than linearly with the number of fragments, as it is clearly shown in Figure S36 of the Supporting Information by plotting the intensities of the spectra of the different species normalized by division for the number of units *n*. This prediction is correctly reproduced by our computations, particularly for the localized approaches. The trend is a bit less clear for OPA, where these normalized intensities are similar for (*P*_2_)-**DES**_2_ and (*P*_3_)-**DES**_3_ and larger for (*P*_4_)-**DES**_4_, although the shape of (*P*_2_)-**DES**_2_ is much broader, making the comparison less straightforward. Our computations predict a more-than-linear increase of intensity with ring size for both OPA and ECD. For ECD, the trend holds across the whole series, whereas for OPA, the increase is marked from (*P*_2_)-**DES**_2_ to (*P*_3_)-**DES**_3_, but nearly saturates from (*P*_3_)-**DES**_3_ to (*P*_4_)-**DES**_4_.

Since both OPA and ECD increase to a similar extent, the experimental anisotropy factor *g*, plotted in Figure S38 of the SI, remains rather similar for the three species from 3.2 to 3.75 eV. Interestingly, *g* is larger at low energies since the first ECD peak is red-shifted with respect to the OPA one. The computed *g* is in remarkable agreement with experiment, apart from some larger fluctuations for (*P*_3_)-**DES**_3_ and (*P*_4_)-**DES**_4_ due to fact that the flat experimental ECD in the interval 3.3–3.45 eV is reproduced in the computation by two small but visible bands with opposite concavity (check Figures 4 and 5).

Another interesting difference from that of (*P*_2_)-**DES**_2_ to that of (*P*_4_)-**DES**_4_ concerns the contribution to the ECD due to the intrinsic chirality induced in each monomer by the bent geometry assumed in the macrocycle. The monomer is very bent in (*P*_2_)-**DES**_2_, moderately bent in (*P*_3_)-**DES**_3_ and almost planar in (*P*_4_)-**DES**_4_ (Figure S5 in SI). Consequently, it acquires a larger intrinsic MTDM in (*P*_2_)-**DES**_2_. Figure 6 actually shows that the impact of such intrinsic MTDM is negligible for (*P*_4_)-**DES**_4_, small for (*P*_3_)-**DES**_3_ and dominant for (*P*_2_)-**DES**_2_.

**Figure 6:**
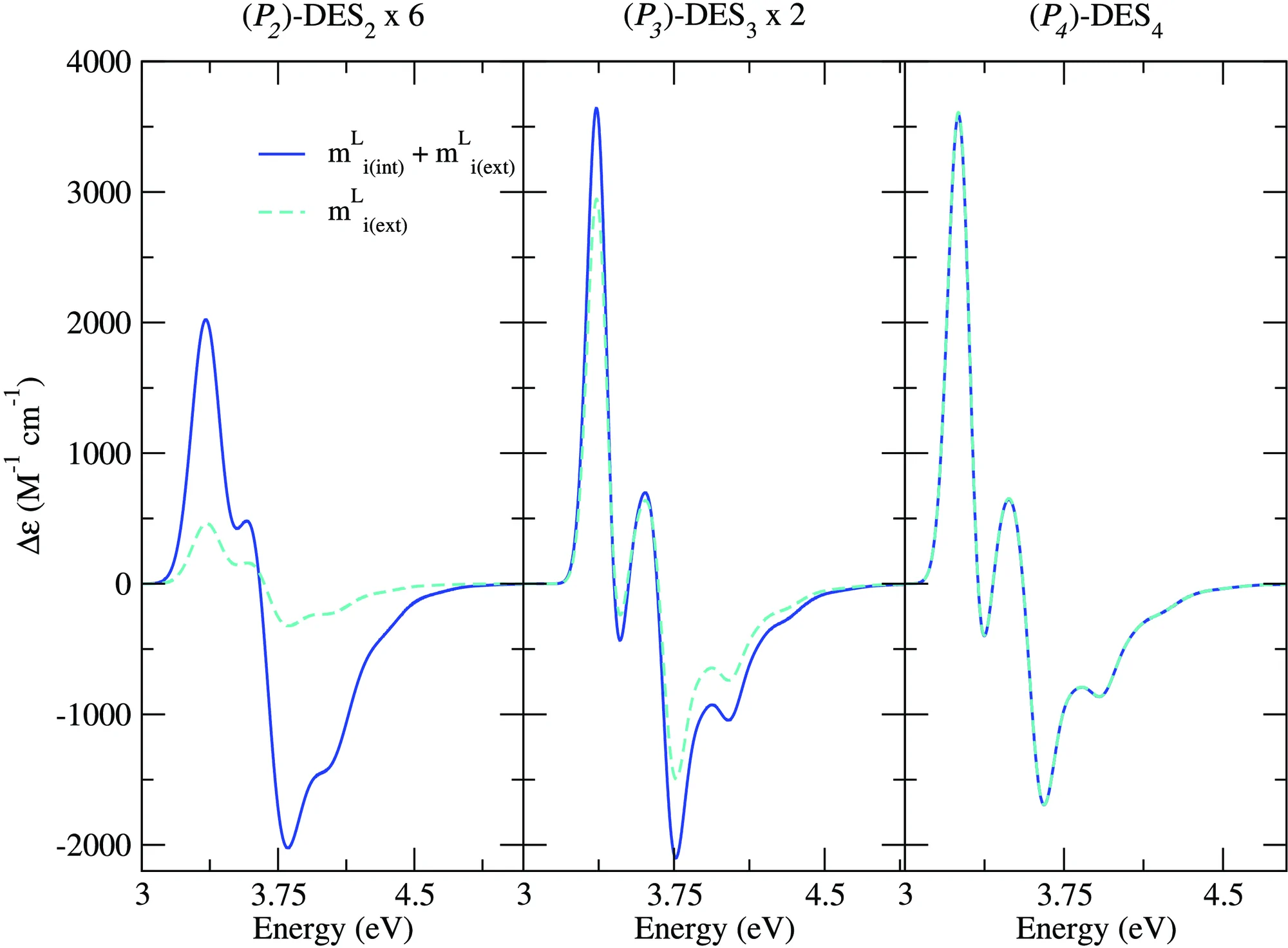
Comparison of ECD spectra computed with the localized FrD-LVC approach by taking into account both intrinsic and extrinsic (***m***_*i*(*int*)_^*L*^ + ***m***_*i*(*ext*)_^*L*^) and only extrinsic (***m***_*i*(*ext*)_^*L*^) contributions in the MTDM. In order to fit on the same scale, (*P*_2_)-**DES**_2_ and (*P*_3_)-**DES**_3_ intensities were multiplied by 6 and 2, respectively. CAM-B3LYP/6–31G­(d) level of theory.

The application of the exciton-like FrD-LVC approach offers an additional interesting point of view for the analysis of the different contributions to the ECD spectrum. Exploiting the expression for the magnetic transition dipole in eq 12, the scalar products in the expression for the ECD spectrum in eq 14, can be rewritten as

16 $$m_{i}^{L}\cdot \mu_{j}^{L}=m_{i\left(int\right)}^{L}\cdot \mu_{j}^{L}+m_{i\left(ext\right)}^{L}\cdot \mu_{j}^{L}$$

For *i* = *j* the second member of the rhs is zero because ***m***_*i*(*ext*)_^*L*^ is orthogonal to **μ**_*i*_^*L*^ (eq 12). This means that the ECD due to the external component of the MTDMs arises only from cross-correlation terms in eq 15, i.e. only thanks to couplings, because such cross-correlations only exist when a wavepacket starting in the local state *i* has a non vanishing probability to move to state *j*.

Therefore, the contributions of autocorrelation terms *i* = *j* arise only due to the intrinsic MTDM ***m***_*i*(*int*)_^*L*^, i.e., to the distortion of the monomer structure in the macrocycle, and it becomes progressively weaker from (*P*_2_)-**DES**_2_ to (*P*_3_)-**DES**_3_ and (*P*_4_)-**DES**_4_. This phenomenon is clearly shown in Figure S3 of the Supporting Information. On the other hand, the same figure highlights that the contribution of such autocorrelations remains weak even for (*P*_2_)-**DES**_2_, where ***m***_*i*(*int*)_^*L*^ is dominant. Figure S4 further evidences this point, showing that the ECD of (*P*_2_)-**DES**_2_ is largely due to the ***m***_*i*(*int*)_^*L*^ contribution to cross-correlations. Physically speaking, this fact indicates that such an ECD is not simply the one of the monomer inherited by the dimer, since it is in any case dominated by a contribution due to the excitonic interaction of the two monomers.

Turning to the analysis of the effect of the external component of the MTDMs to cross-correlation functions, the contribution of a pair of monomers *i* and *j* tends to increase with the dimension of the ring since the distance of the monomers from the center of masses becomes larger. Moreover, the number of pairs (*i*, *j*) grows quadratically, as *n*(*n* – 1), explaining why ECD intensities tend to increase with *n*.

The partition of the contributions to the spectra in autocorrelation and cross-correlation ones brings some information also for OPA spectra. In fact, it comes out that, only considering autocorrelation terms, the spectra of (*P*_2_)-**DES**_2_, (*P*_3_)-**DES**_3_, and (*P*_4_)-**DES**_4_ are all predicted to have a very similar shape (see Figure S3 in the SI). The differences are therefore mainly due to cross-correlation terms, which are more important for (*P*_2_)-**DES**_2_, given that the ETDMs of the two monomers are more collinear and therefore their scalar product is larger (see Table S6 in SI).

### 4.6 Design Principles

The analysis above can be distilled into transferable design principles for excitonic chiroptical macrocycles. In the localized picture, the ECD of a *D*_*n*_ macrocycle is governed by (i) the electric transition dipole of the monomer, |**μ**_*i*_^*L*^|, (ii) its distance from the center of mass, |**R**_**i**_|, (iii) the intrinsic MTDM of the strained monomer, ***m***_*i*(*int*)_^*L*^; (iv) the excitonic couplings β (adjacent) and η (nonadjacent), and (v) the monomer vibronic gradient λ.

Enlarging the macrocycle amplifies the extrinsic MTDM, which grows with |**R**_**i**_|(eq 12), while the number of cross-correlation pairs that generate the spectra grows as *n*(*n* – 1) (eq 16). Together, these produce the superlinear growth of the ECD with *n* documented above. The OPA, however, increases to a comparable extent, so the dissymmetry factor *g* remains nearly constant along the series (Figure S38). The intrinsic MTDM, by contrast, contributes appreciably only in the smallest and most bent macrocycle, so it is a lever for small rings rather than a route to scale up the response. Since also in these small rings the ECD arises from the interaction between the monomers, engineering macrocyle structures which maximize the scalar product of the intrinsic MTDM of one monomer with the ETDM of the other one, would favor the ECD response.

The lowest part of the ECD signal is made up by a positive/negative doublet and what protects it from self-cancellation is the energetic separation. At pure electronic level it is Δ*E* = 2β for (*P*_2_)-**DES**_2_, 3β for (*P*_3_)-**DES**_3_ and 2­(β + η) for (*P*_4_)-**DES**_4_. The very same expansion that increases |**R**_**i**_| moves the chromophores apart and weakens the coupling. The two levers thus pull in opposite directions, hence ring size is best treated as a quantity to be optimized rather than maximized. Crucially, in this series of DES macrocycles the computed splittings (*ΔE* = 0.15, 0.16, and 0.12 eV) are comparable to the width of the vibronic progressions, which places them in the regime where the cancellation of the positive/negative ECD peaks is decided by the band shape. This finding reveals a design lever that a purely electronic exciton treatment cannot access: what governs the observable ECD amplitude is not Δ*E* alone, but its ratio to the vibronic bandwidth, which in our localized model is determined by the single parameter λ. To test its importance, we recomputed the spectra scaling λ by 0.5 and 2.0 (Figure S37). Halving λ concentrates the oscillator and rotatory strength into a narrower progression, so that the two branches of the couplet overlap less, cancel less, and the ECD amplitude grows markedly; doubling λ produces the opposite effect. Therefore, at the same electronic coupling, chromophores with intrinsically narrow vibronic progressions are expected to deliver significantly higher *g*-factors.

Overall, the chiroptical response is maximized not by maximizing the ring size, but by maximizing the ratio between the exciton splitting and the vibronic bandwidth. One should (i) increase the monomer electric transition dipole |**μ**_*i*_^*L*^|, which raises both the intensity and, through β, the splitting that protects it; (ii) preserve a large β by not pushing the chromophores farther apart than necessary; and (iii) select stiff chromophores with a small λ.

## 5 Conclusions

In this work, we have exploited both delocalized and localized LVC models to investigate and explain experimental OPA and ECD spectra for three axially chiral macrocycles, (*P*_2_)-**DES**_2_, (*P*_3_)-**DES**_3_ and (*P*_4_)-**DES**_4_, made up by 2, 3, and 4 diethynylspirobifluorene units, respectively. The delocalized approach defines diabatic states from electronic states of the entire macrocycle and naturally describes Conical Intersections and Jahn–Teller and pseudo-Jahn–Teller effects, whereas the localized approach is based on the definition of local excitations on each branch of the macrocycle coupled through exciton-like interactions. We also introduced simplified idealized models which help in connecting the localized and delocalized pictures, emphasizing that the parameters of the Hamiltonians in the two representations are expected to be connected by simple relations based on symmetry.

Our results show that both localized and delocalized pictures reproduce very satisfactorily the chiroptical properties of these macrocycles, evidencing that the excited states are intrinsically coupled by nonadiabatic interactions which dominate the shapes of OPA and ECD spectra. On the other hand, we also clearly evidence that the cross comparison between localized and delocalized approaches can allow a deeper understanding of the chiroptical properties of axially chiral macrocycles.

The delocalized model predicts that both JT and pJT effects are present in (*P*_3_)-**DES**_3_, and only pJT effects play a role for (*P*_4_)-**DES**_4_, evidencing that they dominate over couplings between the nondegenerate states S_1_ and S_4_. On the other hand, the localized model allows for a comprehensive study of how the number of fragments influences OPA and ECD spectra. In particular, we have shown that the vibronic contributions of the fragments are essentially the same in all species, independent of the number of fragments. The differences observed in spectral shapes and intensities between the different macrocycles emerge mainly from the changes in electric and magnetic transition dipole moments arising from the strain of the fragments and the size of the molecules.

Our analysis indicates that the ECD spectrum of (*P*_2_)-**DES**_2_ is dominated by the intrinsic chirality of each monomer because of the strongly bent structure of the macrocycle. On the other hand, the excitonic contribution due to the interaction between the ETDMs of different monomers becomes more dominant as the number of fragments increases. Experimental ECD intensities increase more than linearly with the number of fragments *n* with this trend being correctly reproduced and explained by our computations. On balance, however, the anisotropy *g*-factor remains rather similar for the three investigated macrocycles.

Beyond rationalizing these trends, our results translate into design principles for excitonic chiroptical macrocycles. Enlarging the macrocycle amplifies the extrinsic magnetic transition dipole, but it simultaneously moves the chromophores apart and weakens the excitonic coupling that separates the two oppositely signed branches of the exciton couplet so that the ring size is a quantity to be optimized rather than maximized. More importantly, the computed exciton splittings are comparable to the width of the vibronic progressions, and therefore, the observed ECD amplitude is decided by the bandwidth. This vibronic lever is a direct result of the vibronic description adopted here.

From a computational point of view, the possibility to define from first-principle electronic computations a localized model Hamiltonian able to deliver accurate predictions, even for chemical structures in which the monomers are covalently bound, together with the capability to reduce the number of vibrational coordinates on the grounds of rigorous procedures like the hierarchical representation, suggests that these refined localized approaches can be profitably adopted also for more general and extended nanostructures exhibiting axial chirality.^43–45^

In conclusion, our localized nonadiabatic model allows for a fast check of the combined effect of electronic couplings and vibrational gradients, and therefore, it stands as a fast predictive tool to screen the expected chiroptical properties of possible candidate macrocycles even before synthesis.

## Supplementary Material





## Data Availability

Optimized structures and parameters of LVC Hamiltonians are available at the Zenodo database: 10.5281/zenodo.21624829.^46^ The open source code Overdia employed to obtain the corresponding LVC parameters can be obtained at http://www.iccom.cnr.it/en/overdia-en/.

## Notes

Movies for the most important modes for each species (ZIP)
Further theoretical remarks regarding different strategies to compute localized MTDMs and a comparison between them, examination of the influence of auto and cross-correlation contributions to OPA and ECD spectra, angles and overlaps between ETDM and MTDM vectors for the localized model, data for the idealized monomers, ML-MCTDH trees, more details on adiabatic PES, an extended discussion on (*P*_3_)-**DES**_3_, population dynamics, block convergence for the hierarchical model, comparison of spectra obtained through the standard and hierarchical strategies for the delocalized model, investigation of the dependence of OPA and ECD intensities on the number of fragments, effect of the vibronic contribution on the broadening of spectra, and *g*-factors (PDF)
