# Supplementary material for: Localized and Delocalized Nonadiabatic Vibronic Models Unravel Chiroptical Trends and Design Principles in Symmetric π Conjugated Macrocycles
Source: J Chem Theory Comput. 2026 Oct 19;22(21):11484–97. doi: 10.1021/acs.jctc.6c01519 (PMC13757766; doi:10.1021/acs.jctc.6c01519)
Supplement: Supplementary file 1 [file ct6c01519_si_002.pdf]

Supporting Information: Localized and Delocalized Nonadiabatic Vibronic Models Unravel Chiroptical Trends and Design Principles in Symmetric π Conjugated Macrocycles

Francisco García-González,[†,‡] Julia Portela-Pino,[¶,§] Daniel Aranda,[†,‡] José Lorenzo Alonso-Gómez,[¶] and Fabrizio Santoro[*,‖]

†*Andalucía Tech, Facultad de Ciencias, Departamento de Química Física, Universidad de Málaga, 29071-Málaga, Spain*

‡*Instituto Universitario de Materiales y Nanotecnología, IMANA, University of Malaga, Campus de Teatinos 29071, Malaga, Spain*

¶*Department of Organic Chemistry, University of Vigo, 36310 Vigo, Spain*

§*Department of Physical Chemistry, Lagoas-Marcosende, 36310, Vigo, Spain*

‖*Istituto di Chimica dei Composti Organometallici (ICCOM-CNR), Area della Ricerca del CNR, Via Moruzzi 1, I-56124 Pisa, Italy*

E-mail: fabrizio.santoro@pi.iccom.cnr.it

Summary

1 Further theoretical remarks **S-4**

1.1 Computation of Magnetic Transition Dipole Moments in the localized picture S-4

1.2 Origin Invariance of the computation of ECD spectra in a localized picture S-13

2 Further Computational Details **S-15**

2.1 Delocalized Picture S-15

2.2 Localized Picture S-16

2.3 ML-MCTDH Trees S-18

3 Potential Energy Surfaces **S-22**

3.1 (P_2)-$\mathbf{DES}_2$ S-22

3.1.1 On the absence of Conical Intersections in (P_2)-$\mathbf{DES}_2$ with our model S-23

3.2 (P_3)-$\mathbf{DES}_3$ S-24

3.3 (P_4)-$\mathbf{DES}_4$ S-26

4 A more Extended Discussion on (P_3)-$\mathbf{DES}_3$ LVC and Spectra **S-31**

4.0.1 OPA and ECD Spectra. S-32

5 Extended LVC Parameters of (P_4)-$\mathbf{DES}_4$ **S-34**

6 Dynamics of the Diabatic Electronic Populations for all Species **S-35**

7 Further results with the delocalized St-LVC model **S-38**

7.1 Block convergence for the Hierarchy model S-38

7.2 Comparison between Hierarchy and St-LVC methodologies S-41

7.3 Investigating the non-linear dependence of intensities on the number of fragments S-44
7.4 Broadening related to vibronic progressions S-46
7.5 g-Factors S-47

8 Movies of the main vibrational modes S-48

References S-49

1 Further theoretical remarks

1.1 Computation of Magnetic Transition Dipole Moments in the localized picture

This section presents the different strategies that were investigated to compute the Magnetic Transition Dipole Moments (MTDMs) for the localized picture. Coordinate axes for each species are those indicated in the main manuscript (Figure 1). Firstly, using the eigenvectors that transform localized matrices $\mathbf{H}^{vib,L}_{dia(m)}(\mathbf{0})$ into delocalized matrices $\mathbf{H}^{vib,D}_{dia(m)}(\mathbf{0})$ and vice-versa:

$$\mathbf{T}_{(2)} = \begin{pmatrix} \frac{1}{\sqrt{2}} & -\frac{1}{\sqrt{2}} \\ \frac{1}{\sqrt{2}} & \frac{1}{\sqrt{2}} \end{pmatrix} \tag{1}$$

$$\mathbf{T}_{(3)} = \begin{pmatrix} \frac{1}{\sqrt{3}} & -\frac{1}{\sqrt{6}} & \frac{1}{\sqrt{2}} \\ \frac{1}{\sqrt{3}} & \sqrt{\frac{2}{3}} & 0 \\ \frac{1}{\sqrt{3}} & -\frac{1}{\sqrt{6}} & -\frac{1}{\sqrt{2}} \end{pmatrix} \tag{2}$$

$$\mathbf{T}_{(4)} = \begin{pmatrix} \frac{1}{2} & -\frac{1}{\sqrt{2}} & 0 & \frac{1}{2} \\ \frac{1}{2} & 0 & -\frac{1}{\sqrt{2}} & -\frac{1}{2} \\ \frac{1}{2} & \frac{1}{\sqrt{2}} & 0 & \frac{1}{2} \\ \frac{1}{2} & 0 & \frac{1}{\sqrt{2}} & -\frac{1}{2} \end{pmatrix} \tag{3}$$

$$\mathbf{H}^{vib,D}_{dia(2)}(\mathbf{0}) = \mathbf{T}^{T}_{(2)}\mathbf{H}^{vib,L}_{dia(2)}(\mathbf{0})\mathbf{T}_{(2)} = \mathbf{T}^{T}_{(2)} \begin{pmatrix} E_{0(2)} & \beta_{(2)} \\ \beta_{(2)} & E_{0(2)} \end{pmatrix} \mathbf{T}_{(2)} = \begin{pmatrix} E_{0(2)}+\beta_{(2)} & 0 \\ 0 & E_{0(2)}-\beta_{(2)} \end{pmatrix} \tag{4}$$

$$\mathbf{H}^{vib,D}_{dia(3)}(\mathbf{0}) = \begin{pmatrix} E_{0(3)}+2\beta_{(3)} & 0 & 0 \\ 0 & E_{0(3)}-\beta_{(3)} & 0 \\ 0 & 0 & E_{0(3)}-\beta_{(3)} \end{pmatrix} \tag{5}$$

$$\mathbf{H}^{vib,D}_{dia(4)}(\mathbf{0}) = \begin{pmatrix} E_{0(4)}+2\beta_{(4)}+\eta_{(4)} & 0 & 0 & 0 \\ 0 & E_{0(4)}-\eta_{(4)} & 0 & 0 \\ 0 & 0 & E_{0(4)}-\eta_{(4)} & 0 \\ 0 & 0 & 0 & E_{0(4)}-2\beta_{(4)}+\eta_{(4)} \end{pmatrix} \tag{6}$$

It is possible to compute localized ETDMs and MTDMs from the delocalized vectors by applying:

$$\mu_\xi^{loc} = \mathbf{T}_{(n)} \mu_\xi^{del} \tag{7}$$

$$m_\xi^{loc} = \mathbf{T}_{(n)} m_\xi^{del} \tag{8}$$

Where $\mu_\xi^{loc(del)}$ and $m_\xi^{loc(del)}$ are the ETDM and MTDM column vectors for $\xi = x, y, z$. Results are shown in Table S1.

Table S1: Localized electric transition dipole moments (μ^{loc}) and magnetic dipole moments (m^{loc}) obtained by applying transformation matrices $\mathbf{T}_{(n)}$ to delocalized ETDMs and MTDMs for each of the monomers for the species studied in this work. All values in a.u. except for R_{vel} (10^{-40} cgs). CAM-B3LYP/6-31G(d) level of theory.

	$\boldsymbol{\mu}^{loc}$				$\mathbf{m}^{loc}$					
	X	Y	Z	$\|\mu\|$	X	Y	Z	$\|m\|$	$\|\mu\|^2$	R_{vel}
(P_2)-**DES**$_2$										
Mon. 1	2.98	0.00	0.78	3.08	-0.71	0.00	2.37	2.47	9.49	-130
Mon. 2	-2.98	0.00	0.78	3.08	0.71	0.00	2.37	2.47	9.49	-130
(P_3)-**DES**$_3$										
Mon. 1	-4.09	0.00	0.84	4.18	0.71	0.00	3.31	3.39	17.47	-86
Mon. 2	2.05	3.54	0.84	4.18	-0.35	-0.62	3.31	3.39	17.47	-86
Mon. 3	2.05	-3.54	0.84	4.18	-0.35	0.62	3.31	3.39	17.47	-86
(P_4)-**DES**$_4$										
Mon. 1	3.10	3.10	0.85	4.47	-0.52	-0.52	4.28	4.34	19.98	-109
Mon. 2	-3.10	3.10	0.85	4.47	0.52	-0.52	4.28	4.34	19.98	-109
Mon. 3	-3.10	-3.10	0.85	4.47	0.52	0.52	4.28	4.34	19.98	-109
Mon. 4	3.10	-3.10	0.85	4.47	-0.52	0.52	4.28	4.34	19.98	-109

It can also be considered to extract ETDMs and MTDMs directly from the localized diabatic states obtained by applying the transformation matrices $\mathbf{T}_{(m)}$ to the m HOMO-LUMO orbitals of the molecule, with m being the number of fragments. Vectors computed this way are shown in Table S2.

An alternative way to obtain MTDMs is by extracting them directly from the

Table S2: Electric transition dipole moments (μ_{HL}) and magnetic dipole moments (m_{HL}) obtained by applying transformation matrices $\mathbf{T}_{(n)}$ to the first n HOMO-LUMO orbitals for the species studied in this work. All values in a.u. except for R_{vel} (10^{-40} cgs). CAM-B3LYP/6-31G(d) level of theory.

	$\boldsymbol{\mu}_{HL}$				$\mathbf{m}_{HL}$					
	X	Y	Z	$\lvert\mu\rvert$	X	Y	Z	$\lvert m\rvert$	$\lvert\mu\rvert^2$	R_{vel}
(P_2)-**DES**$_2$										
Mon. 1	3.69	0.00	0.94	3.84	-0.86	0.00	2.45	2.59	14.71	-435
Mon. 2	-3.69	0.00	0.94	3.84	0.86	0.00	2.45	2.59	14.71	-435
(P_3)-**DES**$_3$										
Mon. 1	-4.70	0.00	1.01	4.81	0.93	0.00	3.79	3.90	23.11	-376
Mon. 2	2.35	4.07	1.01	4.81	-0.46	-0.80	3.79	3.90	23.11	-376
Mon. 3	2.35	-4.07	1.01	4.81	-0.46	0.80	3.79	3.90	23.11	-376
(P_4)-**DES**$_4$										
Mon. 1	3.39	3.39	1.05	4.91	-0.75	-0.75	3.60	3.75	24.09	-355
Mon. 2	-3.39	3.39	1.05	4.91	0.75	-0.75	3.60	3.75	24.09	-355
Mon. 3	-3.39	-3.39	1.05	4.91	0.75	0.75	3.60	3.75	24.09	-355
Mon. 4	3.39	-3.39	1.05	4.91	-0.75	0.75	3.60	3.75	24.09	-355

TD-DFT state of each monomer displaced at its corresponding position in the macrocycle (Table S3). It is worth mentioning that in this case this approach to compute MTDMs is equivalent to the application of the model according to exciton chirality theory.[S1,S2]

As indicated in the main manuscript, MTDMs are calculated through this approach as:

$$\boldsymbol{m}_i^L = \boldsymbol{m}_{i(int)}^L + \frac{ie\hbar}{2m_e c}\mathbf{R_i} \times \boldsymbol{\mu}_i^{vel} = \boldsymbol{m}_{i(int)}^L + \boldsymbol{m}_{i(ext)}^L \tag{9}$$

Where $\boldsymbol{m}_{i(int)}^L$ is the MTDM for the monomer placed in the center of mass, $\boldsymbol{m}_{i(ext)}^L$ is the extrinsic MTDM, $\boldsymbol{\mu}_i^{vel}$ is the ETDM in the velocity gauge formulation, and $\mathbf{R_i}$ is the position of the center of mass of the monomer. Table S4 shows $\boldsymbol{m}_{i(ext)}^L$ and positions for each of the monomers.

Table S3: Electric transition dipole moments (μ_{disp}) and magnetic dipole moments (m_{disp}) obtained for each of the monomers displaced to its position in the macrocycle for the species studied in this work. All values in a.u. except for R_{vel} (10^{-40} cgs). CAM-B3LYP/6-31G(d) level of theory.

	$\boldsymbol{\mu}_i^L$				$\boldsymbol{m}_i^L$					
	X	Y	Z	$\|\mu\|$	X	Y	Z	$\|m\|$	$\|\mu\|^2$	R_{vel}
(P_2)-**DES**$_2$										
Mon. 1	3.39	0.01	1.05	3.55	-0.72	0.00	1.97	2.09	12.60	-179
Mon. 2	-3.39	0.01	1.05	3.55	0.72	0.00	1.97	2.09	12.60	-179
(P_3)-**DES**$_3$										
Mon. 1	-4.27	0.00	1.20	4.44	0.89	0.00	2.98	3.11	19.66	-136
Mon. 2	2.14	3.70	1.20	4.44	-0.45	-0.77	2.98	3.11	19.66	-136
Mon. 3	2.14	-3.70	1.20	4.44	-0.45	0.77	2.98	3.11	19.66	-136
(P_4)-**DES**$_4$										
Mon. 1	3.14	3.14	1.20	4.60	-0.75	-0.75	3.93	4.07	21.26	-8
Mon. 2	-3.14	3.14	1.20	4.60	0.75	-0.75	3.93	4.07	21.26	-8
Mon. 3	-3.14	-3.14	1.20	4.60	0.75	0.75	3.93	4.07	21.26	-8
Mon. 4	3.14	-3.14	1.20	4.60	-0.75	0.75	3.93	4.07	21.26	-8

Table S4: Extrinsic magnetic dipole moments (m_{extr}) and positions (**R**) for each of the monomers displaced to its position in the macrocycle for the species studied in this work. $\boldsymbol{m}_{i(ext)}^L$ in a.u. and **R** in Å. CAM-B3LYP/6-31G(d) level of theory.

	$\boldsymbol{m}_{i(ext)}^L$				**R**			
	X	Y	Z	$\|\boldsymbol{m}_{i(ext)}^L\|$	X	Y	Z	$\|\mathbf{R}\|$
(P_2)-**DES**$_2$								
Mon. 1	-0.13	0.00	0.41	0.43	0.00	-0.91	0.00	0.91
Mon. 2	0.13	0.00	0.41	0.43	0.00	0.91	0.00	0.91
(P_3)-**DES**$_3$								
Mon. 1	0.68	0.00	2.40	2.49	0.00	4.34	0.00	4.34
Mon. 2	-0.34	-0.59	2.40	2.49	3.76	-2.17	0.00	4.34
Mon. 3	-0.34	0.59	2.40	2.49	-3.76	-2.17	0.00	4.34
(P_4)-**DES**$_4$								
Mon. 1	-0.75	-0.75	4.01	4.15	5.00	-5.00	0.00	7.07
Mon. 2	0.75	-0.75	4.01	4.15	5.00	5.00	0.00	7.07
Mon. 3	0.75	0.75	4.01	4.15	-5.00	5.00	0.00	7.07
Mon. 4	-0.75	0.75	4.01	4.15	-5.00	-5.00	0.00	7.07

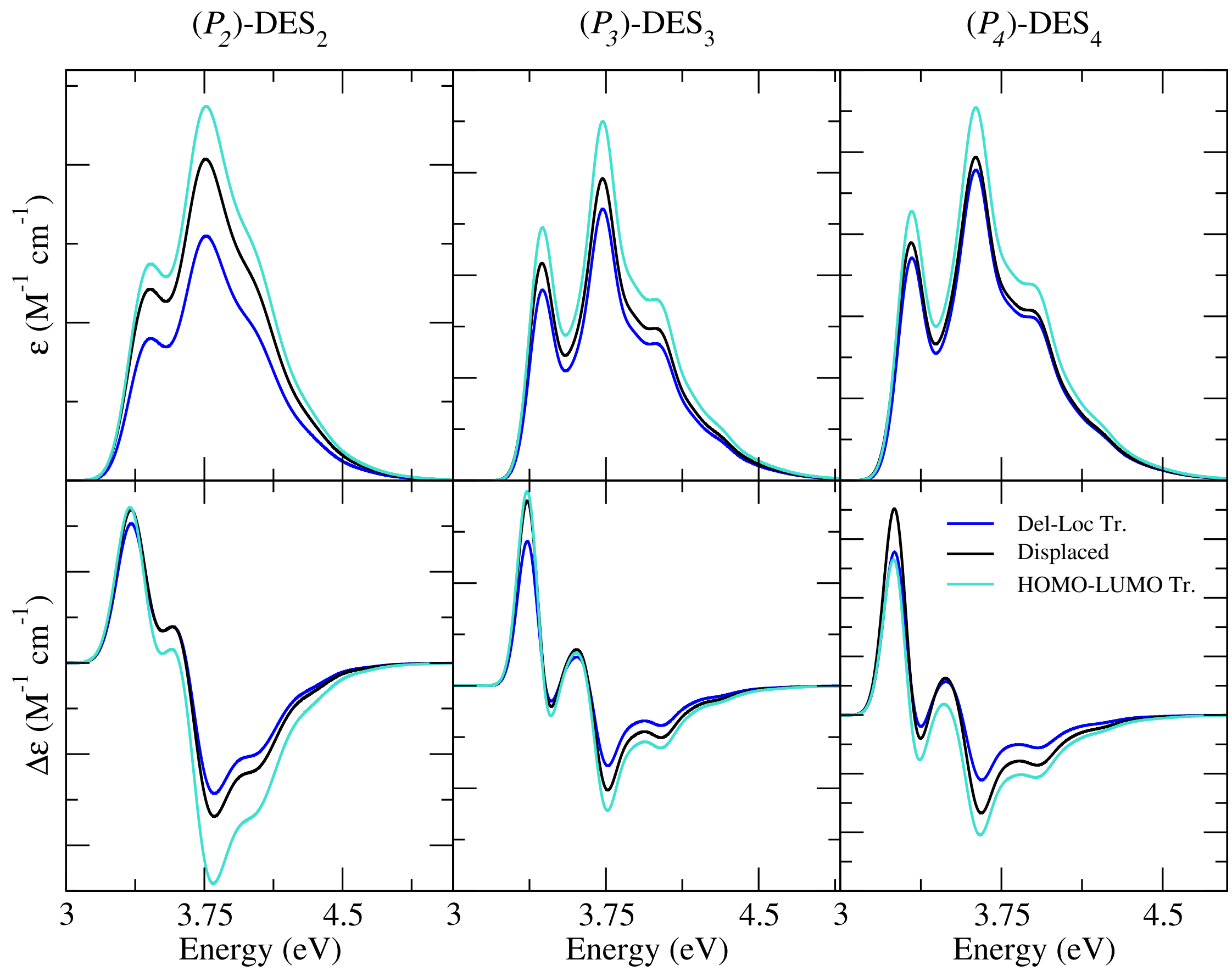

Figure S1: Comparison of OPA and ECD spectra by using ETDMs and MTDMs computed with different strategies: performing a transformation of ETDM and MTDMs through the delocalized-to-localized transformation matrix (Del-Loc Tr.), displacing the center of mass of the monomer to its corresponding position in the macrocycle (Displaced), and applying the transformation matrices $\mathbf{T}_{(m)}$ to the m HOMO-LUMO orbitals of each species. (HOMO-LUMO Tr.). Broadening set to 12.00, 18.26 and 17.00 fs for (P_2)-$\mathbf{DES}_2$, (P_3)-$\mathbf{DES}_3$ and (P_4)-$\mathbf{DES}_4$, respectively. CAM-B3LYP/6-31G(d) level of theory.

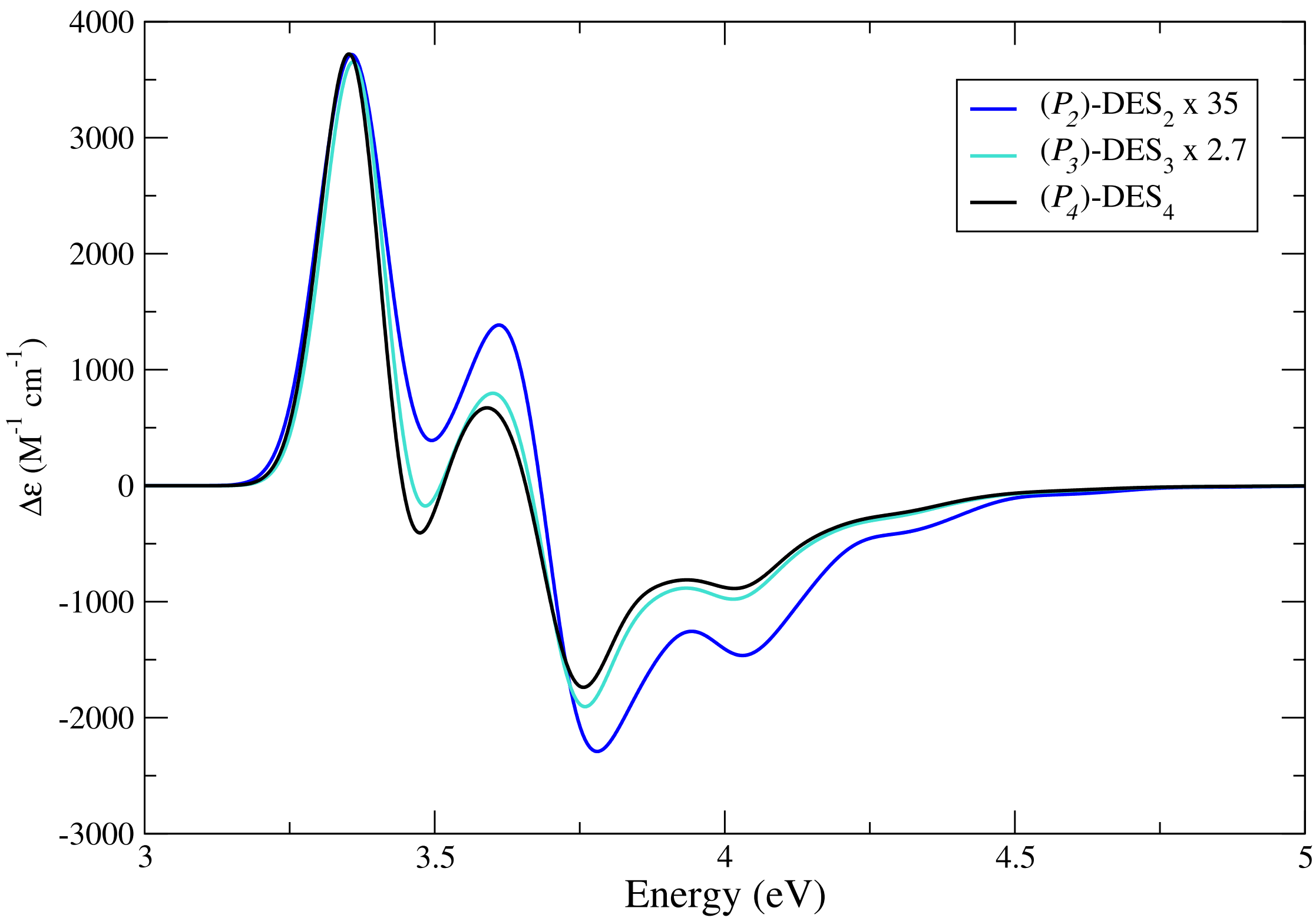

Figure S2: Comparison of ECD spectra by taking into account only extrinsic (m_{extr}) contributions in the MTDM. The energy domain of the spectra have been shifted so that they overlap as much as possible. Damping factor set to 17fs for all spectra. CAM-B3LYP/6-31G(d) level of theory.

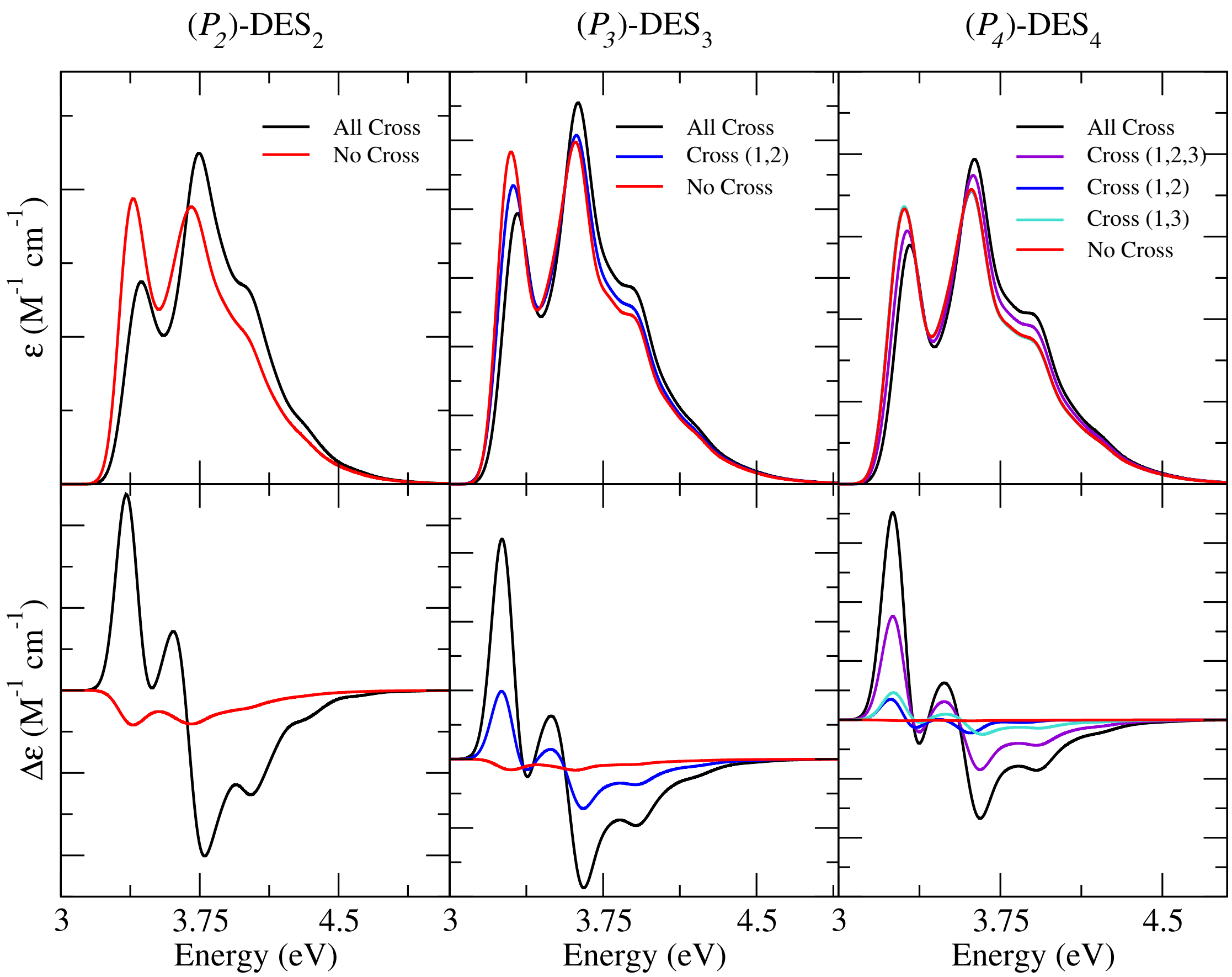

Figure S3: Comparison of OPA and ECD spectra by taking into account different cross-correlation functions. Cross(1,2) denotes cross-correlation functions between adjacent fragments, whereas Cross(1,3) refers to opposite fragments. Damping factor set to 17 fs in all cases. CAM-B3LYP/6-31G(d) level of theory.

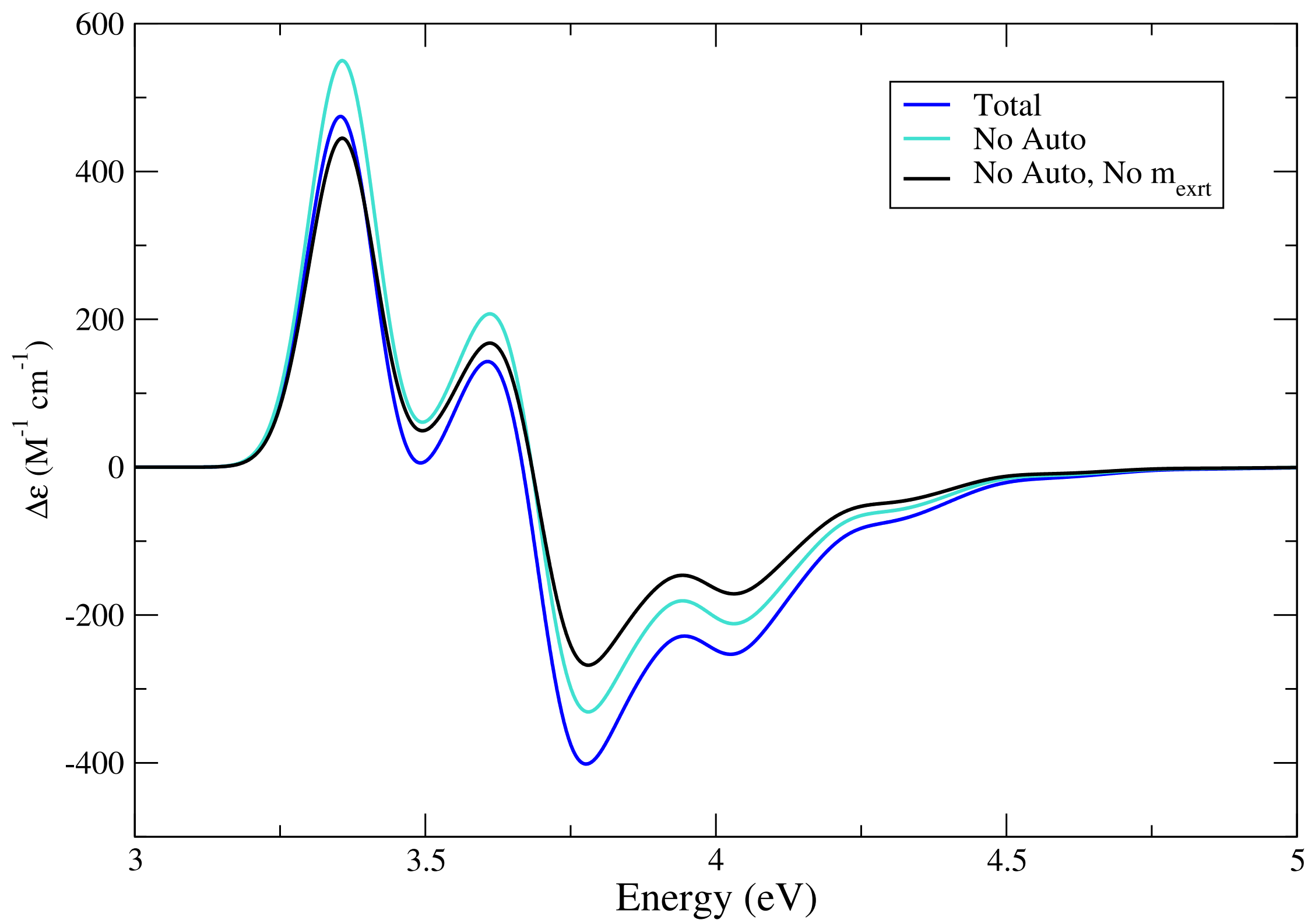

Figure S4: Comparison of ECD spectra for (P_2)-$\mathbf{DES_2}$ by taking into account the full spectrum, only cross-correlation contributions and only cross-correlation contributions excluding m_{extr}. Damping factor set to 17 fs in all cases. CAM-B3LYP/6-31G(d) level of theory.

Table S5: Dot products between $\boldsymbol{m}^L_{a(ext)}/\boldsymbol{m}^L_a$ (Monomer a) and $\boldsymbol{\mu}^L_b$ (Monomer b), and angles between the vectors ($\theta_{a(ext)-(b)}/\theta_{a-b}$) for the monomers integrating all the species studied in this work. All vector products in a.u. CAM-B3LYP/6-31G(d) level of theory.

	$\boldsymbol{m}^L_{a(ext)} \cdot \boldsymbol{\mu}^L_b$	$\theta_{a(ext)-(b)}$	$\boldsymbol{m}^L_a \cdot \boldsymbol{\mu}^L_b$	θ_{a-b}
(P_2)-**DES**$_2$				
Mons. 1,2	0.87	55.20º	4.51	52.71º
(P_3)-**DES**$_3$				
Mons. 1,2	4.33	66.95º	5.48	66.61º
(P_4)-**DES**$_4$				
Mons. 1,2	4.81	75.4º	4.81	75.4º
Mons. 1,3	9.52	60.0º	9.52	60.0º

Table S6: Dot products between $\boldsymbol{\mu}^L_a$ (Monomer a) and $\boldsymbol{\mu}^L_b$ (Monomer b) and angles between them for the monomers integrating all the species studied in this work. All vector products in a.u. CAM-B3LYP/6-31G(d) level of theory.

	$\boldsymbol{\mu}^L_a \cdot \boldsymbol{\mu}^L_b$	θ_{a-b}
(P_2)-**DES**$_2$		
Mons. 1,1	12.60	0.00º
Mons. 1,2	-10.39	145.58º
(P_3)-**DES**$_3$		
Mons. 1,1	19.71	0.00º
Mons. 1,2	-7.67	112.90º
(P_4)-**DES**$_4$		
Mons. 1,1	21.16	0.00º
Mons. 1,2	1.44	86.09º
Mons. 1,3	-18.28	149.76º

1.2 Origin Invariance of the computation of ECD spectra in a localized picture

Eqs 14 and 15 in the main text show that the ECD spectrum ($\Delta\epsilon(\omega)$) is a sum of contributions from auto- and cross-correlation functions multiplied by scalar products between some ETDM and MTDM. We focus on the localized picture according which the MTDM is given by the sum of two terms an intrinsic component ($\boldsymbol{m}^L_{i(int)}$) and an extrinsic component ($\boldsymbol{m}^L_{i(ext)}$) which is dependent on the origin of the coordinates (Eq. 12 in the main text, since now on we skip for simplicity the "L" superscript). The contribution of auto-correlation functions c_{ii} is independent of the coordinates of the system. In fact $\boldsymbol{\mu}_i$ is by definition orthogonal to the extrinsic contribution $\boldsymbol{m}_{i(ext)}$, that arises from the displacement of the monomer from the origin of coordinates, therefore the product $(\boldsymbol{m}_i \cdot \boldsymbol{\mu}_i)$ remains unchanged as the system is displaced.

For cross-correlation functions c_{ij}, we can examine the conjoint contribution of some pair c_{ij} and c_{ji}, $c_{ij}(\boldsymbol{m}_i \cdot \boldsymbol{\mu}_j) + c_{ji}(\boldsymbol{m}_j \cdot \boldsymbol{\mu}_i) = C_{ij}$, as:

$$C_{ij} = c_{ij}(\boldsymbol{m}_{i(int)} + \boldsymbol{m}_{i(ext)}) \cdot \boldsymbol{\mu}_j + c_{ji}(\boldsymbol{m}_{j(int)} + \boldsymbol{m}_{j(ext)}) \cdot \boldsymbol{\mu}_i \qquad (10)$$

The contribution from $\boldsymbol{m}_{i,j(int)}$ is independent of the position of the system. Turning to the extrinsic component, $\boldsymbol{m}_{i(ext)}$, it is:

$$\boldsymbol{m}_{i(ext)} = p_f(\boldsymbol{R}_i \times \boldsymbol{\mu}_i) \qquad (11)$$

Where $p_f = ie\hbar/2m_ec$ is the numerical prefactor present in Eq. 9. We assume that $\boldsymbol{R}_i$ is the position of the monomer when the center of mass of the macrocycle is placed the origin of coordinates. With these settings, the contribution of the pair is:

$$C_{ij} = c_{ij}(\boldsymbol{m}_{i(int)} + p_f(\boldsymbol{R}_i \times \boldsymbol{\mu}_i)) \cdot \boldsymbol{\mu}_j + c_{ji}(\boldsymbol{m}_{j(int)} + p_f(\boldsymbol{R}_j \times \boldsymbol{\mu}_j)) \cdot \boldsymbol{\mu}_i \tag{12}$$

If the system is displaced by some vector $\boldsymbol{r}$, we get:

$$\boldsymbol{m}^r_{i(ext)} = p_f((\boldsymbol{R}_i + \boldsymbol{r}) \times \boldsymbol{\mu}_i) \tag{13}$$

where the r superscript denotes that the system has been displaced by such vector. After applying the displacement, the contributions by some pair of cross-correlation functions are:

$$C^r_{ij} = c_{ij}(\boldsymbol{m}_{i(int)} + p_f((\boldsymbol{R}_i + r) \times \boldsymbol{\mu}_i)) \cdot \boldsymbol{\mu}_j + c_{ji}(\boldsymbol{m}_{j(int)} + p_f((\boldsymbol{R}_j + r) \times \boldsymbol{\mu}_j)) \cdot \boldsymbol{\mu}_i \tag{14}$$

which can be reordered as:

$$C^r_{ij} = C_{ij} + c_{ij} p_f(r \times \boldsymbol{\mu}_i) \cdot \boldsymbol{\mu}_j + c_{ji} p_f(\boldsymbol{r} \times \boldsymbol{\mu}_j) \cdot \boldsymbol{\mu}_i \tag{15}$$

Due to the symmetry of the system, $c_{ij} = c_{ji}$ in the localized picture, and exploiting the fact that

$$(\boldsymbol{r} \times \boldsymbol{\mu}_i) \cdot \boldsymbol{\mu}_j = -(\boldsymbol{r} \times \boldsymbol{\mu}_j) \cdot \boldsymbol{\mu}_i \tag{16}$$

we can conclude that

$$C^r_{ij} = C_{ij} \tag{17}$$

2 Further Computational Details

2.1 Delocalized Picture

Table S7: Electric (μ) and magnetic (m) transition dipole moment for the relevant excited states of the molecules investigated in this work. All values in a.u. CAM-B3LYP/6-31G(d) level of theory.

	μ			m		
	X	Y	Z	X	Y	Z
(P_2)-**DES**$_2$						
S_1	0.0	0.00	1.11	0.00	0.00	3.35
S_2	4.21	0.00	0.00	-1.01	0.00	0.00
(P_3)-**DES**$_3$						
S_1	0.0	0.00	1.45	0.00	0.00	5.74
S_2	-5.00	0.00	0.00	0.88	0.00	0.00
S_3	0.00	-5.00	0.00	0.00	-0.88	0.00
(P_4)-**DES**$_4$						
S_1	0.0	0.00	1.70	0.00	0.00	8.56
S_2	4.39	-4.39	0.00	-0.75	0.75	0.00
S_3	4.39	4.39	0.00	-0.75	-0.75	0.00
S_4	0.00	0.00	0.00	0.00	0.00	0.00

2.2 Localized Picture

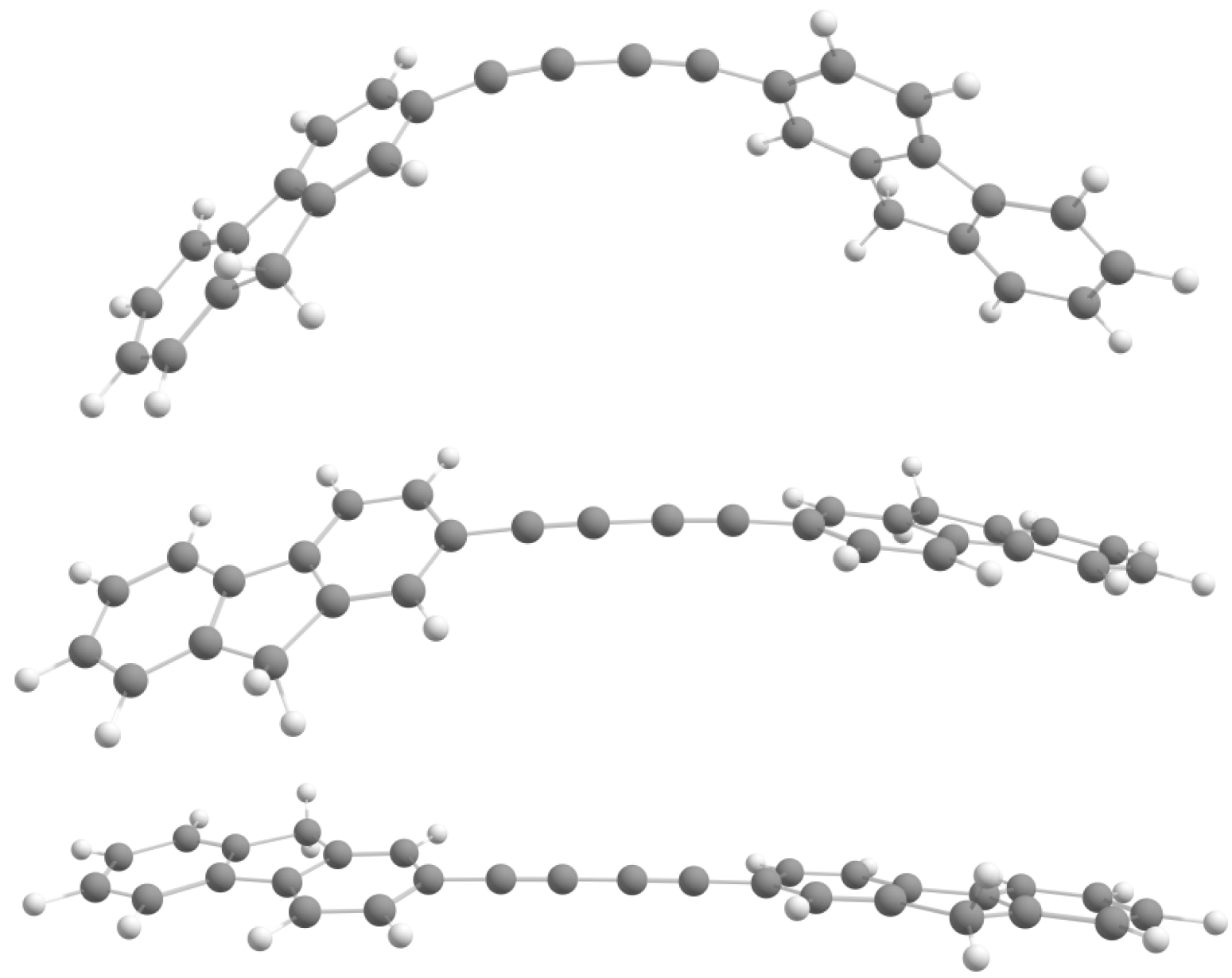

Figure S5: Saturated monomers employed to perform the calculations for the localized FrD-LVC methodology: (P_2)-$\mathbf{DES}_2$ (top), (P_3)-$\mathbf{DES}_3$ (mid) and (P_4)-$\mathbf{DES}_4$ (bottom). CAM-B3LYP/6-31G(d) level of theory.

Table S8: S_1 Vertical Energies (VE), Dipole Strengths, electric transition dipole moments (μ) and intrinsic magnetic dipole moments (m) for each of the monomers for the species studied in this work and the optimized monomer. Values taken with the center of mass of the molecule at the origin of coordinates. All values in a.u. except energies (eV) and R_{vel} (10^{-40} cgs). All data retrieved directly from TD-DFT calculations at the CAM-B3LYP/6-31G(d) level of theory.

		$\boldsymbol{\mu}$				$\mathbf{m}_{int}$					
	VE	X	Y	Z	$\|\mu\|$	X	Y	Z	$\|m_{int}\|$	$\|\mu\|^2$	R_{vel}
(P_2)-$\mathbf{DES_2}$											
Mon. 1	3.86	3.39	0.01	1.05	3.55	-0.59	0.00	1.60	1.71	12.60	-179
Mon. 2	3.86	-3.39	0.01	1.05	3.55	0.59	0.00	1.60	1.71	12.60	-179
(P_3)-$\mathbf{DES_3}$											
Mon. 1	3.72	-4.27	0.00	1.20	4.44	0.23	0.00	0.62	0.66	19.66	-136
Mon. 2	3.72	2.14	3.70	1.20	4.44	-0.12	-0.20	0.62	0.66	19.66	-136
Mon. 3	3.72	2.14	-3.70	1.20	4.44	-0.12	0.20	0.62	0.66	19.66	-136
(P_4)-$\mathbf{DES_4}$											
Mon. 1	3.68	3.14	3.14	1.20	4.60	0.00	0.00	-0.02	0.02	21.26	-8
Mon. 2	3.68	-3.14	3.14	1.20	4.60	0.00	0.00	-0.02	0.02	21.26	-8
Mon. 3	3.68	-3.14	-3.14	1.20	4.60	0.00	0.00	-0.02	0.02	21.26	-8
Mon. 4	3.68	3.14	-3.14	1.20	4.60	0.00	0.00	-0.02	0.02	21.26	-8

2.3 ML-MCTDH Trees

Figures S6 to S8 show the Multilayer-MCTDH trees constructed to perform the wavepacket propagations necessary to compute OPA and ECD spectra. The numbers between the circles nodes represent Single Particle Functions (SPFs), whereas the numbers next to the rectangles represent the primitive functions for each vibrational mode. Modes carrying higher couplings and gradients a higher number of primitive functions and are grouped in higher branches in the ML-MCTDH tree.

For delocalized and localized hierarchy Hamiltonians, blocks become successively less relevant as the number of blocks increases, and thus their corresponding modes become more branched and have a lower number of primitive functions assigned for successive blocks. For the localized approach, modes corresponding to different fragments of the macrocycle were grouped so that the symmetry of the system is preserved in the propagation.

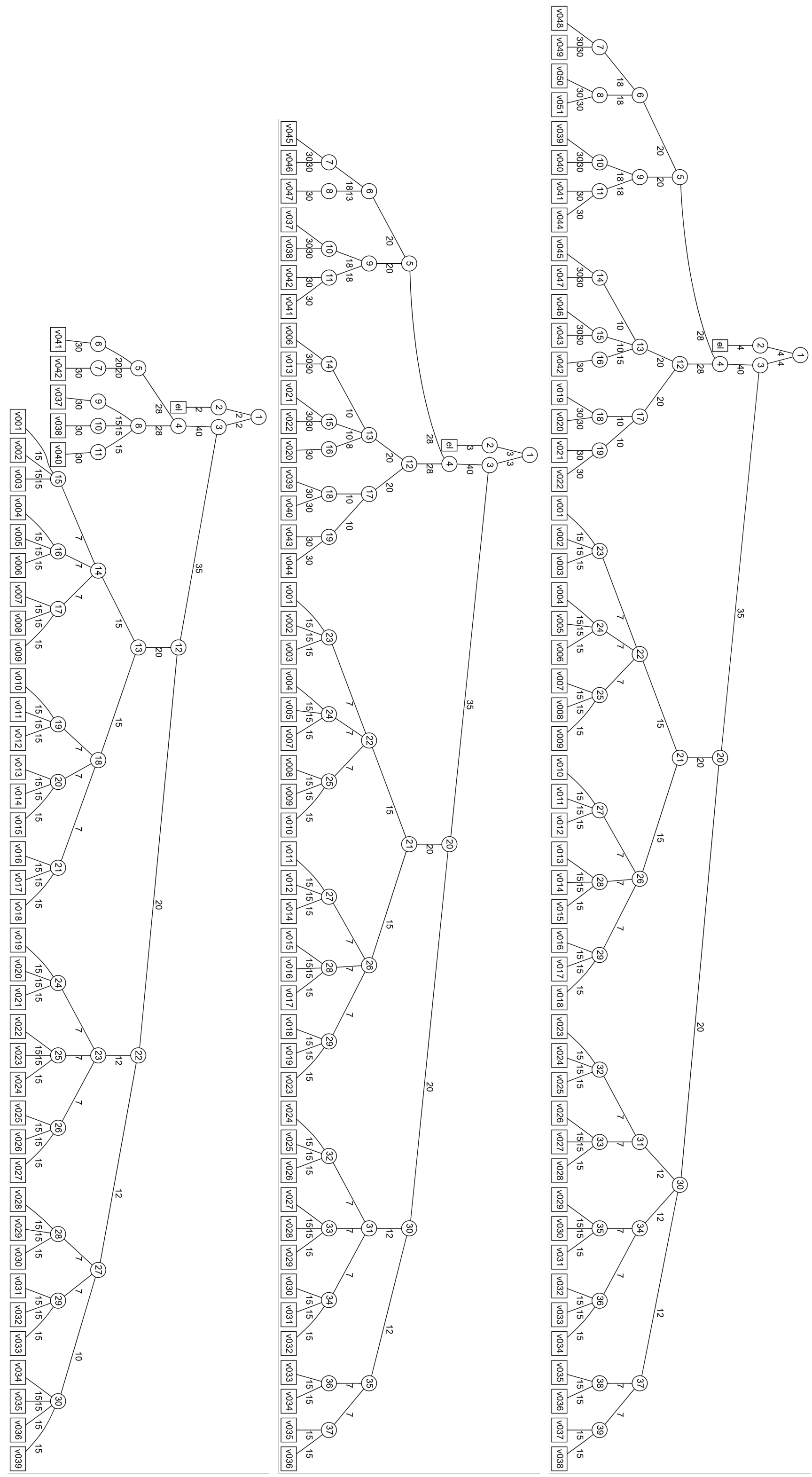

Figure S6: Multilayer-MCTDH trees for the delocalized St-LVC Hamiltonians for (P_2)-$\mathbf{DES}_2$ (left), (P_3)-$\mathbf{DES}_3$ (middle) and (P_4)-$\mathbf{DES}_4$ (right).

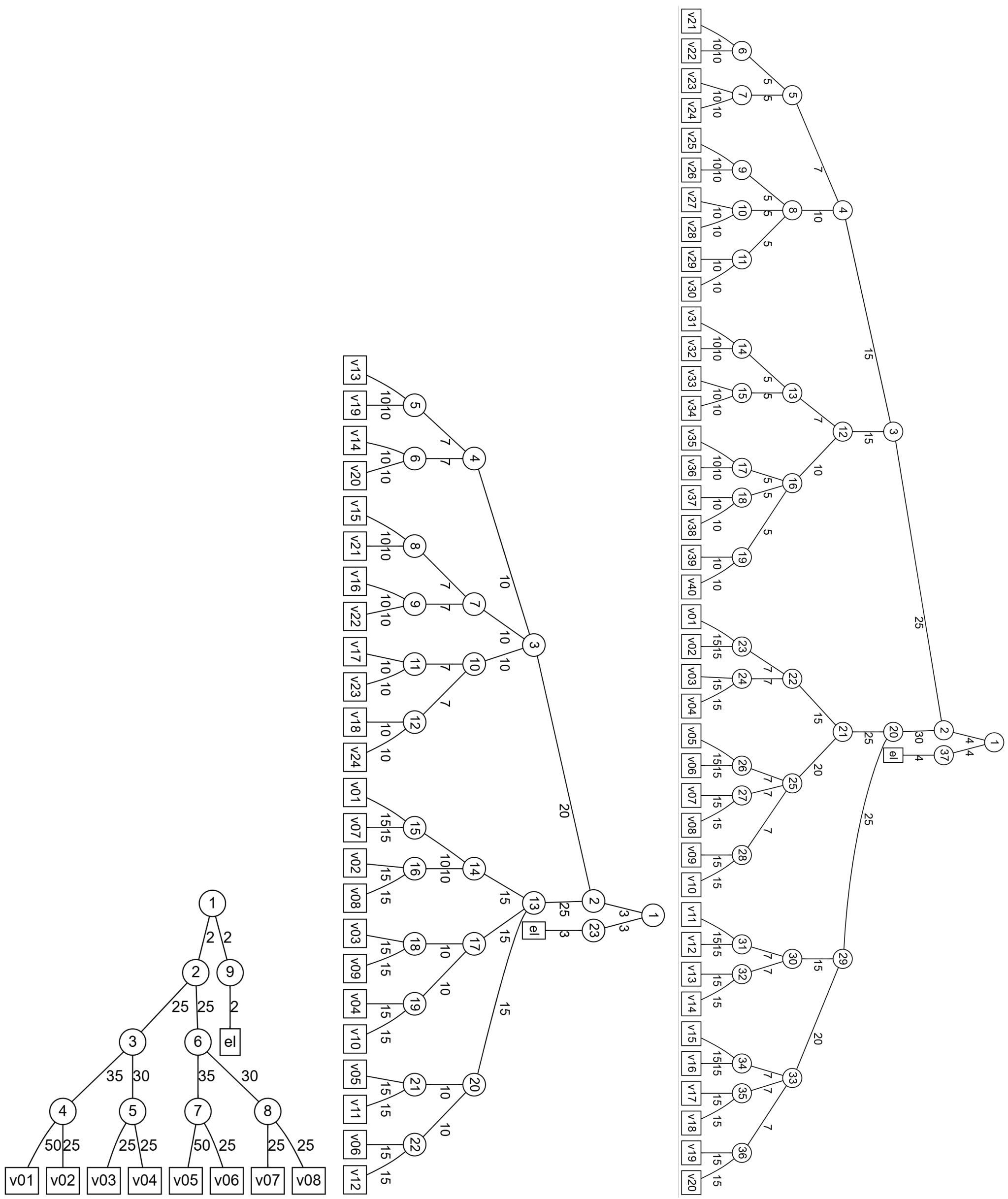

Figure S7: Multilayer-MCTDH trees for the delocalized Hierarchy Hamiltonians for (P_2)-$\mathbf{DES}_2$ (left), (P_3)-$\mathbf{DES}_3$ (middle) and (P_4)-$\mathbf{DES}_4$ (right).

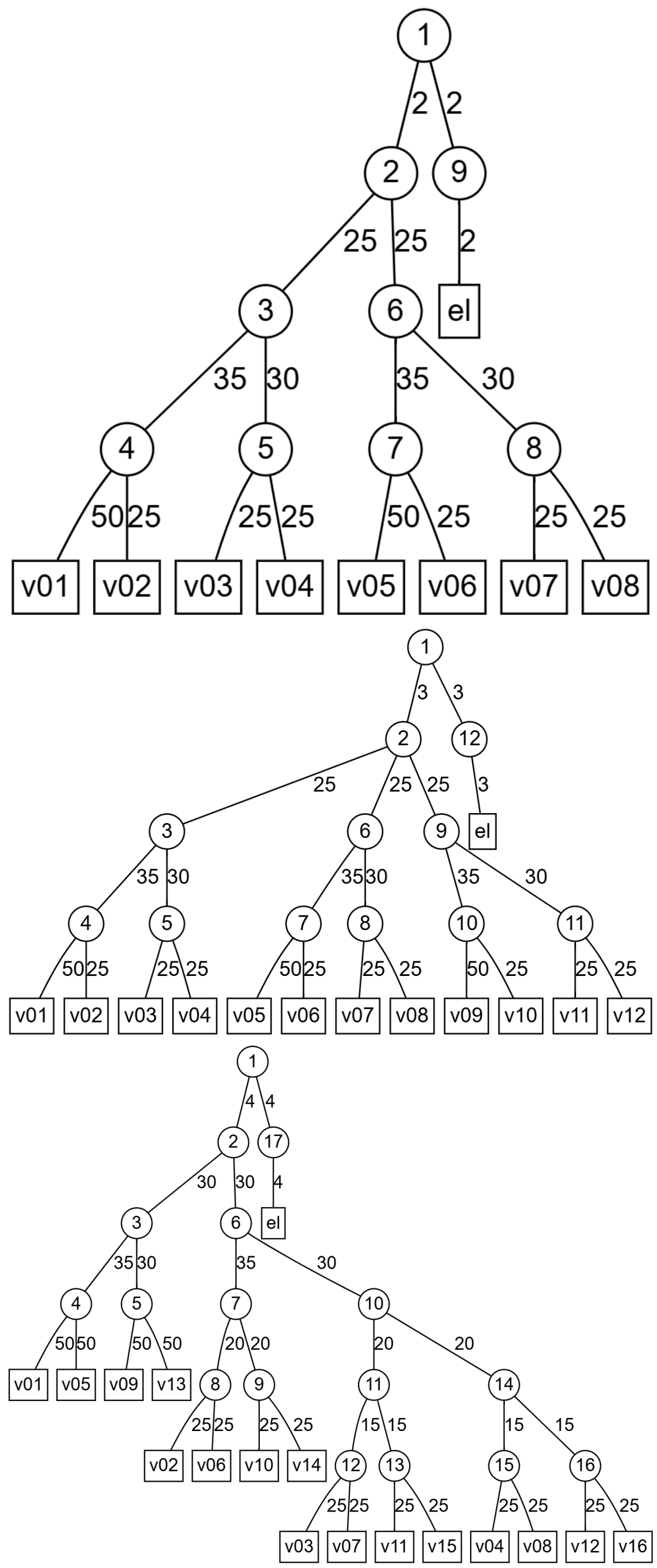

Figure S8: Multilayer-MCTDH trees for the localized Hierarchy Hamiltonians for (P_2)-$\mathbf{DES}_2$ (top), (P_3)-$\mathbf{DES}_3$ (middle) and (P_4)-$\mathbf{DES}_4$ (bottom).

3 Potential Energy Surfaces

3.1 (P_2)-DES$_2$

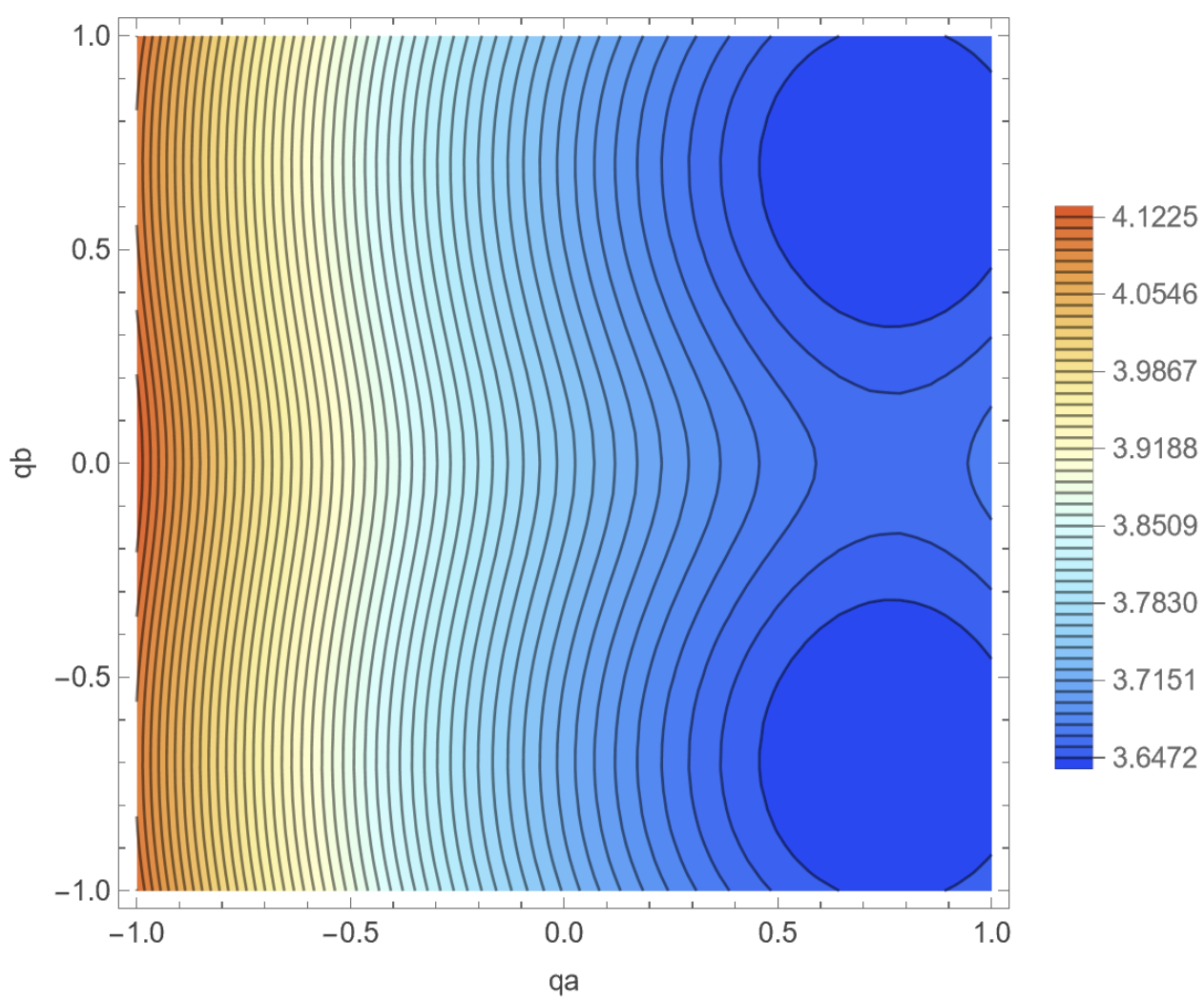

Figure S9: Contour plot of S_1 along the modes 223 ($q_{b(2)}$) and 224 ($q_{a(2)}$) for (P_2)-**DES**$_2$, expressed in dimensionless coordinates.

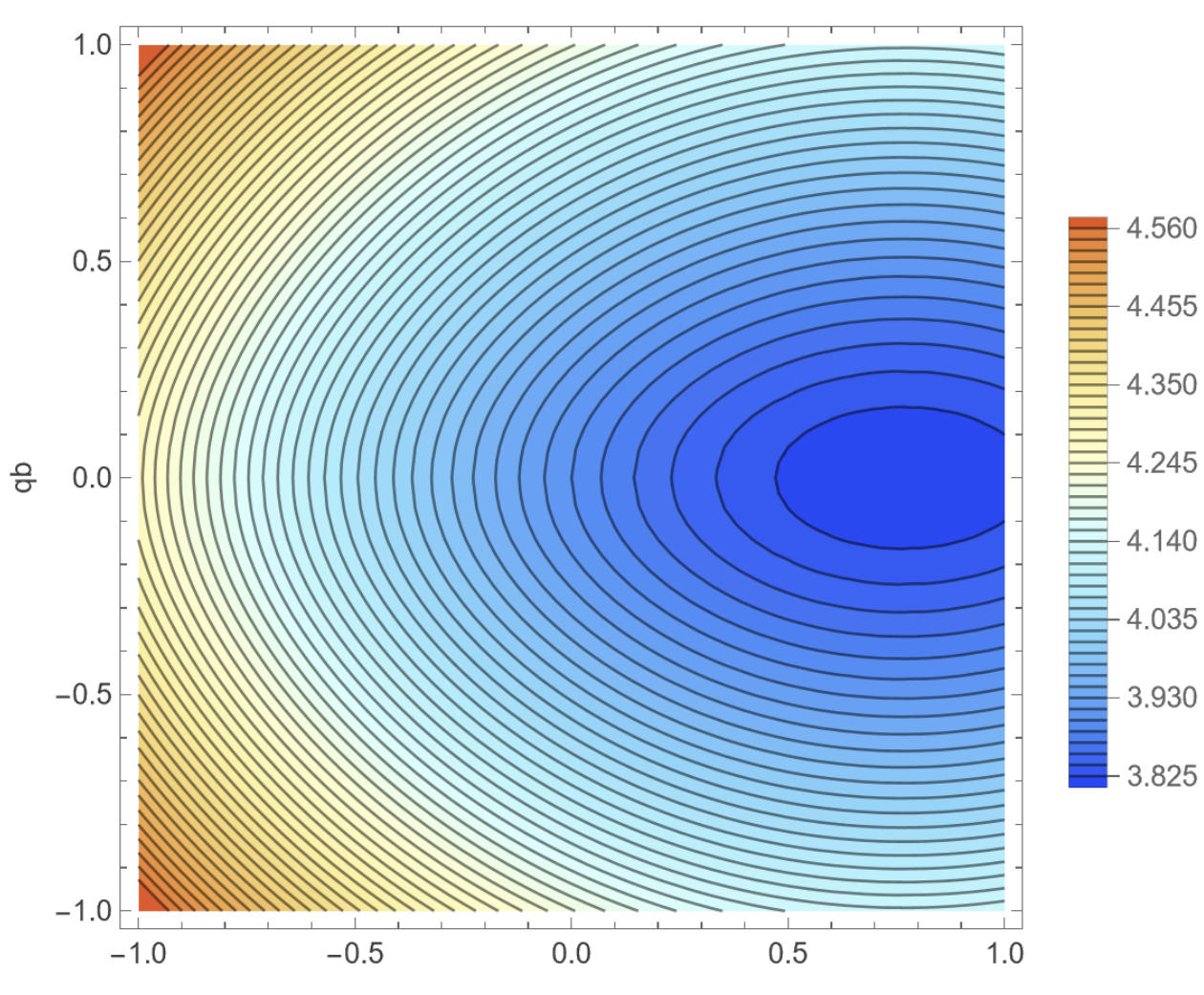

Figure S10: Contour plot of S_2 along the modes 223 ($q_{b(2)}$) and 224 ($q_{a(2)}$) for (P_2)-**DES**$_2$, expressed in dimensionless coordinates.

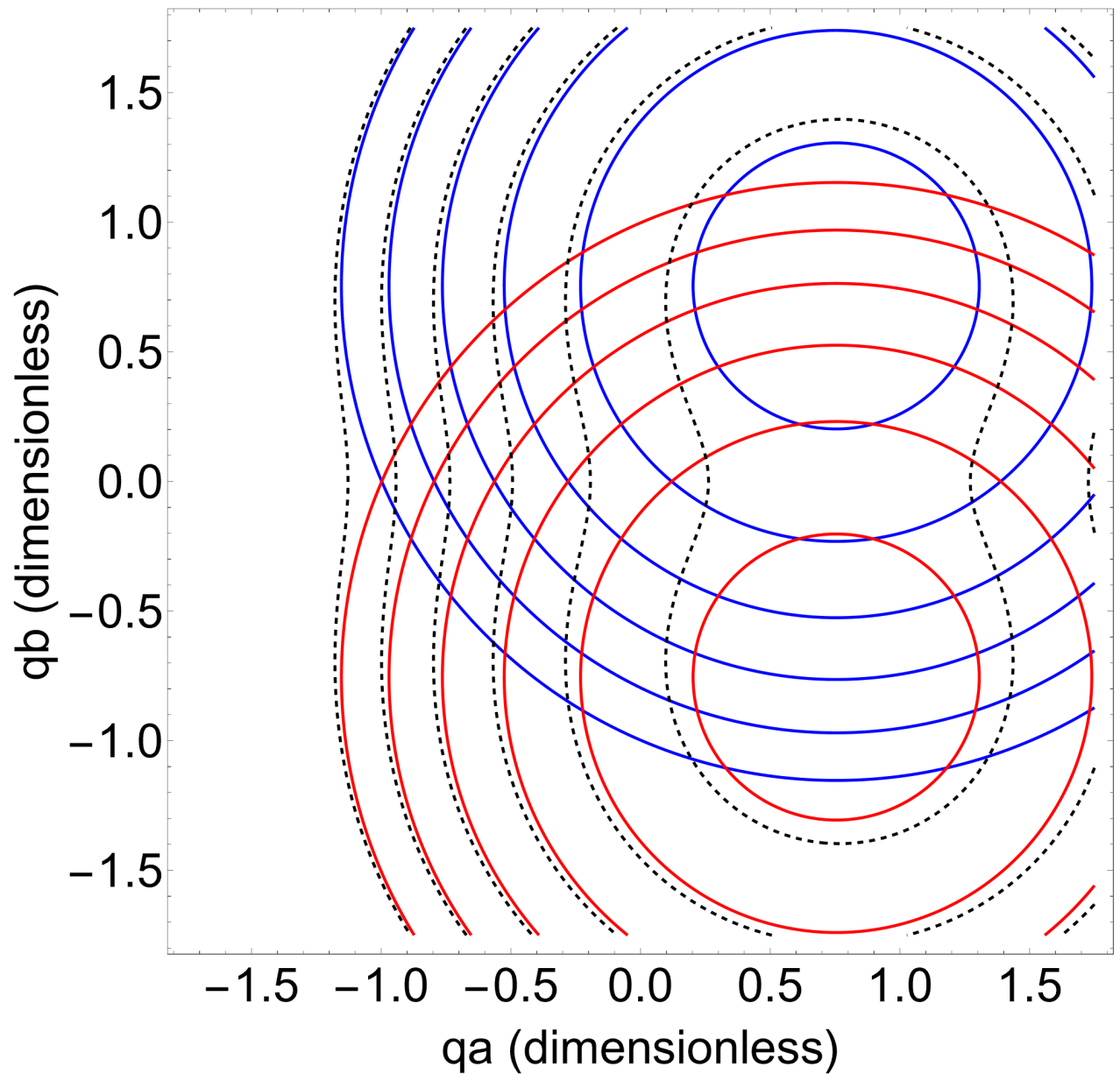

Figure S11: Contour plots for S_1 (dashed black lines) and localized states (full color lines) along modes 223 ($q_{b(2)}$) and 224 ($q_{a(2)}$) for (P_2)-**DES**$_2$, expressed in dimensionless coordinates.

3.1.1 On the absence of Conical Intersections in (P_2)-DES$_2$ with our model

Diagonalization of the delocalized diabatic matrix $\mathbf{H}^{vib,D}_{dia(2)}$ yields the Potential Energy Surfaces for adiabatic states S_1 and S_2 of (P_2)-**DES**$_2$ as the diagonal terms of the adiabatic matrix $\mathbf{H}^{vib}_{adia(2)}$:

$$\mathbf{H}^{vib}_{adia(2)} = \begin{pmatrix} \Upsilon - \sqrt{\left(\frac{\lambda_{(2)}}{\sqrt{2}} q_{b(2)}\right)^2 + \beta^2} & 0 \\ 0 & \Upsilon + \sqrt{\left(\frac{\lambda_{(2)}}{\sqrt{2}} q_{b(2)}\right)^2 + \beta^2} \end{pmatrix} \tag{18}$$

Where $\Upsilon = E_{0(2)} + \frac{\lambda_{(2)}}{\sqrt{2}} q_{a(2)} + \frac{\omega}{2}(q^2_{a(2)} + q^2_{b(2)})$. For a CoI to exist, the condition

$$-\sqrt{\left(\frac{\lambda_{(2)}}{\sqrt{2}} q_{b(2)}\right)^2 + \beta^2} = \sqrt{\left(\frac{\lambda_{(2)}}{\sqrt{2}} q_{b(2)}\right)^2 + \beta^2} \tag{19}$$

needs to be fulfilled. This implies that $\left(\frac{\lambda_{(2)}}{\sqrt{2}} q_{b(2)}\right)^2 + \beta^2 = 0$, and since all values are real, it is required that both β and $\frac{\lambda_{(2)}}{\sqrt{2}} q_{b(2)}$ are zero. Given that $\beta \neq 0$, no CoI

can exist for (P_2)-$\mathbf{DES}_2$ within our model.

3.2 (P_3)-$\mathbf{DES}_3$

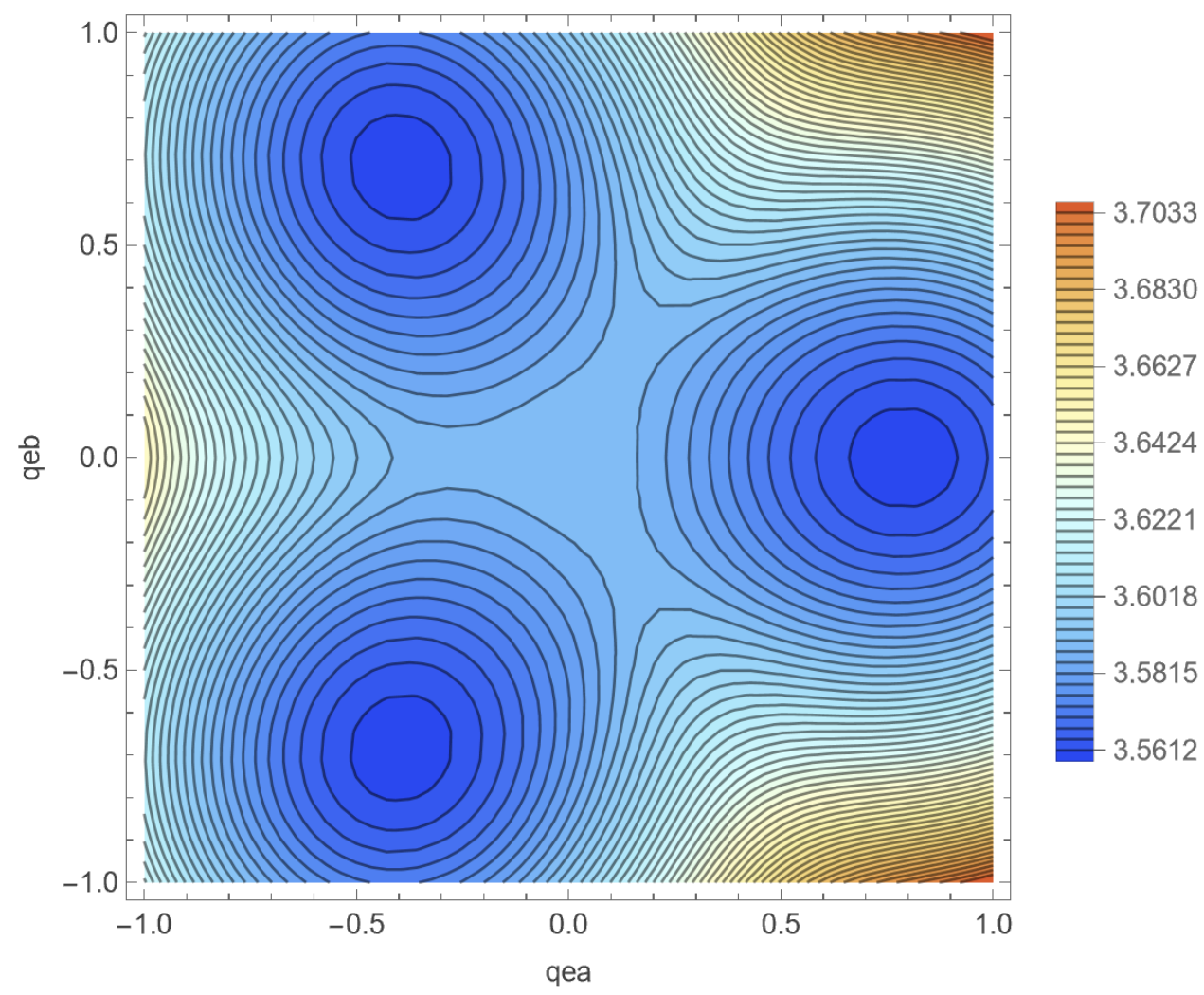

Figure S12: Contour plot of S_1 along the modes 337 ($q_{e_a(3)}$) and 338 ($q_{e_b(3)}$) for (P_3)-$\mathbf{DES}_3$, expressed in dimensionless coordinates.

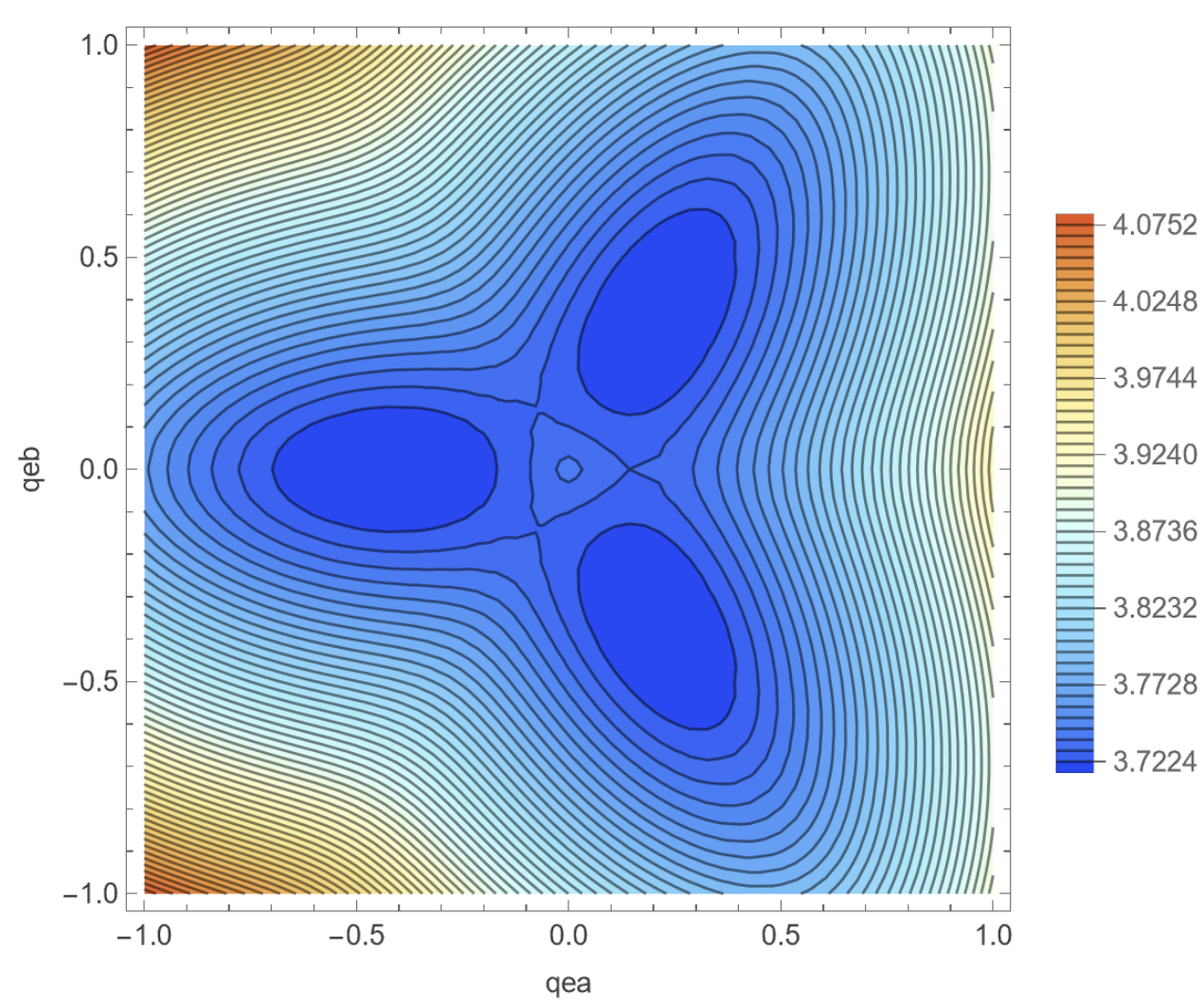

Figure S13: Contour plot of S_2 along the modes 337 ($q_{e_a(3)}$) and 338 ($q_{e_b(3)}$) for (P_3)-$\mathbf{DES}_3$, expressed in dimensionless coordinates.

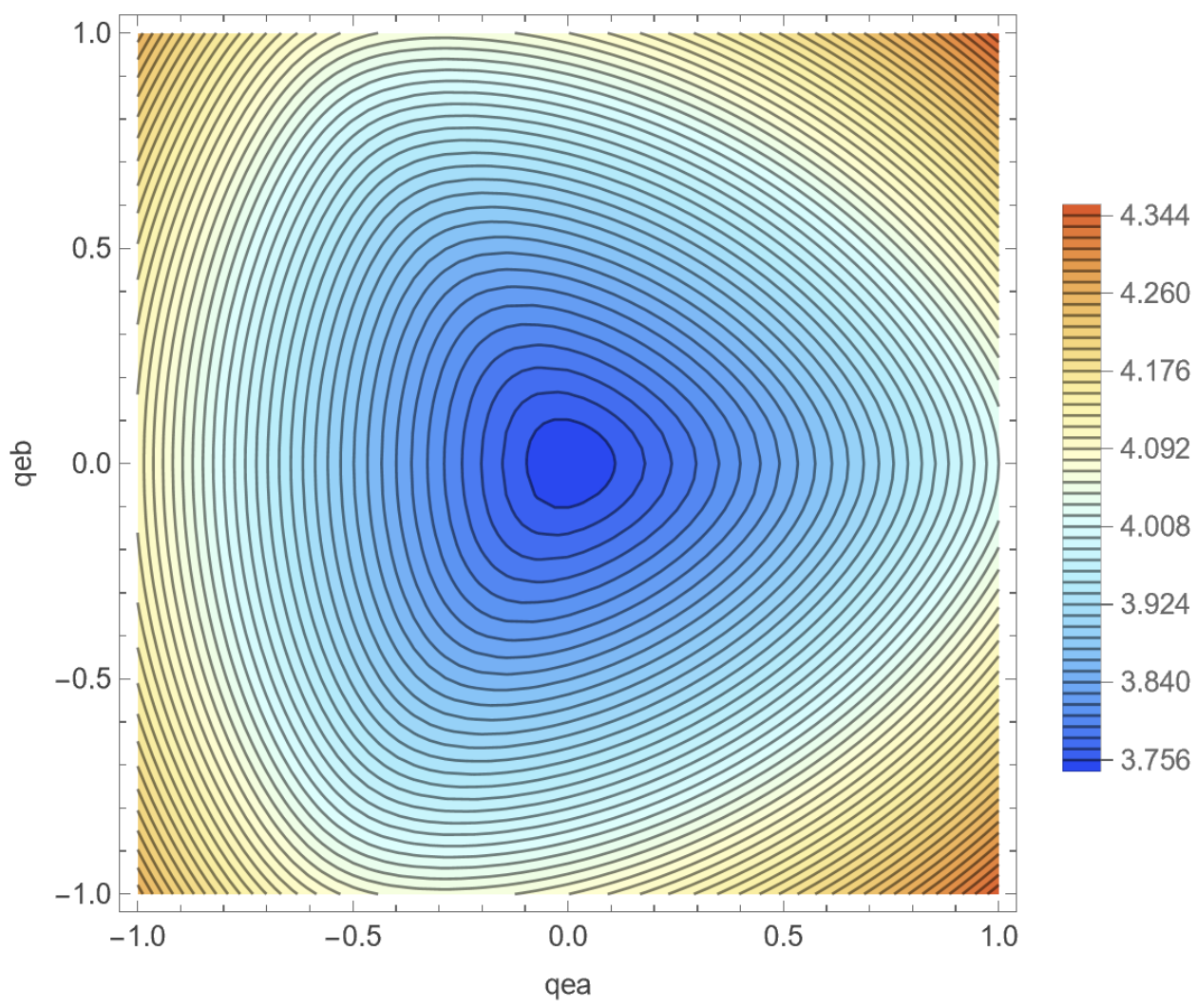

Figure S14: Contour plot of S_3 along the modes 337 ($q_{e_a(3)}$) and 338 ($q_{e_b(3)}$) for (P_3)-**DES**$_3$, expressed in dimensionless coordinates.

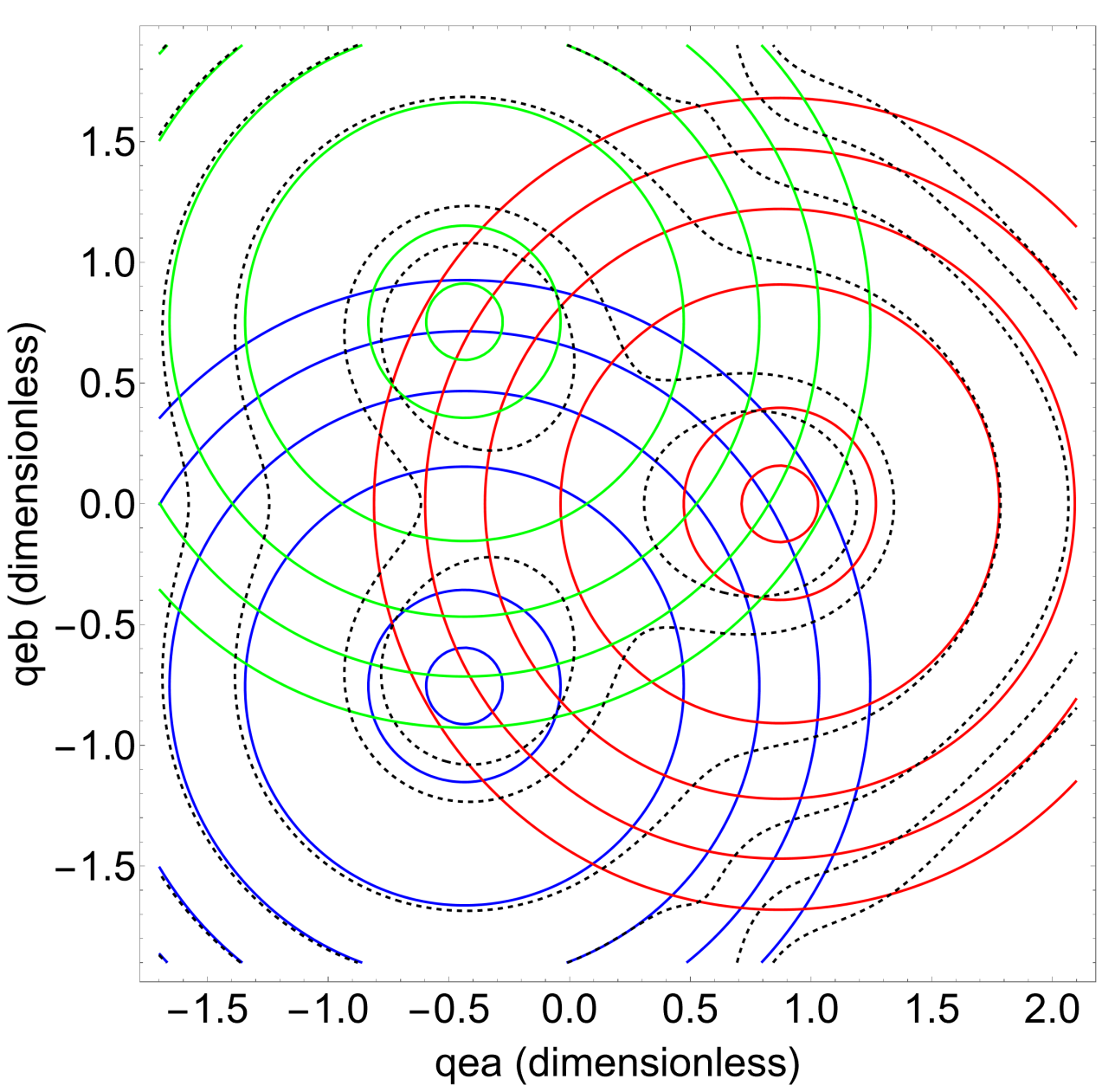

Figure S15: Contour plots for S_1 (dashed black lines) and localized states (full color lines) along modes 337 ($q_{e_a(3)}$) and 338 ($q_{e_a(3)}$) for (P_3)-**DES**$_3$, expressed in dimensionless coordinates.

3.3 (P_4)-DES$_4$

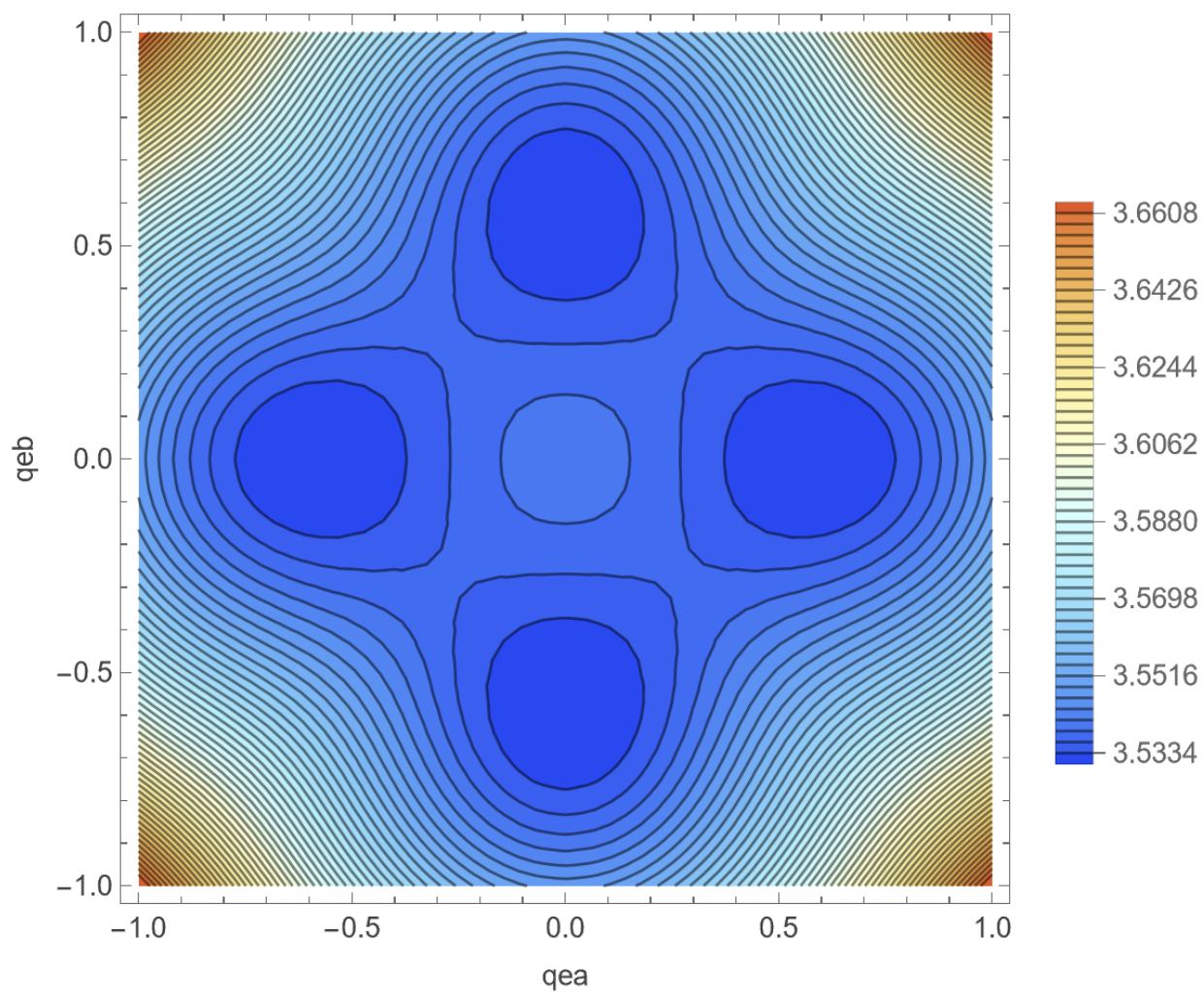

Figure S16: Contour plot of S_1 along the modes 452 ($q_{e_a(4)}$) and 453 ($q_{e_b(34)}$) for (P_4)-**DES**$_4$, expressed in dimensionless coordinates.

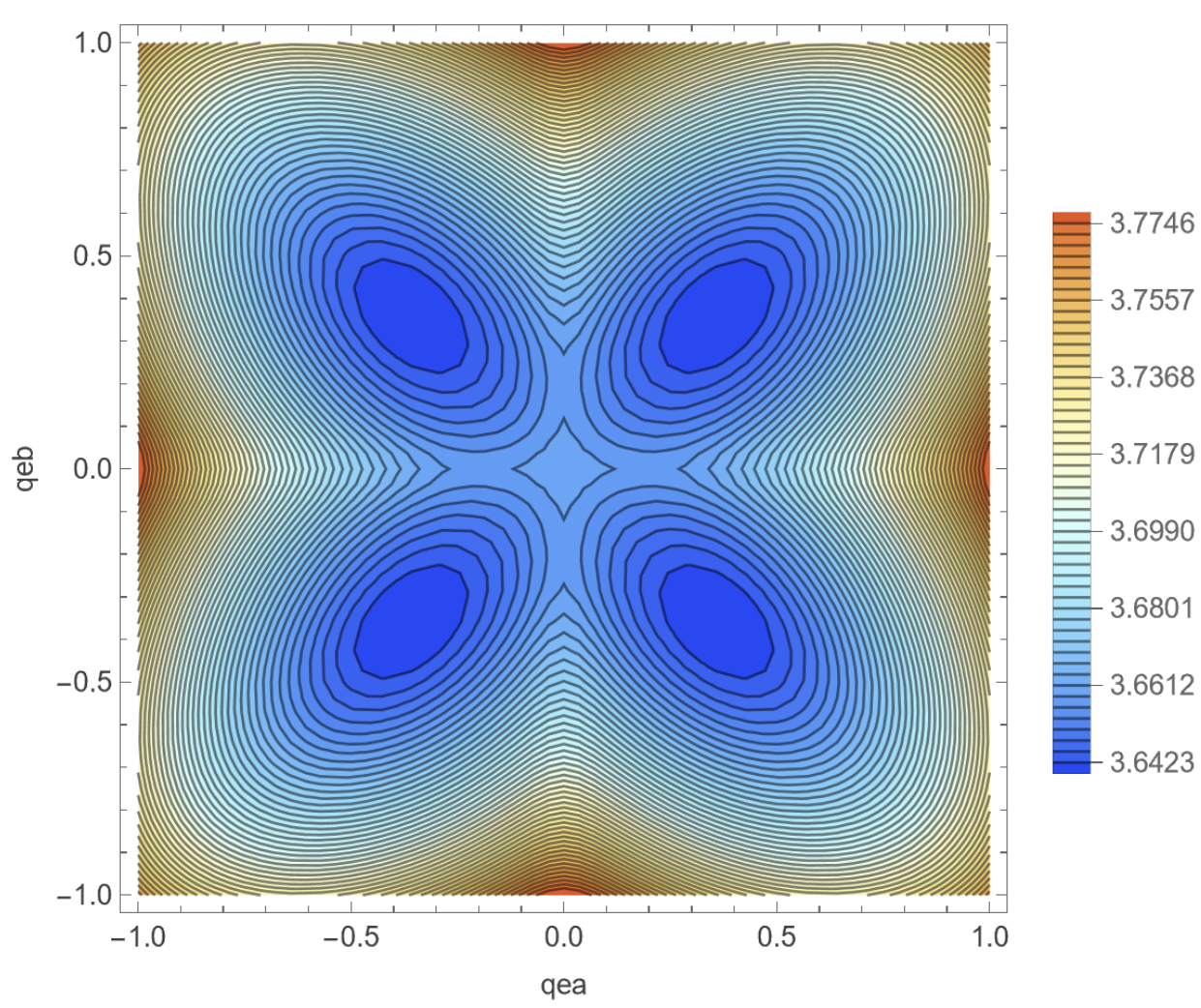

Figure S17: Contour plot of S_2 along the modes 452 ($q_{e_a(4)}$) and 453 ($q_{e_a(4)}$) for (P_4)-**DES**$_4$, expressed in dimensionless coordinates.

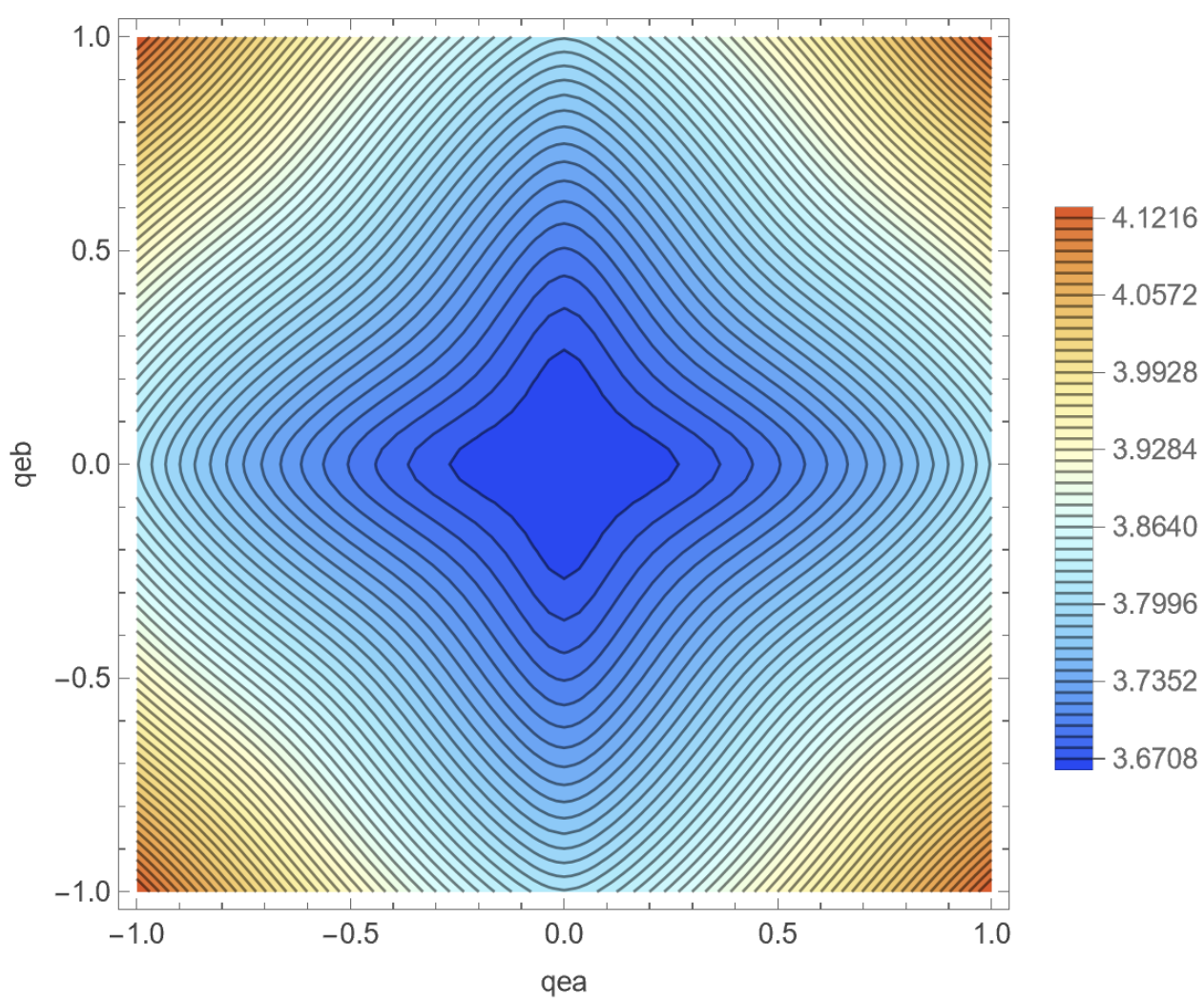

Figure S18: Contour plot of S_3 along the modes 452 ($q_{e_a(4)}$) and 453 ($q_{e_a(4)}$) for (P_4)-**DES**$_4$, expressed in dimensionless coordinates.

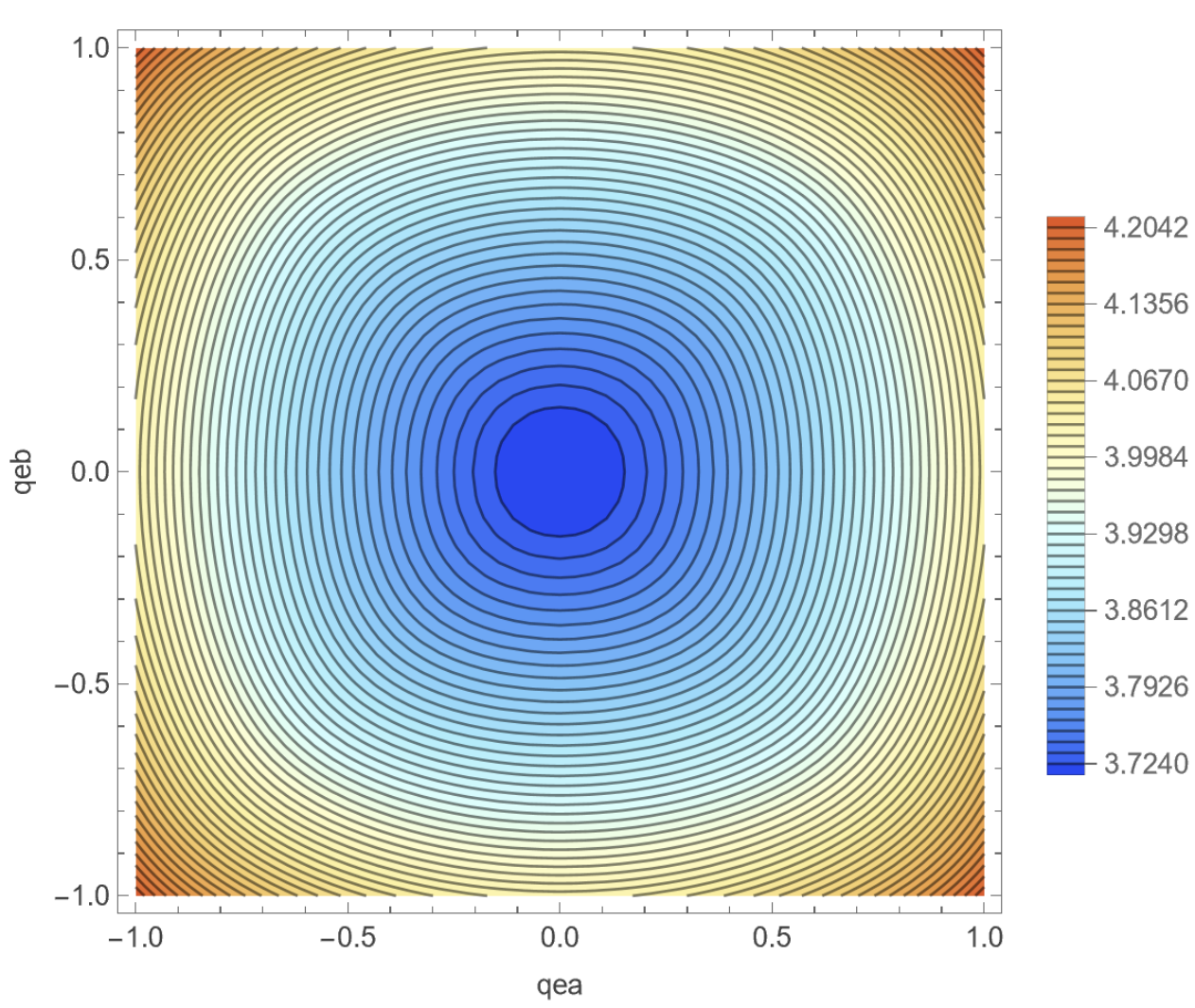

Figure S19: Contour plot of S_4 along the modes 452 ($q_{e_a(4)}$) and 453 ($q_{e_b(4)}$) for (P_4)-**DES**$_4$, expressed in dimensionless coordinates.

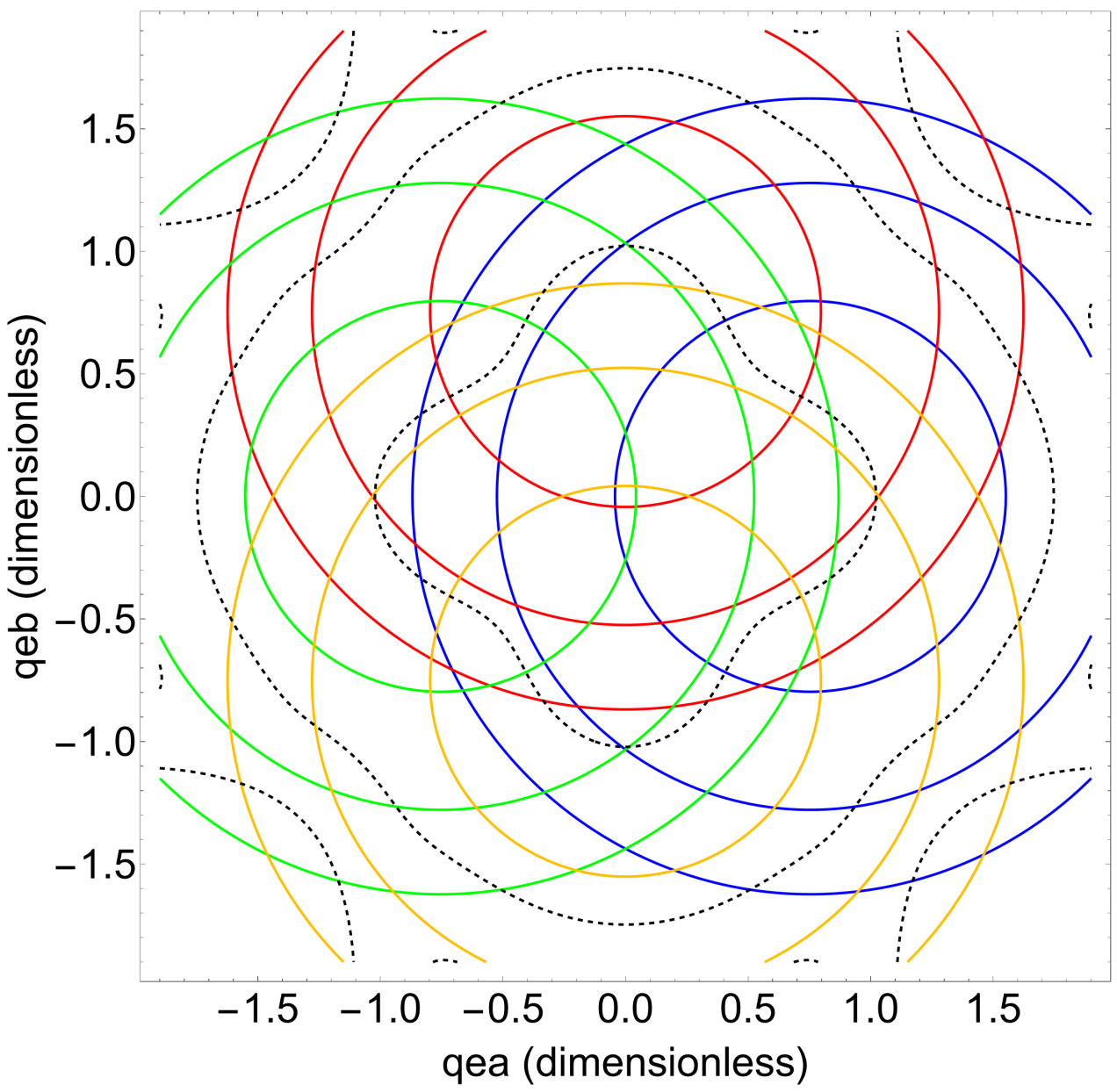

Figure S20: Contour plots for S_1 (dashed black lines) and localized states (full color lines) along the modes 452 ($q_{e_a(4)}$) and 453 ($q_{e_b(4)}$) for (P_4)-**DES**$_4$, expressed in dimensionless coordinates.

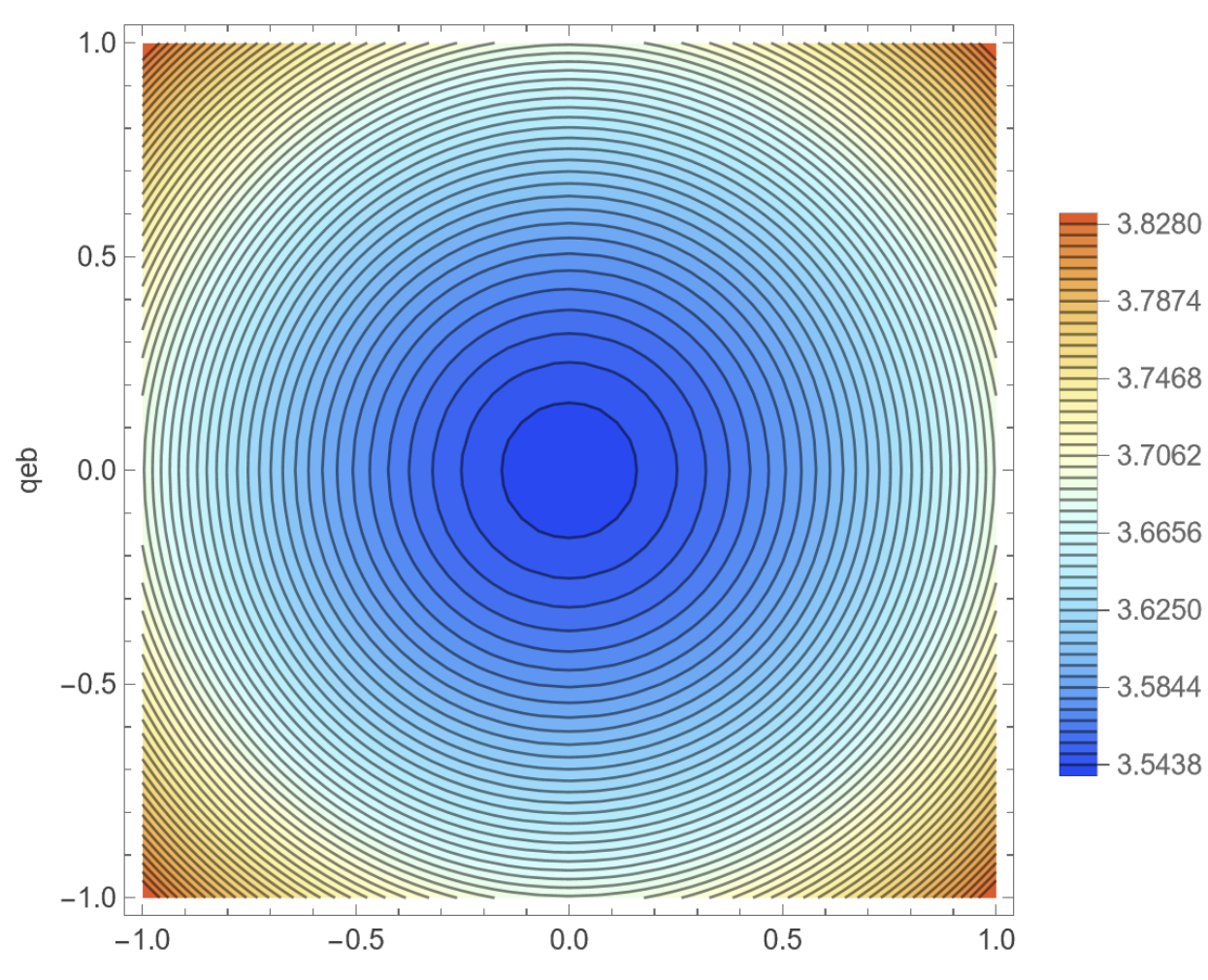

Figure S21: Contour plot of S_1 along the modes 452 ($q_{e_a(4)}$) and 453 ($q_{e_b(4)}$) for (P_4)-**DES**$_4$ setting pJT couplings and all interstate couplings to zero, expressed in dimensionless coordinates.

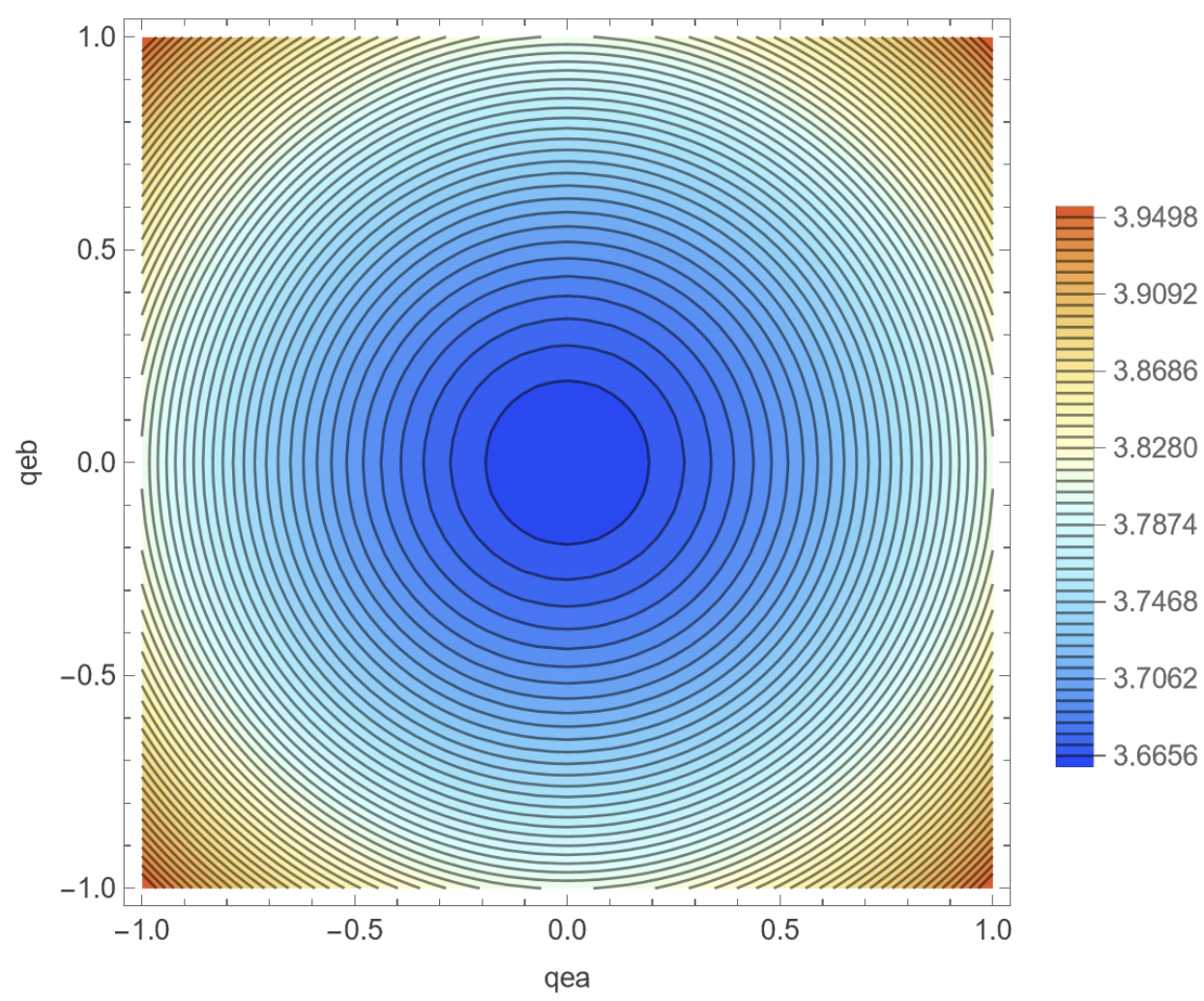

Figure S22: Contour plot of S_2 along the modes 452 ($q_{e_a(4)}$) and 453 ($q_{e_b(4)}$) for (P_4)-$\mathbf{DES}_4$ setting pJT couplings and all interstate couplings to zero, expressed in dimensionless coordinates.

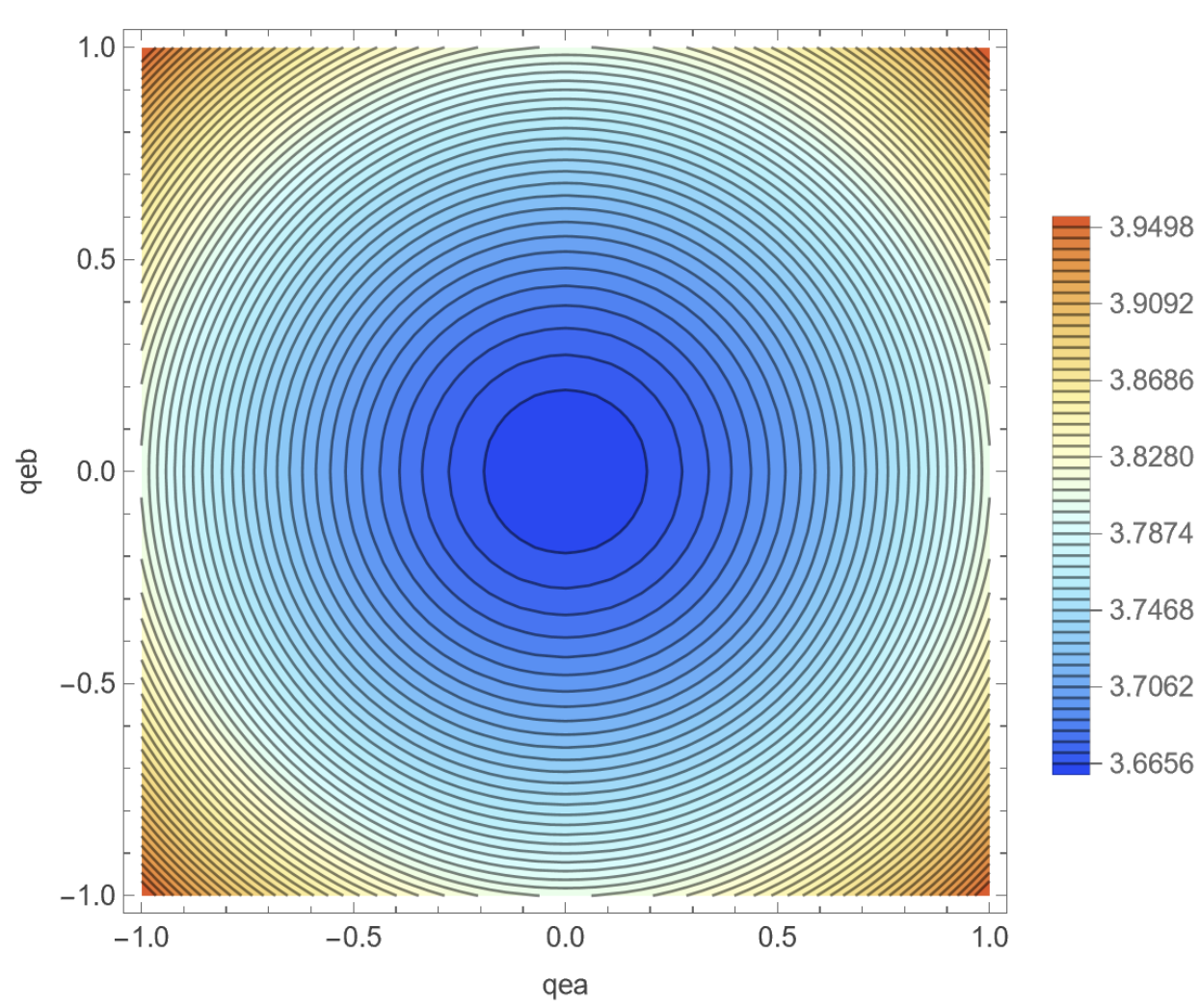

Figure S23: Contour plot of S_3 along the modes 452 ($q_{e_a(4)}$) and 453 ($q_{e_b(4)}$) for (P_4)-$\mathbf{DES}_4$ setting pJT couplings and all interstate couplings to zero, expressed in dimensionless coordinates.

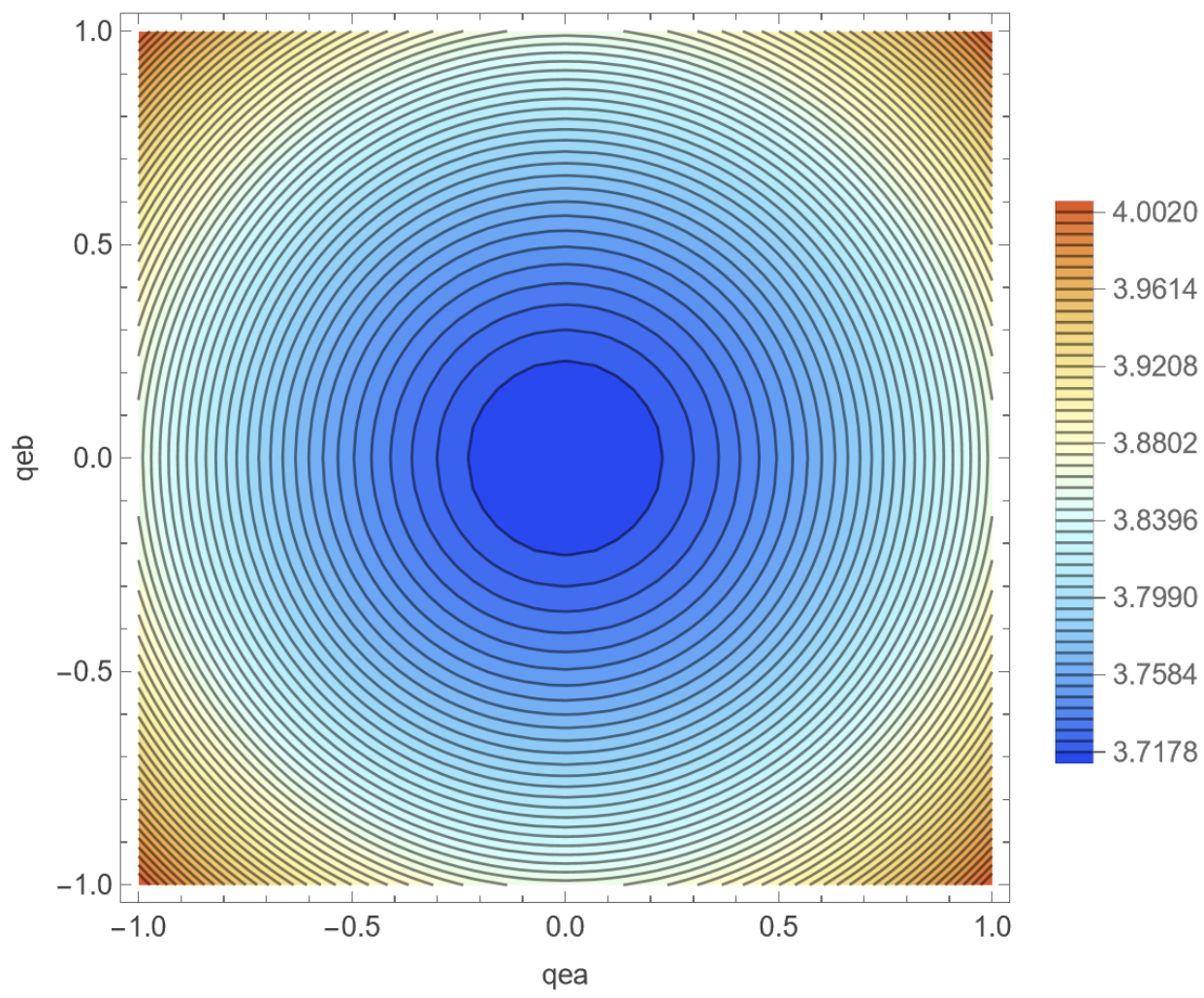

Figure S24: Contour plot of S_4 along the modes 452 ($q_{e_a(4)}$) and 453 ($q_{e_b(4)}$) for (P_4)-$\mathbf{DES}_4$ setting pJT couplings and all interstate couplings to zero, expressed in dimensionless coordinates.

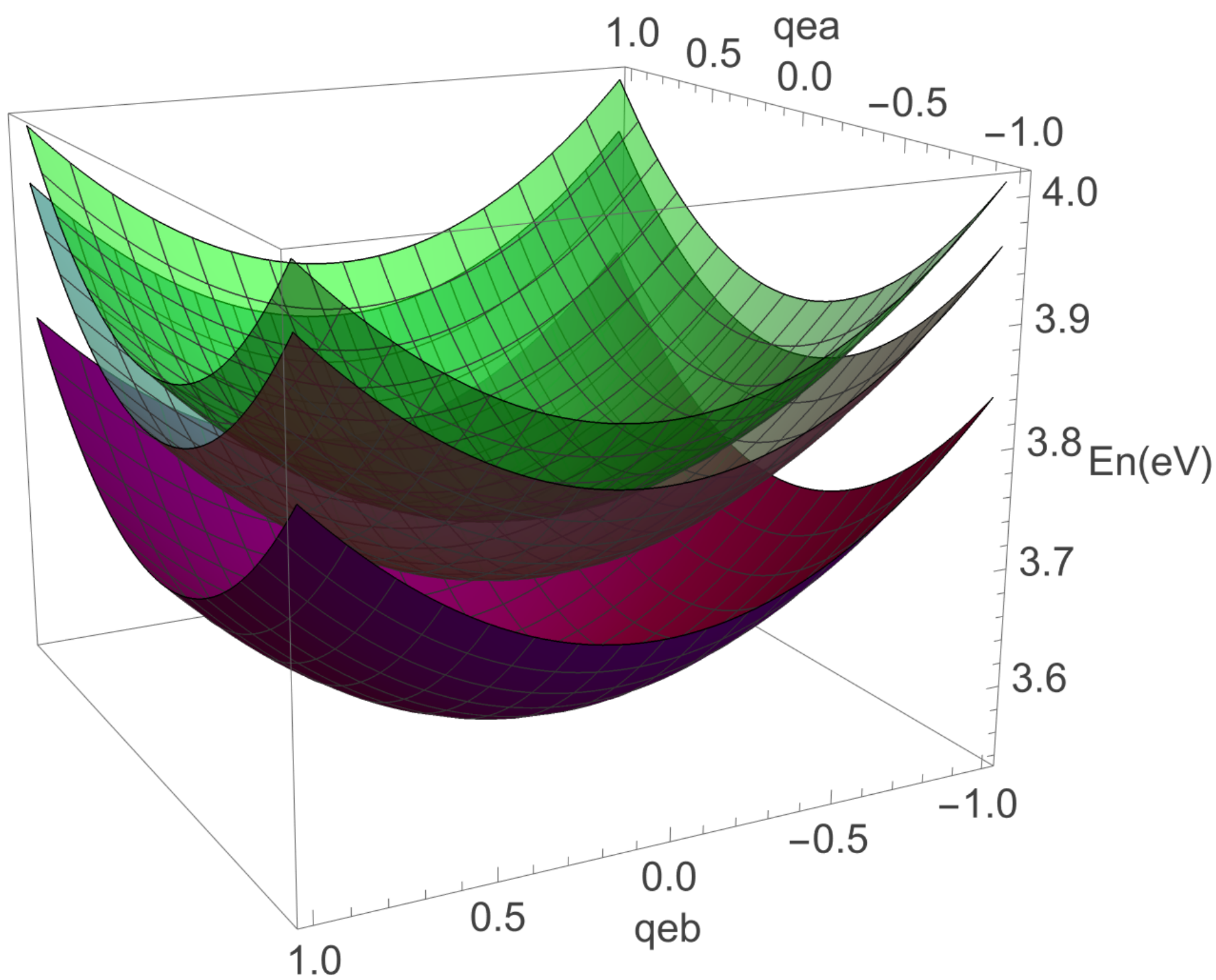

Figure S25: Contour plot of S_1 to S_4 along the modes 452 ($q_{e_a(4)}$) and 453 ($q_{e_b(4)}$) for (P_4)-$\mathbf{DES}_4$ setting pJT couplings and all interstate couplings to zero, expressed in dimensionless coordinates.

4 A more Extended Discussion on (P_3)-DES$_3$ LVC and Spectra

Table S9 shows the data for the lowest-lying in energy excited states for (P_3)-**DES**$_3$. The first excited state, S$_1$, is non-dark and counts with a high Rotatory Strength ($\approx$ 3858 10^{-40} cgs). The rotatory strength of the S$_2$/S$_3$ degenerate pair is about half of this value, whereas their dipole strengths are very high at 25.10 a.u; therefore, these states are expected to play a prominent role in the low-energy region for both OPA and ECD spectra. The S$_4$/S$_5$ degenerate pair and S6 show both extremely low dipole and Rotatory Strengths, lying about 0.37 eV higher in energy than the S$_2$/S$_3$ pair. This energy gap forbids these states from coupling, indicating that it is only necessary to include S$_1$, S$_2$ and S$_3$ in the Hamiltonian.

Table S9: Vertical Energies (VE), dipole strength (Dip. Str. in a.u.), rotatory strength (in velocity gauge (R_{vel}) and angle between electric and magnetic transition dipole moments (θ_{EM} for the six lowest energy adiabatic states of (P_3)-**DES**$_3$. CAM-B3LYP/6-31G(d) level of theory.

System	State	Symm.	VE (eV)	Dip. Str (a.u.)	R_{vel} (10^{-40} cgs)	θ_{EM}
(P_3)-**DES**$_3$	S$_1$	A$_2$	3.58	2.10	3858.02	0.00
	S$_2$	E	3.74	25.10	-2045.47	180.00
	S$_3$	E	3.74	25.10	-2045.47	180.00
	S$_4$	E	4.11	0.01	5.50	0.00
	S$_5$	E	4.11	0.01	5.50	0.00
	S$_6$	A$_1$	4.11	0.00	0.00	-

According to the ideal delocalized Hamiltonian model (Eq. **??**), the energy gap between S$_1$ and the degenerate pair is 3β. Using the calculated exciton coupling, $\beta = -0.059$ eV (Table 1), the predicted energy gap is $\approx$ 0.18 eV, a value very similar to the computed gap between S$_1$ and S$_2$ (0.16 eV). The origin of this small difference could be due to the interaction of the S$_2$/S$_3$ pair with the S$_4$/S$_5$ degenerate pair, which is not taken into account in the idealized model.

Table S10 features the three most prominent modes for the St-LVC Hamiltonian

of (P_3)-$\mathbf{DES}_3$, which account for $\approx$ 80 % of the total vibronic coupling. Attending to the ideal delocalized Hamiltonian (Equation **??**), the gradients due to a totally-symmetric mode (Mode 339) should be equal for all 3 states, whereas a pair of degenerate E symmetry modes account for a part of the gradients of S_2 and S_3 (Mode 337) and for the coupling with respect to S_1 and between S_2 and S_3 (Modes 337 and 338). The calculated magnitudes for the couplings are also in very good agreement with the idealized model: for instance, both the computed gradients in S_2 and S_3 along one of the degenerate modes, and the coupling between S_2 and S_3, are predicted to be $\frac{\sqrt{3}}{\sqrt{6}} = 0.71$ times the value for the rest of the gradients and couplings. Taking into account that these values are 0.13-0.14 and 0.18-0.19, respectively, the factor for the calculated data ranges between 0.68 and 0.78.

Table S10: LVC parameters for the most relevant normal modes of (P_3)-$\mathbf{DES}_3$. All λ in eV. CAM-B3LYP/6-31G(d) level of theory.

Modes	Symm.	Freq (cm^{-1})	λ_{11}	λ_{22}	λ_{33}	λ_{12}	λ_{13}	λ_{23}
337	E	2401.00	0.00	0.13	-0.14	0.00	-0.19	0.00
338	E	2401.00	0.00	0.00	0.00	-0.19	0.00	0.13
339	A_1	2401.00	-0.18	-0.18	-0.18	0.00	0.00	0.00
Norm 3 Modes			0.18	0.22	0.23	0.19	0.19	0.13
Norm All Modes			0.23	0.27	0.27	0.22	0.22	0.16

4.0.1 OPA and ECD Spectra.

Figure S26 compares experimental, delocalized spectra computed through the St-LVC methodology, and localized spectra obtained through the FrD-LVC model. Both approaches agree correctly with each other and with the experimental data for OPA and ECD, being able to reproduce the peaks present at both spectra at $\approx$ 3.30 and 3.60 eV. To gain insight into the influence of non-adiabatic effects, Figure S26 compares the St-LVC model with FC|VG calculated spectra as well. This model practically accounts only for S_2 and S_3 due to the dipole strength of

S_1 being comparatively much lower and agrees much poorer with the experimental data than the LVC model: in general, the spectra are blue-shifted, and specifically, in the OPA spectrum, the intensities of the two main peaks are reversed and the shoulder at higher energies is not well-reproduced. In ECD, the position of both peaks is not correctly predicted by FC|VG, with the negative band being overestimated and both bands being too close in energy.

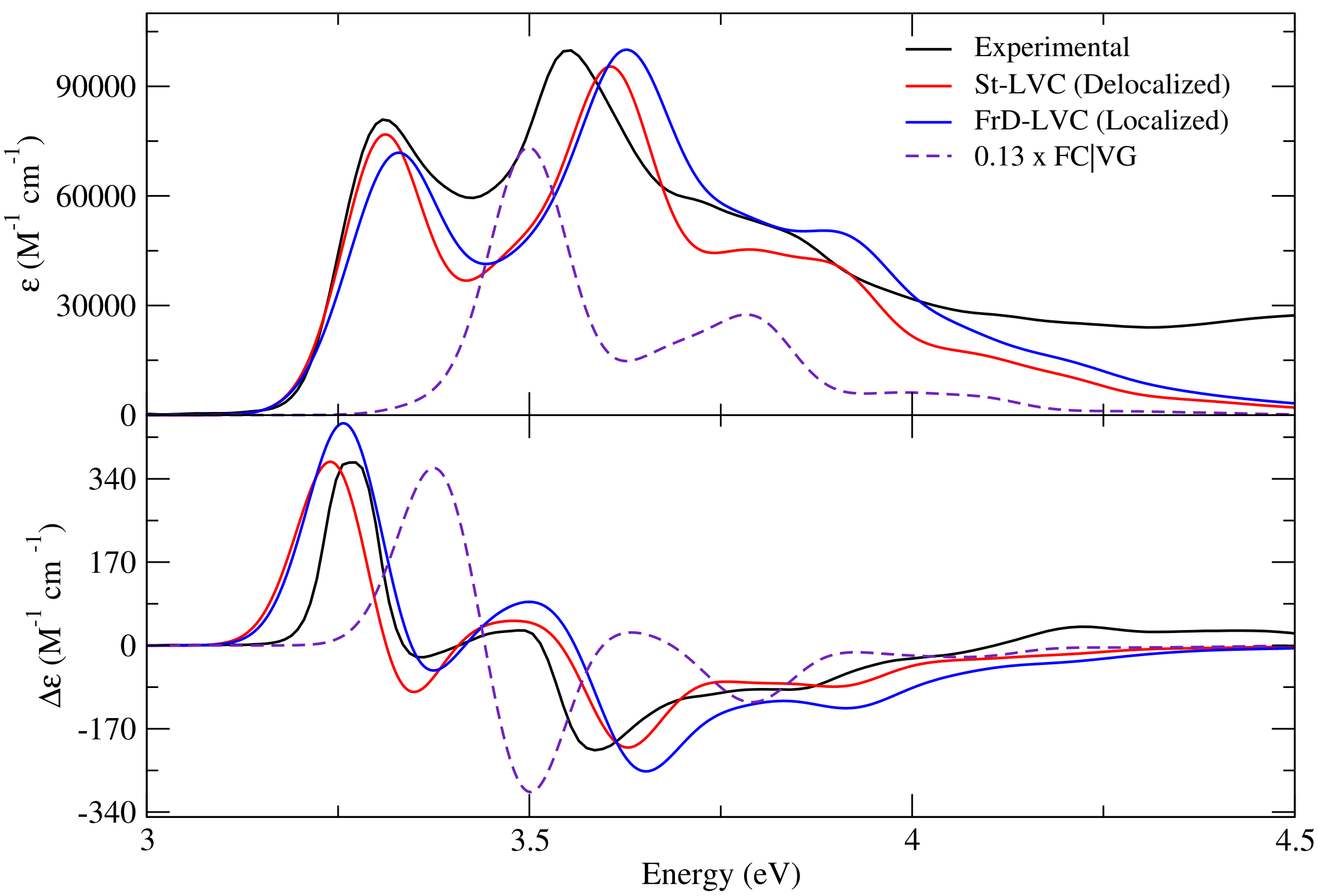

Figure S26: Comparison of computed and experimental ABS and ECD spectra for (P_3)-$\mathbf{DES_3}$. Computed spectra were red-shifted by 0.105 eV, and a Gaussian broadening with HWHM=0.064 eV was set for both ABS and ECD. LVC OPA and ECD spectra were scaled by 0.35 and 0.26, respectively. CAM-B3LYP/6-31G(d) level of theory.

5 Extended LVC Parameters of (P_4)-DES$_4$

Table S11: Modes with minor contributions to the LVC Hamiltonian of (P_4)-**DES**$_4$. All λ in eV. CAM-B3LYP/6-31G(d) level of theory.

Modes	Symm.	Freq (cm^{-1})	λ_{11}	λ_{22}	λ_{33}	λ_{44}	λ_{12}	λ_{13}	λ_{14}	λ_{23}	λ_{24}	λ_{34}
432	B2	1700.54	0.00	0.08	-0.08	0.00	0.00	0.00	-0.08	0.00	0.00	0.00
433	E	1700.61	0.00	0.00	0.00	0.00	-0.05	-0.03	0.00	0.00	0.05	-0.03
434	E	1700.61	0.00	0.00	0.00	0.00	-0.03	0.05	0.00	0.00	0.03	0.05
438	A1	1701.66	-0.06	-0.05	-0.05	-0.05	0.00	0.00	0.00	0.00	0.00	0.00
443	A1	1702.18	-0.06	-0.05	0.06	-0.06	0.00	0.00	0.00	0.00	0.00	0.00

6 Dynamics of the Diabatic Electronic Populations for all Species

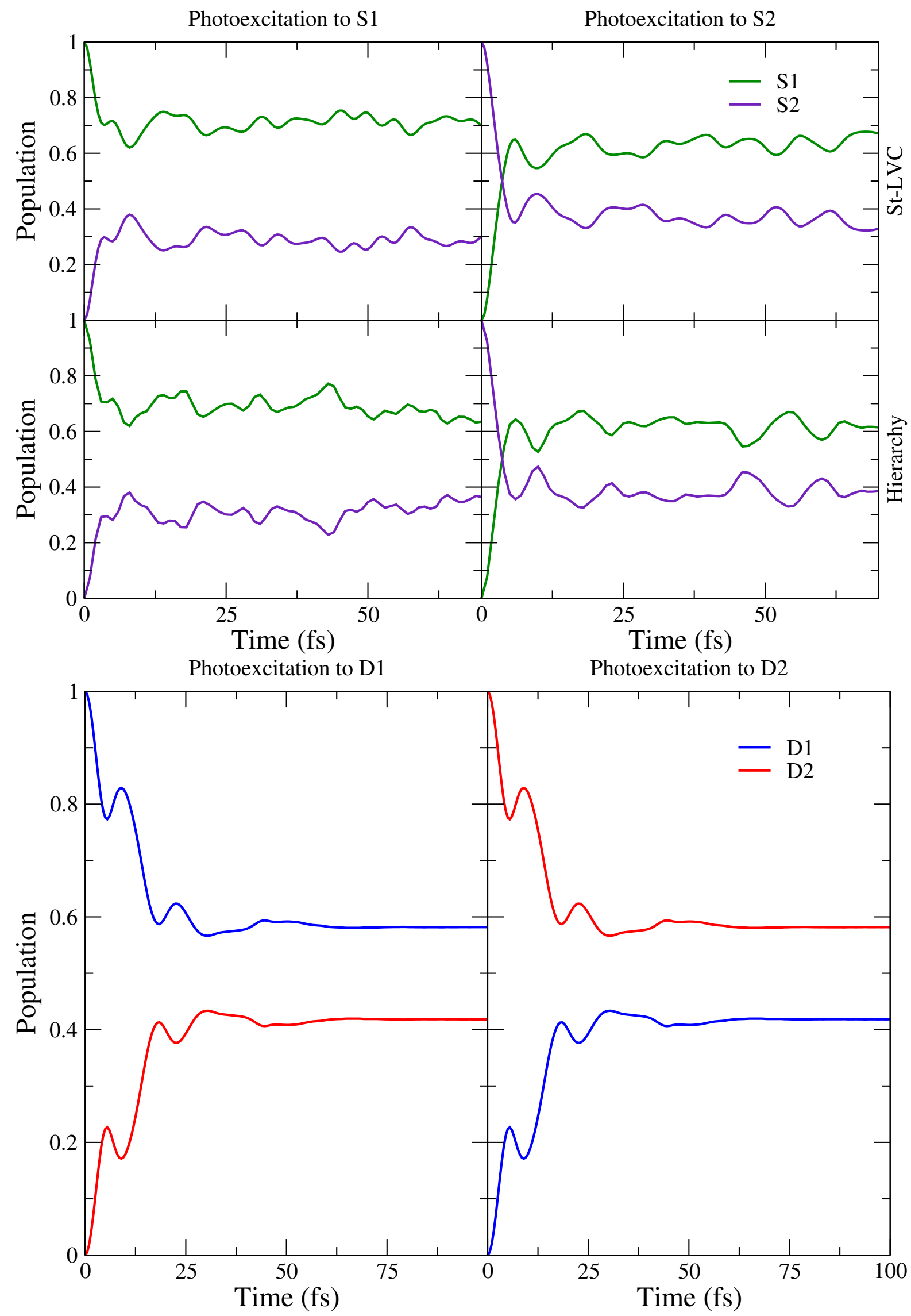

Figure S27: Population dynamics for (P_2)-**DES**$_2$. Top: Population dynamics after photoexcitation to S_1 and S_2 for the delocalized standard (St-LVC) and hierarchical (Hierarchy) Hamiltonians. Bottom: Population dynamics after photoexcitation to the degenerate states D_1 and D_2 for the localized hierarchical Hamiltonian. CAM-B3LYP/6-31G(d) level of theory.

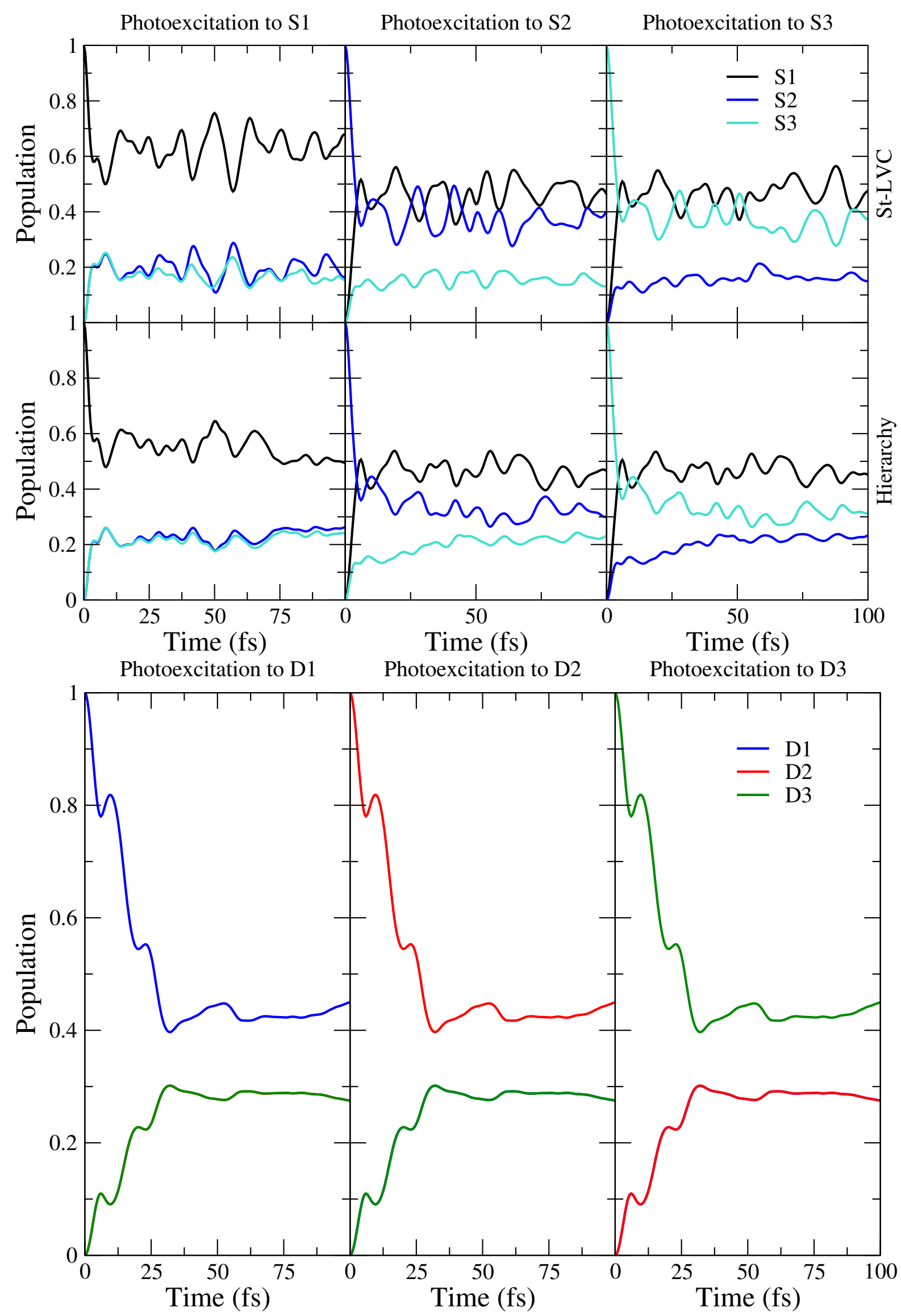

Figure S28: Population dynamics for (P_3)-**DES**$_3$. Top: Population dynamics after photoexcitation to S_1, S_2 and S_3 for the delocalized standard (St-LVC) and hierarchical (Hierarchy) Hamiltonians. Bottom: Population dynamics after photoexcitation to the degenerate states D_1, D_2 and D_3 for the localized hierarchical Hamiltonian. CAM-B3LYP/6-31G(d) level of theory.

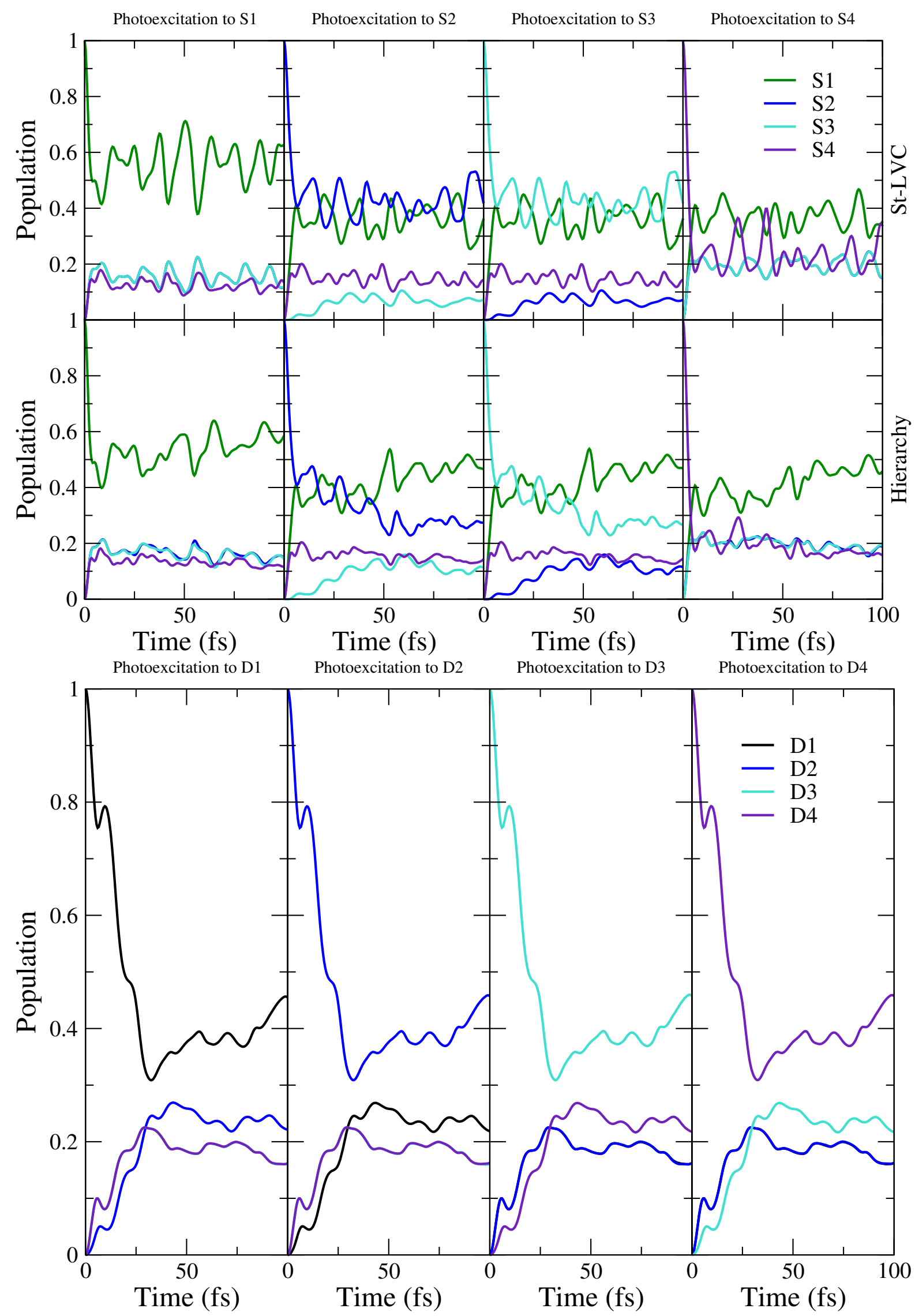

Figure S29: Population dynamics for (P_4)-$\mathbf{DES}_4$. Top: Population dynamics after photoexcitation to S_1, S_2, S_3 and S_4 for the delocalized standard (St-LVC) and hierarchical (Hierarchy) Hamiltonians. Bottom: Population dynamics after photoexcitation to the degenerate states D_1, D_2, D_3 and D_4 for the localized hierarchical Hamiltonian. CAM-B3LYP/6-31G(d) level of theory.

7 Further results with the delocalized St-LVC model

7.1 Block convergence for the Hierarchy model

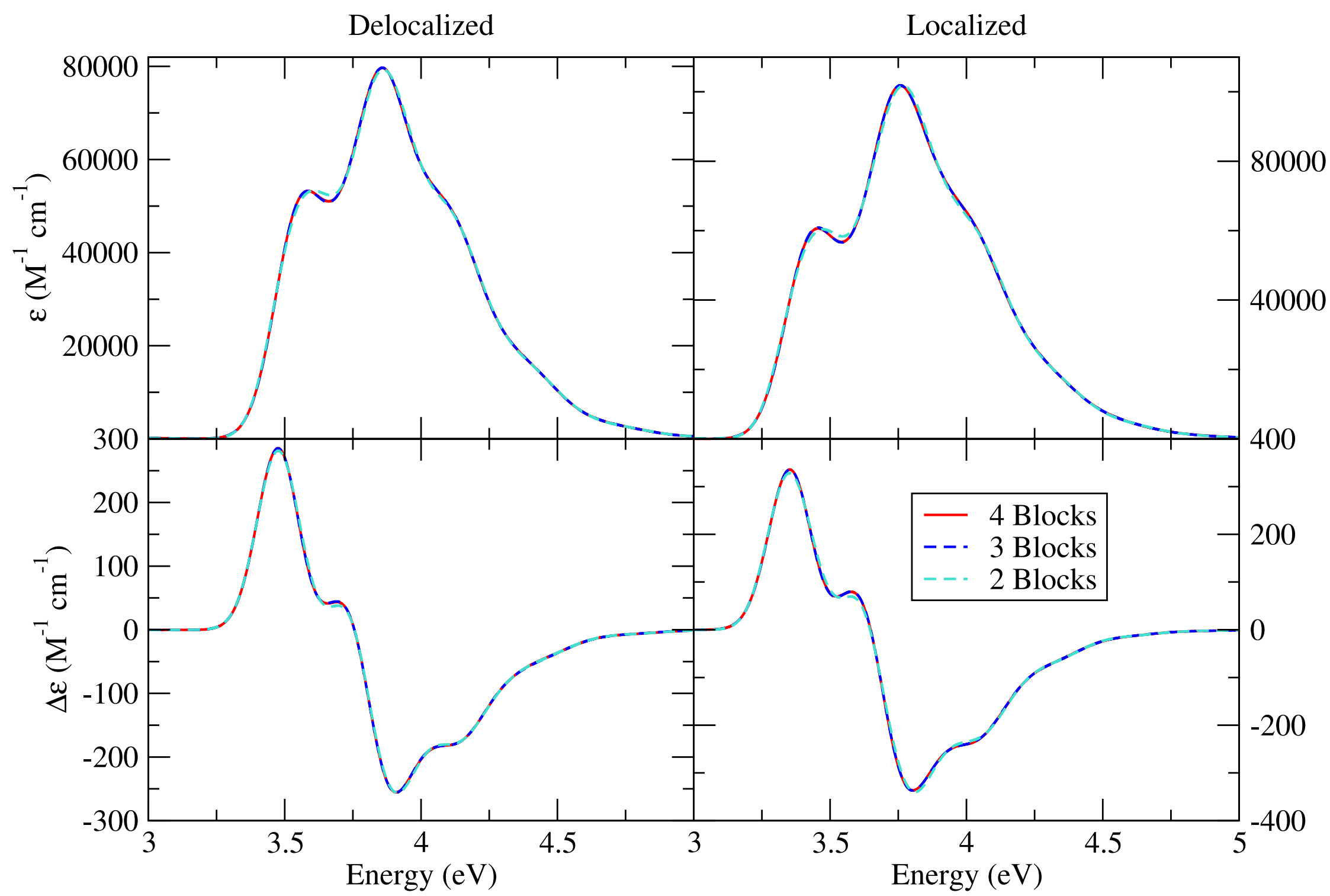

Figure S30: Comparison for Absorption and ECD spectra for (P_2)-**DES**$_2$ with different number of blocks for the Hierarchy methodology. Damping factor set at τ = 12.00 fs. CAM-B3LYP/6-31G(d) level of theory.

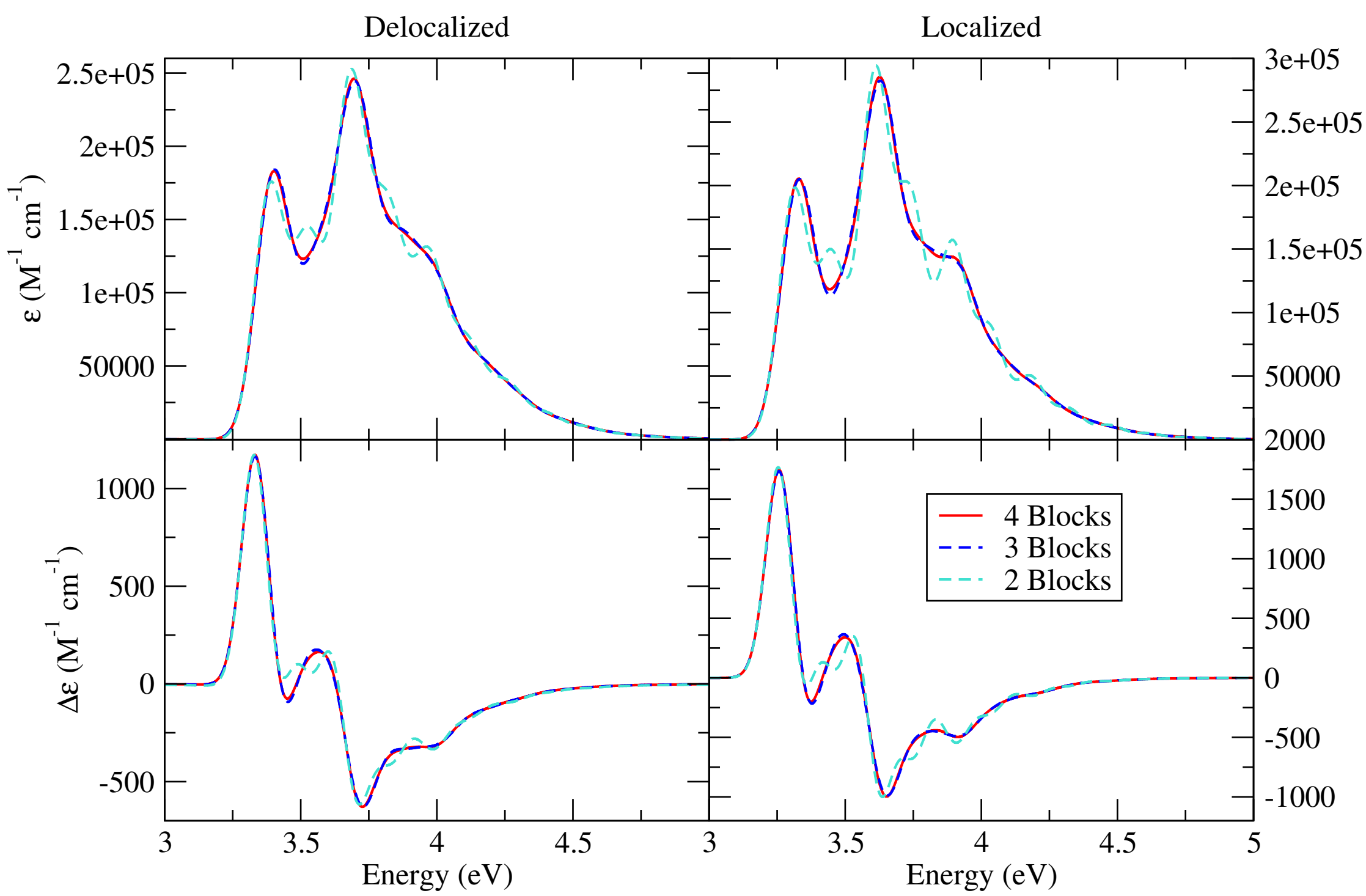

Figure S31: Comparison for Absorption and ECD spectra for (P_3)-$\mathbf{DES}_3$ with different number of blocks for the Hierarchy methodology. Damping factor set at τ = 18.26 fs. CAM-B3LYP/6-31G(d) level of theory.

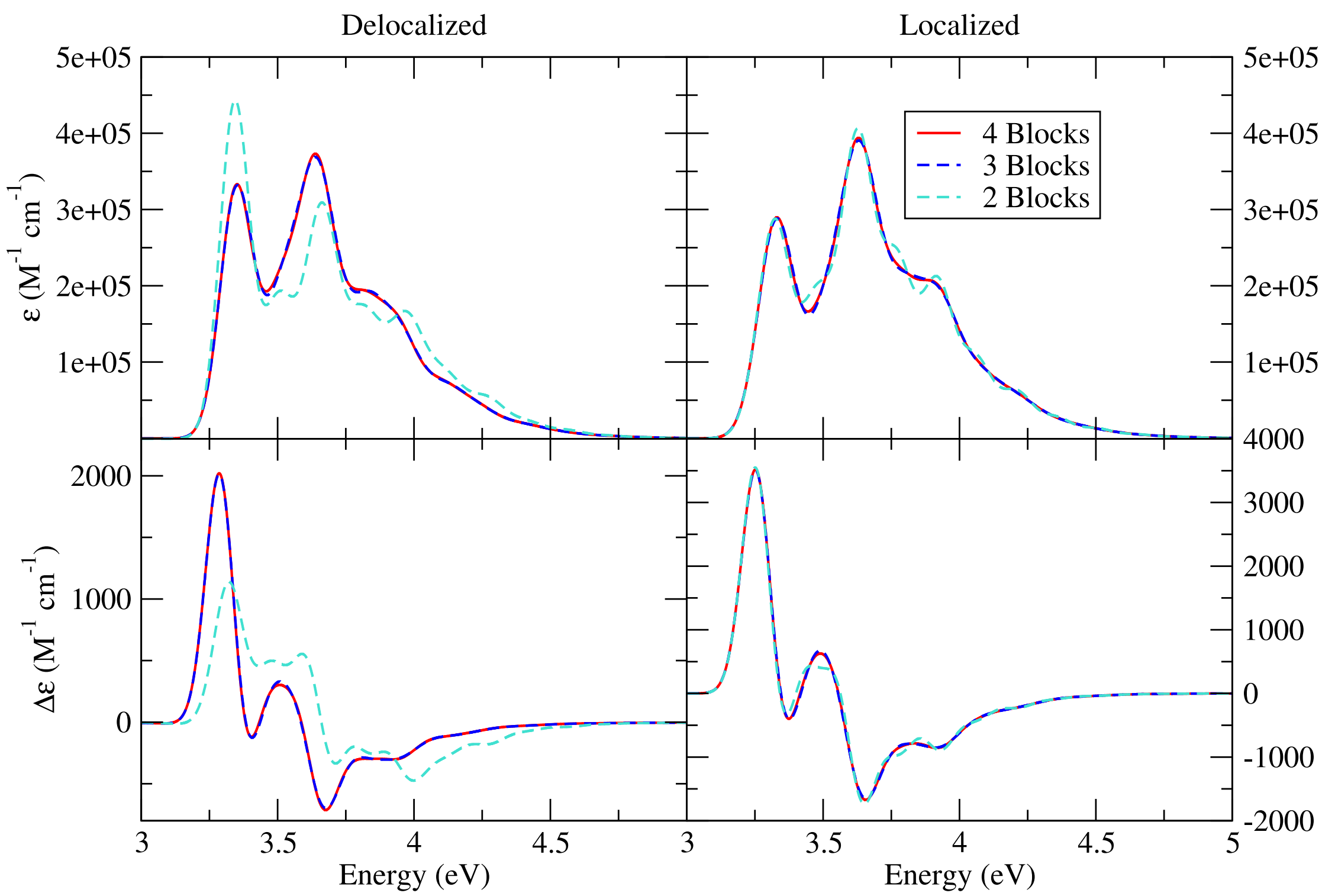

Figure S32: Comparison for Absorption and ECD spectra for (P_4)-**DES**$_4$ with different number of blocks for the Hierarchy methodology. Damping factor set at τ = 17.00 fs. CAM-B3LYP/6-31G(d) level of theory.

7.2 Comparison between Hierarchy and St-LVC methodologies

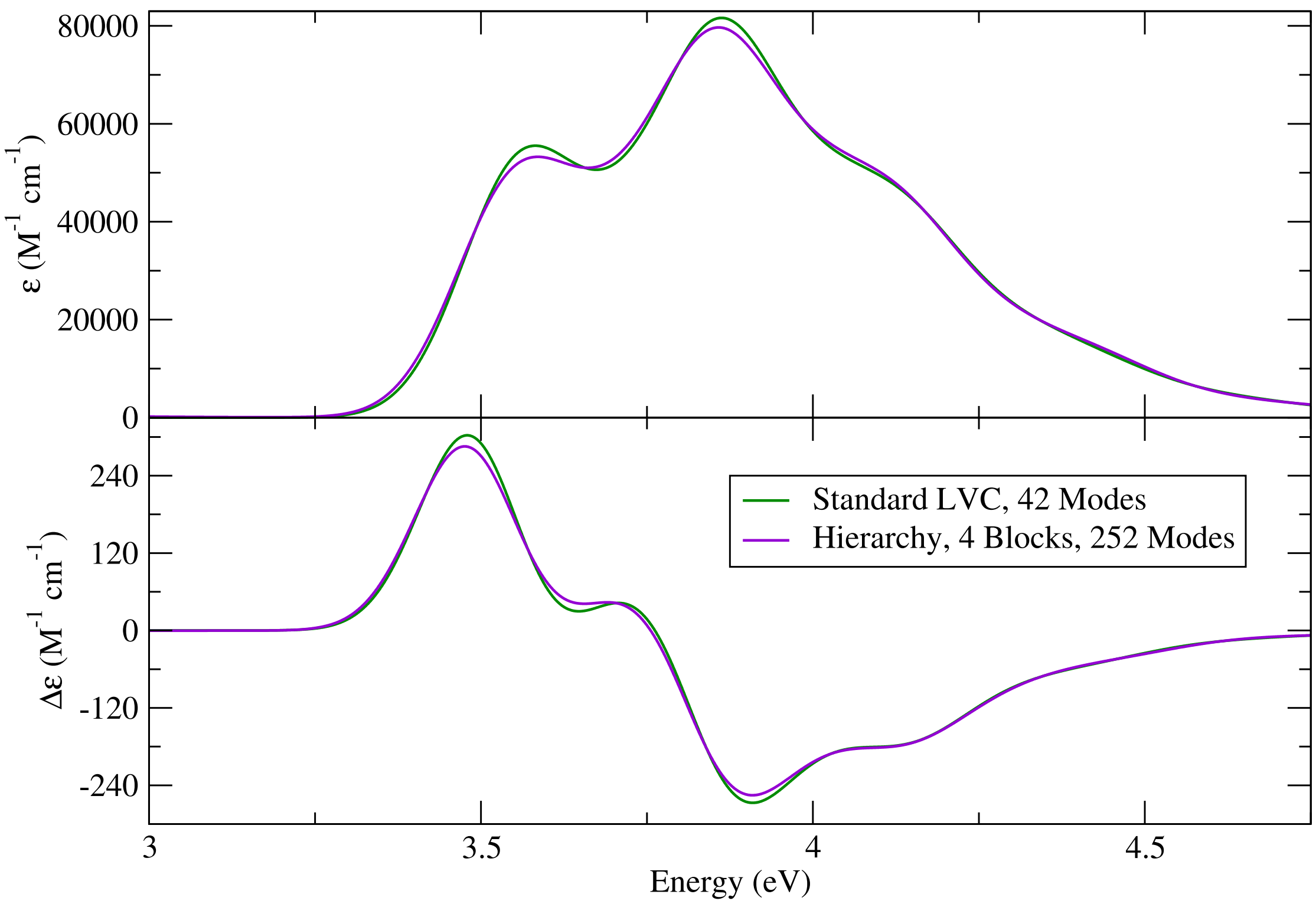

Figure S33: Comparison for Absorption and ECD spectra for (P_2)-**DES**$_2$ with Standard and hierarchy methodologies. τ = 12.0 fs for both Absorption and ECD. CAM-B3LYP/6-31G(d) level of theory.

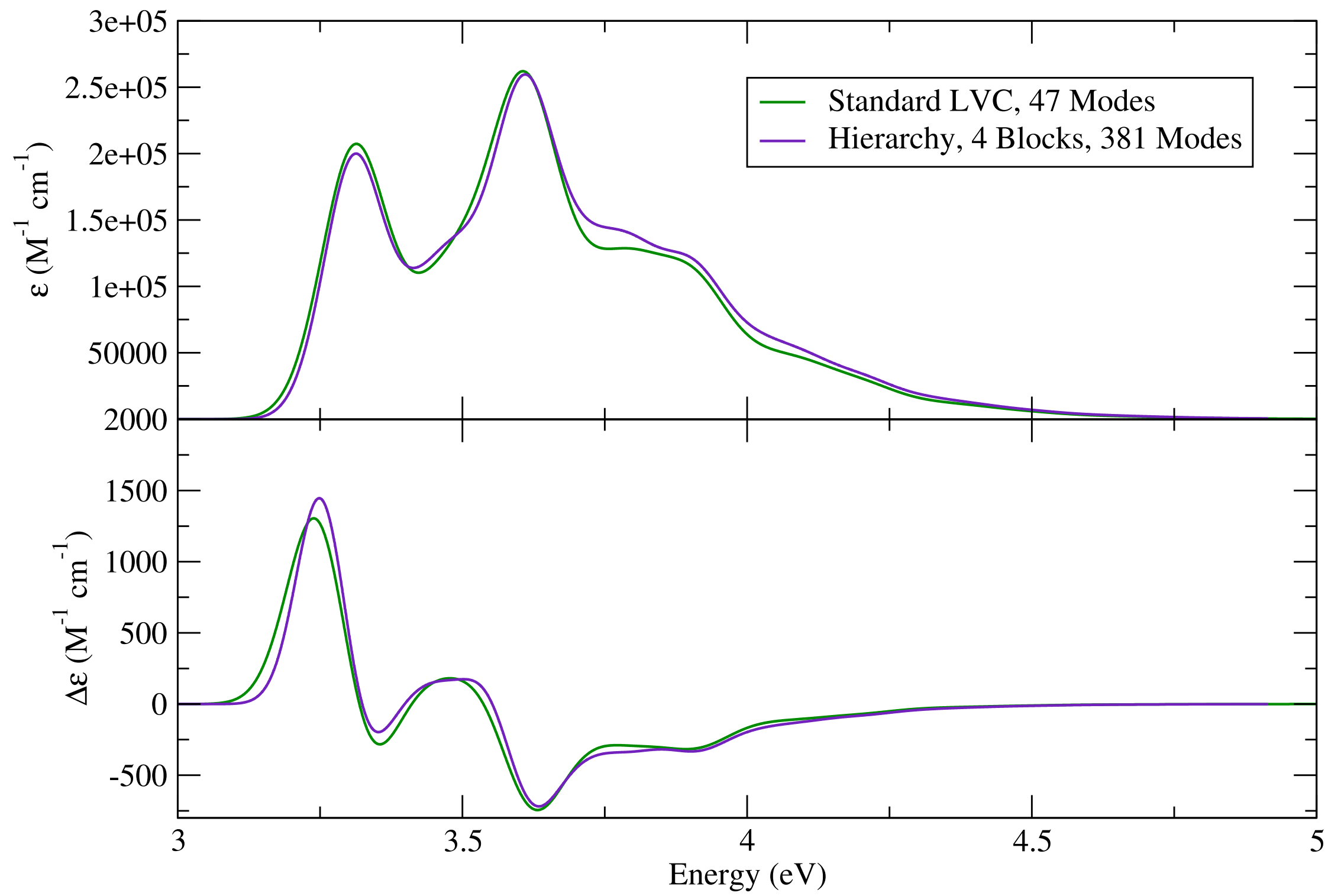

Figure S34: Comparison for Absorption and ECD spectra for (P_3)-$\mathbf{DES}_3$ with Standard and Hierarchy methodologies. τ = 17.0 fs for St-LVC and 22.0 fs for Hierarchy St-LVC. CAM-B3LYP/6-31G(d) level of theory.

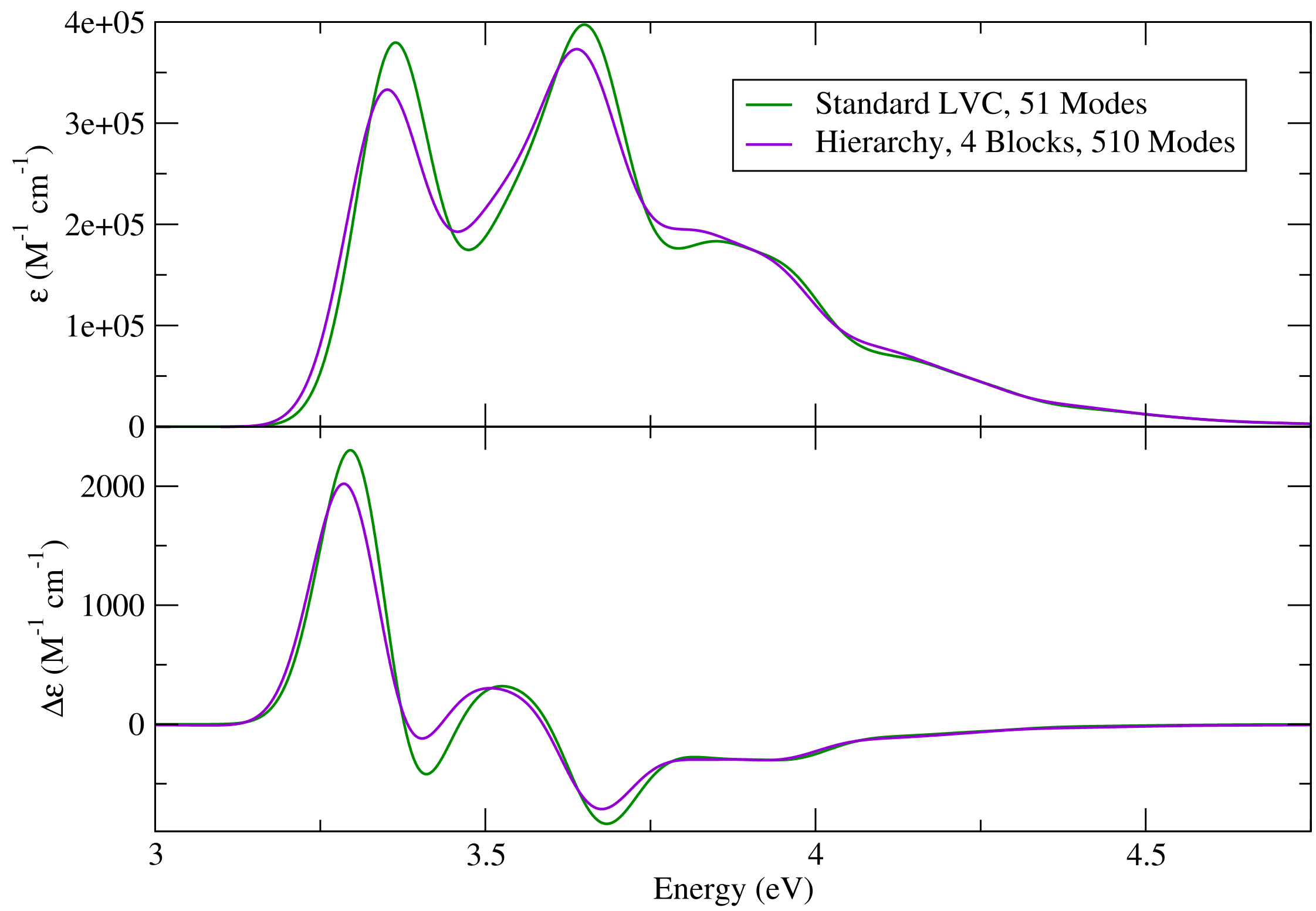

Figure S35: Comparison for Absorption and ECD spectra for (P_4)-**DES**$_4$ with Standard and Hierarchy methodologies. τ = 17.0 fs for both Absorption and ECD. CAM-B3LYP/6-31G(d) level of theory.

7.3 Investigating the non-linear dependence of intensities on the number of fragments

Traces of the non-linear increase in ECD intensities with respect to the number of fragments n for the computed spectra are observed by a simple analysis of the dipole (Dip Str) and rotatory strengths (R). For S_1, R is 1700 10^{-40}, 3900 10^{-40} and 6800 10^{-40} cgs, respectively for (P_2)-**DES**$_2$, (P_3)-**DES**$_3$ and (P_4)-**DES**$_4$, with and increase of $\sim$ 2.3 and $\sim$ 2.0. The lowest band with negative R is due to S_2 for (P_2)-**DES**$_2$ with a value of -2000 10^{-40} cgs, and to the pair S_2/S_3 for (P_3)-**DES**$_3$ and (P_4)-**DES**$_4$ with total R -4100 10^{-40} and -6200 10^{-40} cgs, with an increase of $\sim$ 2.05 and $\sim$ 1.5. The increase of the dipole strength is smaller for S_1 with a factor $\sim$1.7 from DES_2 to DES_3 and $\sim$1.4 from DES_3 to DES_4, and of $\sim$2.85 and $\sim$1.6 for the contributions of S_2 for (P_2)-**DES**$_2$ and of S_2/S_3 for (P_3)-**DES**$_3$ and (P_4)-**DES**$_4$.

The rationalization of this trend is simpler for the non degenerate state S_1 which arises, in all species, from the total symmetric combination of the local excitations with equal weights. The module of the ETDM of the monomer strongly increases from DES_2 to DES_4 (check **Table 3 in the SI**) due to the planarization of the monomer structure, but it tends to lie in the molecular plane XY. However, after mixing in the S_1 eigenstate, only the Z-component survives, due to the symmetry combination. As a consequence, S_1 transition remains weak for all species, although it moderately increases from DES_2 to DES_4. Differently, for ECD the MTDMs of all monomers are mainly polarized along Z and they sum up constructively. Furthermore, their external component grows because the distance of each monomer from the center of mass of the macrocycle increases (**Table 4 in the SI)**, explaining the increase of the rotatory strength with n.

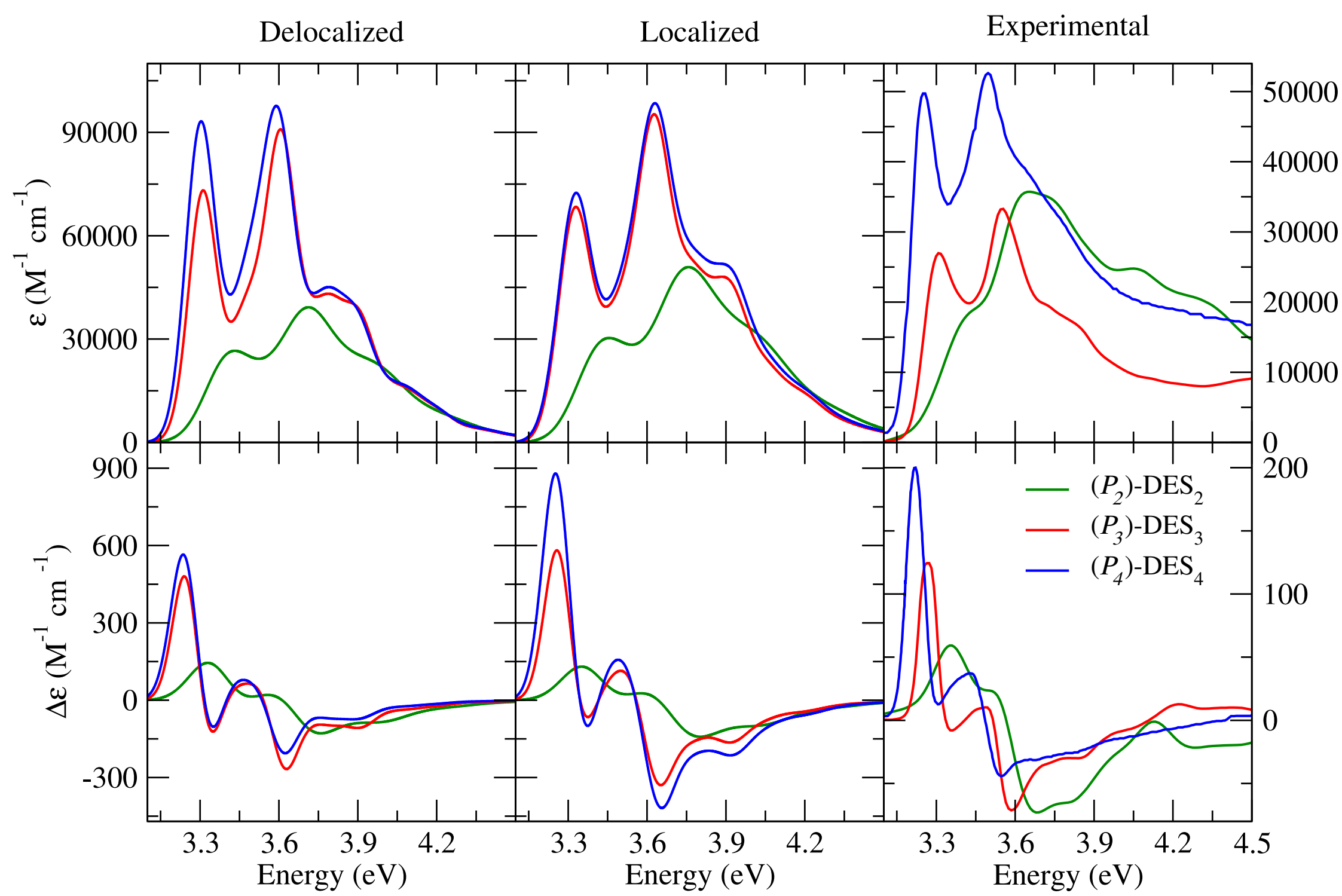

Figure S36: Comparison of OPA and ECD spectra with intensities divided by the number of fragments integrating the macrocycle: 2 for (P_2)-**DES**$_2$, 3 for (P_3)-**DES**$_3$ and 4 for (P_4)-**DES**$_4$. The same Y axis was defined for both Delocalized and Localized. CAM-B3LYP/6-31G(d) level of theory.

7.4 Broadening related to vibronic progressions

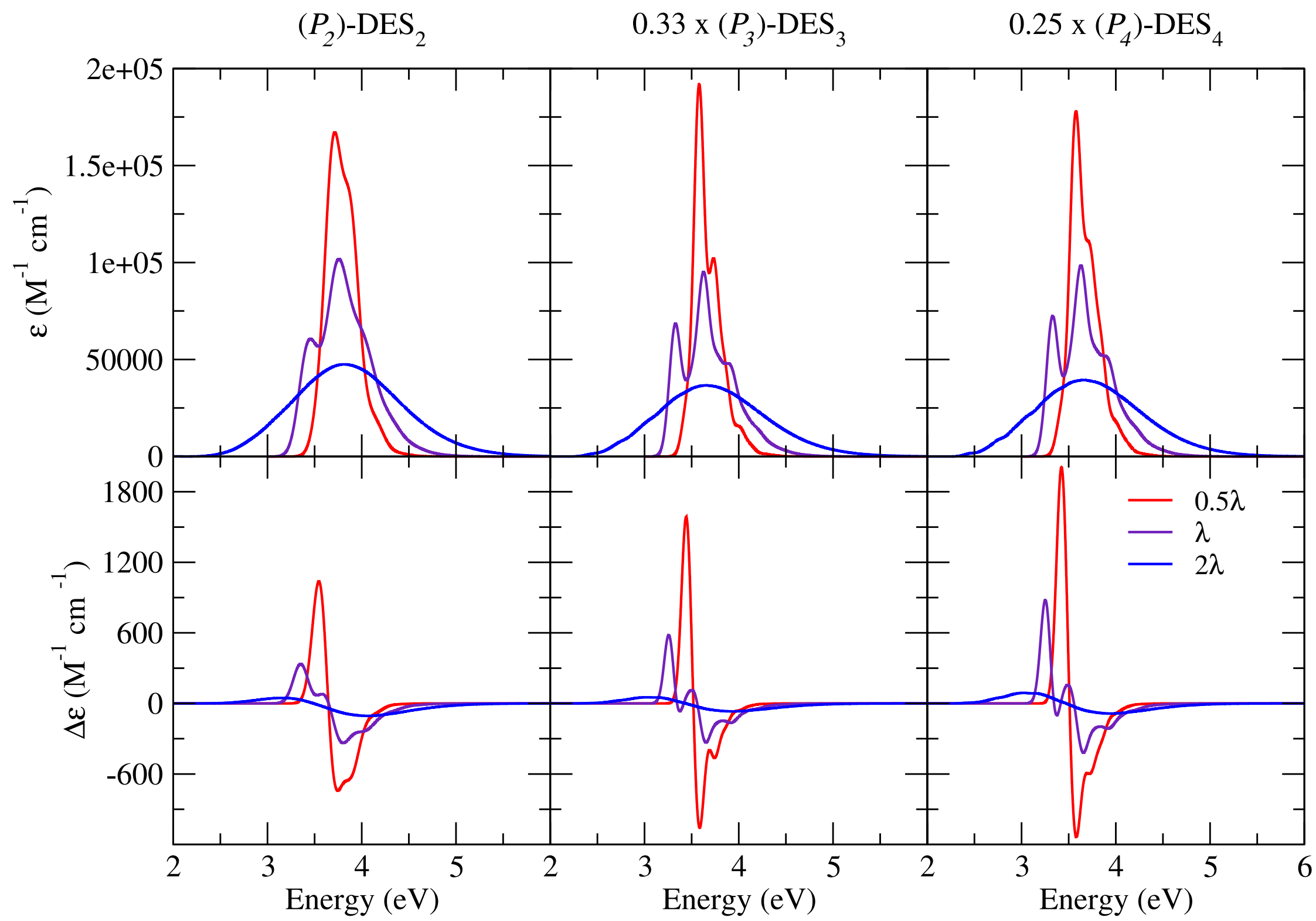

Figure S37: Localized OPA and ECD spectra of the species studied in this work by multiplying λ by a factor of 0.5, 1.0 and 2.0. Spectra for (P_3)-**DES**$_3$ and (P_4)-**DES**$_4$ scaled by a factor of 0.33 and 0.25, respectively. CAM-B3LYP/6-31G(d) level of theory.

7.5 g-Factors

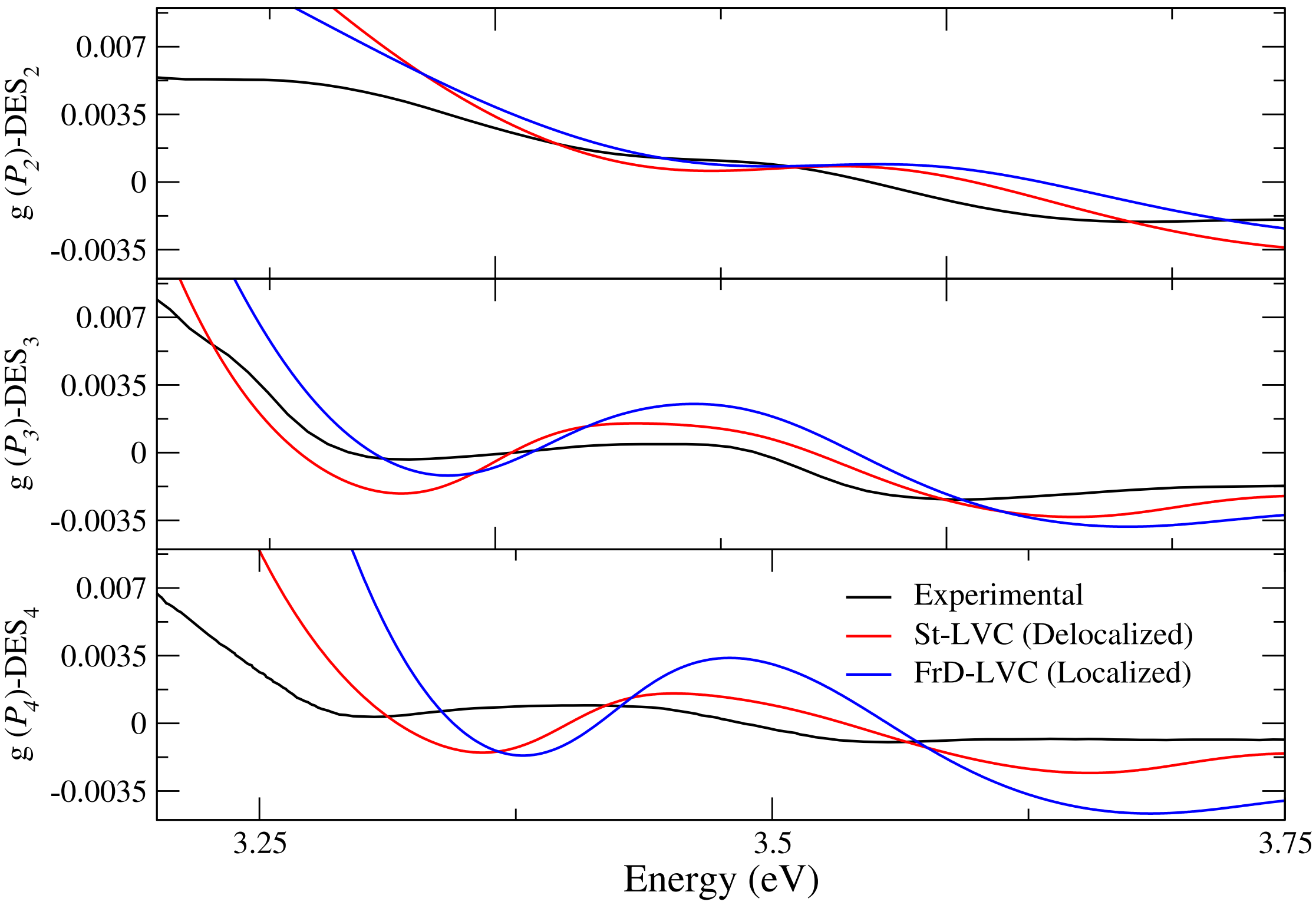

Figure S38: Experimental and computed g-factors along the most relevant energy range for the spectra for the species studied in this work. CAM-B3LYP/6-31G(d) level of theory.

8 Movies of the main vibrational modes

Movies of the main vibrational modes of the three species (P_2)-**DES**$_2$, (P_3)-**DES**$_3$ and (P_4)-**DES**$_4$ are also provided as separated supporting information files and here we report the corresponding captions

- **Movie01-DES2_214**: Vibrational Mode 214 of (P_2)-**DES**$_2$, frequency 1698.8 cm^{-1}
- **Movie02-DES2_216**: Vibrational Mode 216 of (P_2)-**DES**$_2$, frequency 1700.7 cm^{-1}
- **Movie03-DES2_219**: Vibrational Mode 219 of (P_2)-**DES**$_2$, frequency 1705.5 cm^{-1}
- **Movie04-DES2_223**: Vibrational Mode 223 of (P_2)-**DES**$_2$, frequency 2392.7 cm^{-1}
- **Movie05-DES2_224**: Vibrational Mode 224 of (P_2)-**DES**$_2$, frequency 2392.8 cm^{-1}
- **Movie06-DES3_337**: Vibrational Mode 337 of (P_3)-**DES**$_3$, frequency 2401.0 cm^{-1}
- **Movie07-DES3_337**: Vibrational Mode 338 of (P_3)-**DES**$_3$, frequency 2401.0 cm^{-1}
- **Movie08-DES3_337**: Vibrational Mode 339 of (P_3)-**DES**$_3$, frequency 2401.0 cm^{-1}
- **Movie09-DES4_451**: Vibrational Mode 451 of (P_4)-**DES**$_4$, frequency 2400.2 cm^{-1}
- **Movie10-DES4_452**: Vibrational Mode 452 of (P_4)-**DES**$_4$, frequency 2400.3 cm^{-1}
- **Movie11-DES4_453**: Vibrational Mode 453 of (P_4)-**DES**$_4$, frequency 2400.3 cm^{-1}
- **Movie12-DES4_454**: Vibrational Mode 454 of (P_4)-**DES**$_4$, frequency 2400.3 cm^{-1}

References

(S1) Jurinovich, S.; Guido, C. A.; Bruhn, T.; Pescitelli, G.; Mennucci, B. The role of magnetic–electric coupling in exciton-coupled ECD spectra: the case of bis-phenanthrenes. *Chem. Commun.* **2015**, *51*, 10498–10501.

(S2) Jurinovich, S.; Cupellini, L.; Guido, C. A.; Mennucci, B. EXAT: EXcitonic Analysis Tool. *J. Comput. Chem.* **2018**, *39*, 279–286.
