# Supplementary material for: Localized and Delocalized Nonadiabatic Vibronic Models Unravel Chiroptical Trends and Design Principles in Symmetric π Conjugated Macrocycles
Source: J Chem Theory Comput. 2026 Oct 19;22(21):11484–97. doi: 10.1021/acs.jctc.6c01519 (PMC13757766; doi:10.1021/acs.jctc.6c01519)
Supplement: Supplementary file 2 [file ct6c01519_si_001.zip › Movies/Caption_movies.docx]

Movies of the main vibrational modes of the three species (*P_2_*)- **DES**_2_, (*P_3_*)- **DES**_3_ and (*P_4_*)- **DES**_4_ are also provided as separated supporting information files and here we report the corresponding captions

**Movie01-DES2_214**: Vibrational Mode 214 of (*P_2_*)- **DES**_2_, frequency 1698.8 cm^-1^

**Movie02-DES2_216:** Vibrational Mode 216 of (*P_2_*)- **DES**_2_, frequency 1700.7 cm^-1^

**Movie03-DES2_219:** Vibrational Mode 219 of (*P_2_*)- **DES**_2_, frequency 1705.5 cm^-1^

**Movie04-DES2_223:** Vibrational Mode 223 of (*P_2_*)- **DES**_2_, frequency 2392.7 cm^-1^

**Movie05-DES2_224:** Vibrational Mode 224 of (*P_2_*)- **DES**_2_, frequency 2392.8 cm^-1^

**Movie06-DES3_337:** Vibrational Mode 337 of (*P_3_*)- **DES**_3_, frequency 2401.0 cm^-1^

**Movie07-DES3_338:** Vibrational Mode 338 of (*P_3_*)- **DES**_3_, frequency 2401.0 cm^-1^

**Movie08-DES3_339:** Vibrational Mode 339 of (*P_3_*)- **DES**_3_, frequency 2401.0 cm^-1^

**Movie09-DES4_451:** Vibrational Mode 451 of (*P_4_*)- **DES**_4_, frequency 2400.2 cm^-1^

**Movie10-DES4_452:** Vibrational Mode 452 of (*P_4_*)- **DES**_4_, frequency 2400.3 cm^-1^

**Movie11-DES4_453:** Vibrational Mode 453 of (*P_4_*)- **DES**_4_, frequency 2400.3 cm^-1^

**Movie12-DES4_454:** Vibrational Mode 454 of (*P_4_*)- **DES**_4_, frequency 2400.3 cm^-1^
